# Supplementary material for: Tifcemalimab as monotherapy or in combination with toripalimab in patients with relapsed/refractory lymphoma: a Phase I trial
Source: Nat Commun. 2025 May 16;16:4559. doi: 10.1038/s41467-025-59461-3 (PMC12084519; doi:10.1038/s41467-025-59461-3)
Supplement: Supplementary file 1 — Supplementary Information [file 41467_2025_59461_MOESM1_ESM.pdf]

**Tifcemalimab as Monotherapy or in Combination with Toripalimab in Patients with Relapsed/Refractory Lymphoma: Phase I Trial**

Supplementary Table 1: Overall Summary of Treatment-Emergent Adverse Events (Part A) ....2
Supplementary Table 2: Treatment-Related Adverse Events by Grade (Part A).......3
Supplementary Table 3: Overall Summary of Treatment-Emergent Adverse Events (Part B) ....5
Supplementary Table 4: Treatment-Related Adverse Events by Grade (Part B).......6
Supplementary Table 5: Treatment-Related Adverse Events Leading to Treatment Discontinuation (Part B) ....10
Supplementary Table 6: Receptor Occupancy (%) of T and B cells [Mean±SD (N)] in Part A....11
Supplementary Table 7: Summary of PK Parameters of Tifcemalimab Monotherapy and Tifcemalimab in Combination with Toripalimab Following Single Dose and Multiple Doses in Patients with Relapsed/Refractory Lymphoma....12
Supplementary Table 8: The Ratio of Cytokines Concentration 24 Hours Post-Administration Compared to Pre-Dose Levels....13
Supplementary Table 9: Summary of Tumor Response for Monotherapy, Combination Therapy and Classical Hodgkin's Lymphoma (Full Analysis Set) ....14
Supplementary Table 10 Herpes Virus Entry Mediator (HVEM) and Programmed Cell Death Ligand 1 (PD-L1) Staining ....15
Supplementary Figure 1: Serum Concentration of Tifcemalimab after First Dosing ....16
Supplementary Figure 2: Progression-Free Survival Status....17
Supplementary Figure 3 Tumor response ....19
Supplementary Figure 4: Representative Immunohistochemistry Staining of HVEM in Patients with Lymphoma....21

## Supplementary Table 1: Overall Summary of Treatment-Emergent Adverse Events (Part A)

| | Part A (Tifcemalimab Monotherapy) | | | | |
|---|---|---|---|---|---|
| | **1mg/kg (N=3)** | **3mg/kg (N=12)** | **10mg/kg (N=3)** | **200mg (N=7)** | **Total (N=25)** |
| **Adverse Events Type** | **n (%)** | **n (%)** | **n (%)** | **n (%)** | **n (%)** |
| Treatment-emergent adverse event (TEAE) | 3 (100) | 11 (91·7) | 3 (100) | 6 (85·7) | 23 (92·0) |
| Treatment-related adverse event (TRAE) | 2 (66·7) | 11 (91·7) | 1 (33·3) | 6 (85·7) | 20 (80·0) |
| Dose-limiting toxicities (DLT) | 0 | 0 | 0 | 0 | 0 |
| Grade ≥ 3 TEAE | 0 | 3 (25·0) | 0 | 2 (28·6) | 5 (20·0) |
| Grade ≥ 3 TRAE | 0 | 1 (8·3) | 0 | 2 (28·6) | 3 (12·0) |
| Serious adverse event (SAE) | 0 | 2 (16·7) | 0 | 0 | 2 (8·0) |
| Study drug-related SAE | 0 | 0 | 0 | 0 | 0 |
| Immune-related adverse events (irAE) | 1 (33·3) | 6 (50·0) | 2 (66·7) | 1 (14·3) | 10 (40·0) |
| Grade ⩾ 3 irAE | 0 | 0 | 0 | 0 | 0 |
| TEAE leading to study drug interruption | 1 (33·3) | 2 (16·7) | 0 | 0 | 3 (12·0) |
| TRAE leading to study drug interruption | 1 (33·3) | 1 (8·3) | 0 | 0 | 2 (8·0) |
| TEAE leading to study drug discontinuation | 0 | 0 | 0 | 0 | 0 |
| TEAE leading to death | 0 | 0 | 0 | 0 | 0 |

Treatment-related adverse events (TRAEs) were defined as TEAEs that were related or unlikely related to the study treatment as assessed by the investigator.

## Supplementary Table 2: Treatment-Related Adverse Events by Grade (Part A)

| | Part A (Tifcemalimab Monotherapy) | | | | | | | | | |
|---|---|---|---|---|---|---|---|---|---|---|
| | JS004 1 mg/kg (N=3) n (%) | | JS004 3 mg/kg (N=12) n (%) | | JS004 10 mg/kg (N=3) n (%) | | JS004 200 mg (N=7) n (%) | | Total (N=25) n (%) | |
| **Preferred term** | **Grade 1-2** | **Grade 3-4** | **Grade 1-2** | **Grade 3-4** | **Grade 1-2** | **Grade 3-4** | **Grade 1-2** | **Grade 3-4** | **Grade 1-2** | **Grade 3-4** |
| Any TRAEs | 2 (66.7) | 0 | 10 (83.3) | 1 (8.3) | 1 (33.3) | 0 | 4 (57.1) | 2 (28.6) | 17 (68.0) | 3 (12.0) |
| Anemia | 0 | 0 | 2 (16.7) | 0 | 0 | 0 | 2 (28.6) | 1 (14.3) | 4 (16.0) | 1 (4.0) |
| Lymphocyte count decreased | 0 | 0 | 1 (8.3) | 0 | 0 | 0 | 1 (14.3) | 1 (14.3) | 2 (8.0) | 1 (4.0) |
| Blood bilirubin increased | 0 | 0 | 0 | 1 (8.3) | 0 | 0 | 1 (14.3) | 0 | 1 (4.0) | 1 (4.0) |
| γ-glutamyltransferase increased | 0 | 0 | 0 | 1 (8.3) | 0 | 0 | 0 | 0 | 0 | 1 (4.0) |
| White blood cell count decreased | 0 | 0 | 2 (16.7) | 0 | 0 | 0 | 2 (28.6) | 0 | 4 (16.0) | 0 |
| Pyrexia | 1 (33.3) | 0 | 0 | 0 | 1 (33.3) | 0 | 2 (28.6) | 0 | 4 (16.0) | 0 |
| Asthenia | 0 | 0 | 3 (25.0) | 0 | 0 | 0 | 0 | 0 | 3 (12.0) | 0 |
| Aspartate aminotransferase increased | 0 | 0 | 3 (25.0) | 0 | 0 | 0 | 0 | 0 | 3 (12.0) | 0 |
| Bilirubin conjugated increased | 0 | 0 | 0 | 0 | 0 | 0 | 2 (28.6) | 0 | 2 (8.0) | 0 |
| Protein urine present | 0 | 0 | 2 (16.7) | 0 | 0 | 0 | 0 | 0 | 2 (8.0) | 0 |
| Blood bilirubin unconjugated increased | 0 | 0 | 1 (8.3) | 0 | 0 | 0 | 1 (14.3) | 0 | 2 (8.0) | 0 |
| Platelet count decreased | 0 | 0 | 1 (8.3) | 0 | 0 | 0 | 1 (14.3) | 0 | 2 (8.0) | 0 |
| Blood pressure increased | 2 (66.7) | 0 | 0 | 0 | 0 | 0 | 0 | 0 | 2 (8.0) | 0 |
| Proteinuria | 0 | 0 | 1 (8.3) | 0 | 0 | 0 | 1 (14.3) | 0 | 2 (8.0) | 0 |
| Hypothyroidism | 0 | 0 | 2 (16.7) | 0 | 0 | 0 | 0 | 0 | 2 (8.0) | 0 |
| Sinus tachycardia | 0 | 0 | 1 (8.3) | 0 | 0 | 0 | 1 (14.3) | 0 | 2 (8.0) | 0 |
| White blood cell count increased | 0 | 0 | 0 | 0 | 0 | 0 | 1 (14.3) | 0 | 1 (4.0) | 0 |
| Alanine aminotransferase increased | 0 | 0 | 1 (8.3) | 0 | 0 | 0 | 0 | 0 | 1 (4.0) | 0 |
| Low density lipoprotein increased | 0 | 0 | 0 | 0 | 0 | 0 | 1 (14.3) | 0 | 1 (4.0) | 0 |
| Red blood cell count decreased | 0 | 0 | 0 | 0 | 0 | 0 | 1 (14.3) | 0 | 1 (4.0) | 0 |
| Anti-thyroid antibody positive | 0 | 0 | 1 (8.3) | 0 | 0 | 0 | 0 | 0 | 1 (4.0) | 0 |
| Bilirubin urine present | 0 | 0 | 1 (8.3) | 0 | 0 | 0 | 0 | 0 | 1 (4.0) | 0 |
| Blood pressure systolic increased | 0 | 0 | 1 (8.3) | 0 | 0 | 0 | 0 | 0 | 1 (4.0) | 0 |
| Weight increased | 0 | 0 | 0 | 0 | 0 | 0 | 1 (14.3) | 0 | 1 (4.0) | 0 |
| Electrocardiogram QRS complex abnormal | 0 | 0 | 1 (8.3) | 0 | 0 | 0 | 0 | 0 | 1 (4.0) | 0 |
| Electrocardiogram QRS prolonged | 0 | 0 | 0 | 0 | 1 (33.3) | 0 | 0 | 0 | 1 (4.0) | 0 |
| Electrocardiogram ST-T change | 0 | 0 | 1 (8.3) | 0 | 0 | 0 | 0 | 0 | 1 (4.0) | 0 |

| | Part A (Tifcemalimab Monotherapy) | | | | | | | | | |
|---|---|---|---|---|---|---|---|---|---|---|
| | JS004 1 mg/kg (N=3) n (%) | | JS004 3 mg/kg (N=12) n (%) | | JS004 10 mg/kg (N=3) n (%) | | JS004 200 mg (N=7) n (%) | | Total (N=25) n (%) | |
| **Preferred term** | **Grade 1-2** | **Grade 3-4** | **Grade 1-2** | **Grade 3-4** | **Grade 1-2** | **Grade 3-4** | **Grade 1-2** | **Grade 3-4** | **Grade 1-2** | **Grade 3-4** |
| Blood alkaline phosphatase increased | 0 | 0 | 1 (8.3) | 0 | 0 | 0 | 0 | 0 | 1 (4.0) | 0 |
| Blood fibrinogen decreased | 0 | 0 | 0 | 0 | 0 | 0 | 1 (14.3) | 0 | 1 (4.0) | 0 |
| Thyroxine free increased | 0 | 0 | 0 | 0 | 0 | 0 | 1 (14.3) | 0 | 1 (4.0) | 0 |
| Neutrophil count increased | 0 | 0 | 0 | 0 | 0 | 0 | 1 (14.3) | 0 | 1 (4.0) | 0 |
| Hypercholesterolemia | 0 | 0 | 0 | 0 | 0 | 0 | 1 (14.3) | 0 | 1 (4.0) | 0 |
| Herpes zoster | 1 (33.3) | 0 | 0 | 0 | 0 | 0 | 0 | 0 | 1 (4.0) | 0 |
| Upper respiratory tract infection | 0 | 0 | 1 (8.3) | 0 | 0 | 0 | 0 | 0 | 1 (4.0) | 0 |
| Infusion related reaction | 1 (33.3) | 0 | 0 | 0 | 0 | 0 | 0 | 0 | 1 (4.0) | 0 |
| Back pain | 0 | 0 | 1 (8.3) | 0 | 0 | 0 | 0 | 0 | 1 (4.0) | 0 |
| Groin pain | 0 | 0 | 1 (8.3) | 0 | 0 | 0 | 0 | 0 | 1 (4.0) | 0 |
| Pain in extremity | 0 | 0 | 1 (8.3) | 0 | 0 | 0 | 0 | 0 | 1 (4.0) | 0 |
| Dyspnoea | 0 | 0 | 1 (8.3) | 0 | 0 | 0 | 0 | 0 | 1 (4.0) | 0 |
| Nausea | 0 | 0 | 1 (8.3) | 0 | 0 | 0 | 0 | 0 | 1 (4.0) | 0 |
| Abdominal pain lower | 0 | 0 | 1 (8.3) | 0 | 0 | 0 | 0 | 0 | 1 (4.0) | 0 |
| Supraventricular extrasystoles | 0 | 0 | 1 (8.3) | 0 | 0 | 0 | 0 | 0 | 1 (4.0) | 0 |

## Supplementary Table 3: Overall Summary of Treatment-Emergent Adverse Events (Part B)

| Adverse Events Types | Part B (Tifcemalimab in Combination with Toripalimab) | | |
|---|---|---|---|
| | Tifcemalimab 100mg (N=10) | Tifcemalimab 200mg (N=36) | Total (N=46) |
| | n (%) | n (%) | n (%) |
| Treatment-emergent adverse event (TEAE) | 8 (80·0) | 36 (100) | 44 (95·7) |
| Treatment-related adverse event (TRAE) | 8 (80·0) | 34 (94·4) | 42 (91·3) |
| Dose-limiting toxicities (DLT) | 0 | 0 | 0 |
| Grade ≥ 3 TEAE | 2 (20·0) | 16 (44·4) | 18 (39·1) |
| Grade ≥ 3 TRAE | 1 (10·0) | 14 (38·9) | 15 (32·6) |
| Serious adverse event (SAE) | 1 (10·0) | 11 (30·6) | 12 (26·1) |
| Study drug-related SAE | 1 (10·0) | 9 (25·0) | 10 (21·7) |
| Immune-related adverse events (irAE) | 7 (70·0) | 18 (50·0) | 25 (54·3) |
| Grade ≥ 3 irAE | 0 | 4 (11·1) | 4 (8·7) |
| TEAE leading to study drug interruption | 3 (30·0) | 23 (63·9) | 26 (56·5) |
| TRAE leading to study drug interruption | 1 (10·0) | 13 (36·1) | 14 (30·4) |
| TEAE leading to study drug discontinuation | 0 | 6 (16·7) | 6 (13·0) |
| TRAE leading to study drug discontinuation | 0 | 6 (16·7) | 6 (13·0) |
| TEAE leading to death | 0 | 0 | 0 |

Treatment-related adverse events (TRAEs) were defined as TEAEs that were related or unlikely related to the study treatment as assessed by the investigator.

## Supplementary Table 4: Treatment-Related Adverse Events by Grade (Part B)

| Adverse events | Part B (Tifcemalimab in Combination with Toripalimab) | | | | | |
|---|---|---|---|---|---|---|
| | JS004 100mg+JS001 240 mg (N=10) n (%) | | JS004 200mg+JS001 240 mg (N=36) n (%) | | Total (N=46) n (%) | |
| | Grade 1-2 | Grade 3-4 | Grade 1-2 | Grade 3-4 | Grade 1-2 | Grade 3-4 |
| Any TRAEs | 7 (70.0) | 1 (10.0) | 20 (55.6) | 14 (38.9) | 27 (58.7) | 15 (32.6) |
| Pneumonia | 0 | 1 (10.0) | 4 (11.1) | 1 (2.8) | 4 (8.7) | 2 (4.3) |
| Infection | 0 | 0 | 0 | 1 (2.8) | 0 | 1 (2.2) |
| Sepsis | 0 | 0 | 0 | 1 (2.8) | 0 | 1 (2.2) |
| Pneumonia bacterial | 0 | 0 | 0 | 1 (2.8) | 0 | 1 (2.2) |
| Bronchitis | 0 | 0 | 0 | 1 (2.8) | 0 | 1 (2.2) |
| Tonsillitis | 0 | 0 | 1 (2.8) | 0 | 1 (2.2) | 0 |
| Herpes zoster | 0 | 0 | 1 (2.8) | 0 | 1 (2.2) | 0 |
| Respiratory tract infection | 0 | 0 | 1 (2.8) | 0 | 1 (2.2) | 0 |
| Urinary tract infection | 0 | 0 | 1 (2.8) | 0 | 1 (2.2) | 0 |
| Upper respiratory tract infection | 1 (10.0) | 0 | 5 (13.9) | 0 | 6 (13.0) | 0 |
| Pneumocystis jirovecii pneumonia | 0 | 0 | 1 (2.8) | 0 | 1 (2.2) | 0 |
| Herpes virus infection | 0 | 0 | 1 (2.8) | 0 | 1 (2.2) | 0 |
| Pyrexia | 2 (20.0) | 0 | 5 (13.9) | 2 (5.6) | 7 (15.2) | 2 (4.3) |
| Oedema peripheral | 0 | 0 | 0 | 1 (2.8) | 0 | 1 (2.2) |
| Asthenia | 1 (10.0) | 0 | 3 (8.3) | 0 | 4 (8.7) | 0 |
| Temperature intolerance | 0 | 0 | 1 (2.8) | 0 | 1 (2.2) | 0 |
| Influenza like illness | 0 | 0 | 1 (2.8) | 0 | 1 (2.2) | 0 |
| Facial pain | 0 | 0 | 1 (2.8) | 0 | 1 (2.2) | 0 |
| Chest discomfort | 0 | 0 | 2 (5.6) | 0 | 2 (4.3) | 0 |
| Hepatic function abnormal | 2 (20.0) | 0 | 2 (5.6) | 1 (2.8) | 4 (8.7) | 1 (2.2) |
| Immune-mediated hepatitis | 0 | 0 | 0 | 1 (2.8) | 0 | 1 (2.2) |
| Pneumonitis | 0 | 0 | 2 (5.6) | 1 (2.8) | 2 (4.3) | 1 (2.2) |
| Pleurisy | 0 | 0 | 0 | 1 (2.8) | 0 | 1 (2.2) |
| Dyspnoea | 0 | 0 | 2 (5.6) | 0 | 2 (4.3) | 0 |
| Interstitial lung disease | 0 | 0 | 1 (2.8) | 0 | 1 (2.2) | 0 |
| Cough | 0 | 0 | 2 (5.6) | 0 | 2 (4.3) | 0 |
| Pleural effusion | 0 | 0 | 1 (2.8) | 0 | 1 (2.2) | 0 |
| hypokalemia | 0 | 0 | 1 (2.8) | 1 (2.8) | 1 (2.2) | 1 (2.2) |
| Hypoalbuminemia | 0 | 0 | 3 (8.3) | 0 | 3 (6.5) | 0 |
| Hypoproteinaemia | 0 | 0 | 1 (2.8) | 0 | 1 (2.2) | 0 |
| Hypocalcaemia | 0 | 0 | 1 (2.8) | 0 | 1 (2.2) | 0 |
| Hypochloraemia | 0 | 0 | 1 (2.8) | 0 | 1 (2.2) | 0 |
| Hypomagnesaemia | 0 | 0 | 1 (2.8) | 0 | 1 (2.2) | 0 |
| Hyponatraemia | 0 | 0 | 3 (8.3) | 0 | 3 (6.5) | 0 |
| Hypoglycemia | 0 | 0 | 1 (2.8) | 0 | 1 (2.2) | 0 |
| Hypertriglyceridemia | 0 | 0 | 2 (5.6) | 0 | 2 (4.3) | 0 |
| Hyperuricemia | 1 (10.0) | 0 | 4 (11.1) | 0 | 5 (10.9) | 0 |
| Hyperglycaemia | 0 | 0 | 4 (11.1) | 0 | 4 (8.7) | 0 |
| Decreased appetite | 0 | 0 | 1 (2.8) | 0 | 1 (2.2) | 0 |
| γ-glutamyltransferase increased | 0 | 0 | 3 (8.3) | 1 (2.8) | 3 (6.5) | 1 (2.2) |
| C-reactive protein increased | 0 | 0 | 1 (2.8) | 0 | 1 (2.2) | 0 |
| Interleukin level increased | 0 | 0 | 1 (2.8) | 0 | 1 (2.2) | 0 |
| White blood cell count decreased | 2 (20.0) | 0 | 5 (13.9) | 0 | 7 (15.2) | 0 |

| Adverse events | Part B (Tifcemalimab in Combination with Toripalimab) JS004 100mg+JS001 240 mg (N=10) n (%) | | JS004 200mg+JS001 240 mg (N=36) n (%) | | Total (N=46) n (%) | |
|---|---|---|---|---|---|---|
| | Grade 1-2 | Grade 3-4 | Grade 1-2 | Grade 3-4 | Grade 1-2 | Grade 3-4 |
| Alanine aminotransferase increased | 1 (10.0) | 0 | 3 (8.3) | 0 | 4 (8.7) | 0 |
| Bile acids increased | 0 | 0 | 2 (5.6) | 0 | 2 (4.3) | 0 |
| Amylase increased | 1 (10.0) | 0 | 1 (2.8) | 0 | 2 (4.3) | 0 |
| Activated partial thromboplastin time prolonged | 0 | 0 | 2 (5.6) | 0 | 2 (4.3) | 0 |
| Activated partial thromboplastin time abnormal | 1 (10.0) | 0 | 4 (11.1) | 0 | 5 (10.9) | 0 |
| Thyroxine decreased | 0 | 0 | 1 (2.8) | 0 | 1 (2.2) | 0 |
| Lymphocyte count decreased | 1 (10.0) | 0 | 1 (2.8) | 0 | 2 (4.3) | 0 |
| Protein urine present | 3 (30.0) | 0 | 1 (2.8) | 0 | 4 (8.7) | 0 |
| Red blood cells urine positive | 1 (10.0) | 0 | 0 | 0 | 1 (2.2) | 0 |
| Urinary occult blood positive | 1 (10.0) | 0 | 1 (2.8) | 0 | 2 (4.3) | 0 |
| Globulins decreased | 0 | 0 | 1 (2.8) | 0 | 1 (2.2) | 0 |
| Weight decreased | 1 (10.0) | 0 | 2 (5.6) | 0 | 3 (6.5) | 0 |
| Weight increased | 1 (10.0) | 0 | 3 (8.3) | 0 | 4 (8.7) | 0 |
| Aspartate aminotransferase increased | 1 (10.0) | 0 | 2 (5.6) | 0 | 3 (6.5) | 0 |
| Fibrin D dimer increased | 0 | 0 | 1 (2.8) | 0 | 1 (2.2) | 0 |
| Electrocardiogram QIT prolonged | 0 | 0 | 2 (5.6) | 0 | 2 (4.3) | 0 |
| Electrocardiogram ST segment elevation | 0 | 0 | 2 (5.6) | 0 | 2 (4.3) | 0 |
| Electrocardiogram T wave abnormal | 0 | 0 | 1 (2.8) | 0 | 1 (2.2) | 0 |
| Blood thyroid stimulating hormone decreased | 1 (10.0) | 0 | 1 (2.8) | 0 | 2 (4.3) | 0 |
| Blood thyroid stimulating hormone increased | 0 | 0 | 5 (13.9) | 0 | 5 (10.9) | 0 |
| Blood bilirubin increased | 1 (10.0) | 0 | 3 (8.3) | 0 | 4 (8.7) | 0 |
| Haemoglobin decreased | 0 | 0 | 1 (2.8) | 0 | 1 (2.2) | 0 |
| Blood creatine phosphokinase increased | 0 | 0 | 1 (2.8) | 0 | 1 (2.2) | 0 |
| Blood creatine increased | 0 | 0 | 2 (5.6) | 0 | 2 (4.3) | 0 |
| Serum ferritin increased | 0 | 0 | 1 (2.8) | 0 | 1 (2.2) | 0 |
| Blood lactate dehydrogenase increased | 0 | 0 | 3 (8.3) | 0 | 3 (6.5) | 0 |
| Blood iron decreased | 0 | 0 | 1 (2.8) | 0 | 1 (2.2) | 0 |
| Blood fibrinogen decreased | 1 (10.0) | 0 | 1 (2.8) | 0 | 2 (4.3) | 0 |
| Blood fibrinogen increased | 0 | 0 | 1 (2.8) | 0 | 1 (2.2) | 0 |
| Platelet count decreased | 1 (10.0) | 0 | 6 (16.7) | 0 | 7 (15.2) | 0 |
| Blood pressure increased | 0 | 0 | 1 (2.8) | 0 | 1 (2.2) | 0 |
| Blood pressed abnormal | 0 | 0 | 1 (2.8) | 0 | 1 (2.2) | 0 |
| Thyroxine free decreased | 0 | 0 | 1 (2.8) | 0 | 1 (2.2) | 0 |
| Triiodothyronine free increased | 0 | 0 | 1 (2.8) | 0 | 1 (2.2) | 0 |
| Lipase increased | 2 (20.0) | 0 | 2 (5.6) | 0 | 4 (8.7) | 0 |
| Neutrophil count decreased | 0 | 0 | 5 (13.9) | 0 | 5 (10.9) | 0 |
| Tumor marker increased | 0 | 0 | 1 (2.8) | 0 | 1 (2.2) | 0 |
| Papillary thyroid cancer | 0 | 0 | 0 | 1 (2.8) | 0 | 1 (2.2) |
| Anaphylactic shock | 0 | 0 | 0 | 1 (2.8) | 0 | 1 (2.2) |
| Hypersensitivity | 0 | 0 | 2 (5.6) | 0 | 2 (4.3) | 0 |

| Adverse events | Part B (Tifcemalimab in Combination with Toripalimab) | | | | | |
|---|---|---|---|---|---|---|
| | JS004 100mg+JS001 240 mg (N=10) n (%) | | JS004 200mg+JS001 240 mg (N=36) n (%) | | Total (N=46) n (%) | |
| | Grade 1-2 | Grade 3-4 | Grade 1-2 | Grade 3-4 | Grade 1-2 | Grade 3-4 |
| Drug eruption | 0 | 0 | 0 | 1 (2.8) | 0 | 1 (2.2) |
| Rash maculopapular | 0 | 0 | 2 (5.6) | 0 | 2 (4.3) | 0 |
| Lichen planus | 1 (10.0) | 0 | 0 | 0 | 1 (2.2) | 0 |
| Night sweats | 1 (10.0) | 0 | 0 | 0 | 1 (2.2) | 0 |
| hyperhidrosis | 0 | 0 | 1 (2.8) | 0 | 1 (2.2) | 0 |
| Dry skin | 0 | 0 | 3 (8.3) | 0 | 3 (6.5) | 0 |
| Rash | 0 | 0 | 6 (16.7) | 0 | 6 (13.0) | 0 |
| Papule | 1 (10.0) | 0 | 0 | 0 | 1 (2.2) | 0 |
| Psoriasis | 0 | 0 | 1 (2.8) | 0 | 1 (2.2) | 0 |
| Seborrhoeic dermatitis | 0 | 0 | 1 (2.8) | 0 | 1 (2.2) | 0 |
| Urticaria | 0 | 0 | 2 (5.6) | 0 | 2 (4.3) | 0 |
| Pruritus | 2 (20.0) | 0 | 2 (5.6) | 0 | 4 (8.7) | 0 |
| Anaemia | 3 (30.0) | 0 | 7 (19.4) | 1 (2.8) | 10 (21.7) | 1 (2.2) |
| Headache | 0 | 0 | 1 (2.8) | 0 | 1 (2.2) | 0 |
| Dizziness | 0 | 0 | 1 (2.8) | 0 | 1 (2.2) | 0 |
| Epilepsy | 0 | 0 | 1 (2.8) | 0 | 1 (2.2) | 0 |
| Infusion related reaction | 0 | 0 | 1 (2.8) | 0 | 1 (2.2) | 0 |
| Back pain | 0 | 0 | 3 (8.3) | 0 | 3 (6.5) | 0 |
| Bone pain | 1 (10.0) | 0 | 1 (2.8) | 0 | 2 (4.3) | 0 |
| Arthralgia | 2 (20.0) | 0 | 1 (2.8) | 0 | 3 (6.5) | 0 |
| Myalgia | 0 | 0 | 1 (2.8) | 0 | 1 (2.2) | 0 |
| Spinal pain | 1 (10.0) | 0 | 0 | 0 | 1 (2.2) | 0 |
| Neck pain | 0 | 0 | 1 (2.8) | 0 | 1 (2.2) | 0 |
| Pain in extremity | 0 | 0 | 1 (2.8) | 0 | 1 (2.2) | 0 |
| Insomnia | 0 | 0 | 1 (2.8) | 0 | 1 (2.2) | 0 |
| Hypothyroidism | 2 (20.0) | 0 | 5 (13.9) | 0 | 7 (15.2) | 0 |
| Hyperthyroidism | 2 (20.0) | 0 | 1 (2.8) | 0 | 3 (6.5) | 0 |
| Thyroid disorder | 0 | 0 | 1 (2.8) | 0 | 1 (2.2) | 0 |
| Thyroid mass | 0 | 0 | 1 (2.8) | 0 | 1 (2.2) | 0 |
| Proteinuria | 0 | 0 | 1 (2.8) | 0 | 1 (2.2) | 0 |
| Dysmenorrhea | 0 | 0 | 1 (2.8) | 0 | 1 (2.2) | 0 |
| Constipation | 0 | 0 | 1 (2.8) | 0 | 1 (2.2) | 0 |
| Nausea | 0 | 0 | 1 (2.8) | 0 | 1 (2.2) | 0 |
| Abdominal pain | 0 | 0 | 4 (11.1) | 0 | 4 (8.7) | 0 |
| Diarrhoea | 0 | 0 | 2 (5.6) | 0 | 2 (4.3) | 0 |
| Abdominal distension | 1 (10.0) | 0 | 2 (5.6) | 0 | 3 (6.5) | 0 |
| Dry mouth | 0 | 0 | 1 (2.8) | 0 | 1 (2.2) | 0 |
| Vomiting | 0 | 0 | 1 (2.8) | 0 | 1 (2.2) | 0 |
| Duodenal ulcer | 0 | 0 | 1 (2.8) | 0 | 1 (2.2) | 0 |
| Gastritis | 0 | 0 | 1 (2.8) | 0 | 1 (2.2) | 0 |
| Toothache | 0 | 0 | 1 (2.8) | 0 | 1 (2.2) | 0 |
| Gingival bleeding | 1 (10.0) | 0 | 0 | 0 | 1 (2.2) | 0 |
| Atrial tachycardia | 0 | 0 | 1 (2.8) | 0 | 1 (2.2) | 0 |
| Supraventricular extrasystoles | 0 | 0 | 1 (2.8) | 0 | 1 (2.2) | 0 |
| Bradycardia | 0 | 0 | 1 (2.8) | 0 | 1 (2.2) | 0 |
| Tachycardia | 0 | 0 | 2 (5.6) | 0 | 2 (4.3) | 0 |
| Palpitations | 0 | 0 | 1 (2.8) | 0 | 1 (2.2) | 0 |
| Arrhythmia | 0 | 0 | 1 (2.8) | 0 | 1 (2.2) | 0 |
| Atrioventricular block first degree | 0 | 0 | 1 (2.8) | 0 | 1 (2.2) | 0 |
| Bundle branch block right | 1 (10.0) | 0 | 0 | 0 | 1 (2.2) | 0 |

| Adverse events | Part B (Tifcemalimab in Combination with Toripalimab) | | | | | |
|---|---|---|---|---|---|---|
| | JS004 100mg+JS001 240 mg (N=10) n (%) | | JS004 200mg+JS001 240 mg (N=36) n (%) | | Total (N=46) n (%) | |
| | Grade 1-2 | Grade 3-4 | Grade 1-2 | Grade 3-4 | Grade 1-2 | Grade 3-4 |
| Bundle branch block left | 0 | 0 | 1 (2.8) | 0 | 1 (2.2) | 0 |
| Sinus bradycardia | 1 (10.0) | 0 | 7 (19.4) | 0 | 8 (17.4) | 0 |
| Sinus tachycardia | 0 | 0 | 7 (19.4) | 0 | 7 (15.2) | 0 |
| Sinus arrhythmia | 0 | 0 | 2 (5.6) | 0 | 2 (4.3) | 0 |
| Hypotension | 1 (10.0) | 0 | 5 (13.9) | 0 | 6 (13.0) | 0 |

## Supplementary Table 5: Treatment-Related Adverse Events Leading to Treatment Discontinuation (Part B)

| | Part B (Tifcemalimab in Combination with Toripalimab) | | |
|---|---|---|---|
| **Adverse events** | **JS004 100mg+JS001 240 mg (N=10) n (%)** | **JS004 200mg+JS001 240 mg (N=36) n (%)** | **Total (N=46) n (%)** |
| Any TRAE | 1 (10.0) | 13 (36.1) | 14 (30.4) |
| Pneumonia | 0 | 2 (5.6) | 2 (4.3) |
| Abdominal pain | 0 | 2 (5.6) | 2 (4.3) |
| Infection | 0 | 1 (2.8) | 1 (2.2) |
| Sepsis | 0 | 1 (2.8) | 1 (2.2) |
| Pneumonia bacterial | 0 | 1 (2.8) | 1 (2.2) |
| Pneumocystis jirovecii pneumonia | 0 | 1 (2.8) | 1 (2.2) |
| Bronchitis | 0 | 1 (2.8) | 1 (2.2) |
| Pneumonitis | 0 | 1 (2.8) | 1 (2.2) |
| Interstitial lung disease | 0 | 1 (2.8) | 1 (2.2) |
| Pleurisy | 0 | 1 (2.8) | 1 (2.2) |
| Pleural effusion | 0 | 1 (2.8) | 1 (2.2) |
| Amylase increased | 0 | 1 (2.8) | 1 (2.2) |
| Platelet count decreased | 0 | 1 (2.8) | 1 (2.2) |
| Lipase increased | 0 | 1 (2.8) | 1 (2.2) |
| Facial pain | 0 | 1 (2.8) | 1 (2.2) |
| Oedema peripheral | 0 | 1 (2.8) | 1 (2.2) |
| Duodenal ulcer | 0 | 1 (2.8) | 1 (2.2) |
| Hypokalemia | 0 | 1 (2.8) | 1 (2.2) |
| Immune-mediated hepatitis | 0 | 1 (2.8) | 1 (2.2) |
| Infusion related reaction | 0 | 1 (2.8) | 1 (2.2) |
| Back pain | 0 | 1 (2.8) | 1 (2.2) |
| Pain in extremity | 0 | 1 (2.8) | 1 (2.2) |
| Hypersensitivity | 0 | 1 (2.8) | 1 (2.2) |
| Hypothyroidism | 1 (10.0) | 0 | 1 (2.2) |
| Drug eruption | 0 | 1 (2.8) | 1 (2.2) |
| Anaemia | 0 | 1 (2.8) | 1 (2.2) |

## Supplementary Table 6: Receptor Occupancy (%) of T and B cells [Mean±SD (N)] in Part A

| Timepoint | | C1 (pre-dose) | C1 (24h) | C1 (168h) | C2 (pre-dose) | C4 (pre-dose) | C6 (pre-dose) | C8 (pre-dose) | EOT |
|---|---|---|---|---|---|---|---|---|---|
| 1mg/kg | CD3+T cell (%) | 0 (3) | 90·1±7·1 (3) | 93·8±0·3 (3) | 91·7±2·1 (3) | 88·5±2·5 (3) | 91·5±1·9 (3) | 98·7 (1) | 90·8±0·7 (3) |
| 3mg/kg | | 0 (12) | 96·7±2·6 (12) | 99·9±10·8 (12) | 94·7±2 (10) | 92·9±3·2 (5) | 92·9±2·3 (3) | 91·8±2 (3) | 87·8±9·9 (7) |
| 10mg/kg | | 0 (3) | 97·8±3·1 (3) | 98·2±1·7 (2) | 91·3±2·1 (2) | NA | NA | NA | 94±6·4 (3) |
| 200mg | | 0 (7) | 97·1±3·3 (7) | 96·9±2·5 (7) | 87·5±10·9 (7) | 91·4±2·4 (6) | 99·1±17·9 (4) | 92·8±1·3 (2) | 91·2±2·6 (5) |
| 1mg/kg | CD20+B cell (%) | 0 (3) | 87·3±6·2 (3) | 89±0·9 (3) | 86·5±2·1 (3) | 85·6±2·3 (3) | 85·8±1·4 (3) | 89 (1) | 86·7±3·3 (3) |
| 3mg/kg | | 0 (12) | 92·6±3·3 (12) | 91·9±2·1 (12) | 89·2±1·6 (11) | 88·7±5·9 (5) | 84·8±1·7 (3) | 86·6±0·7 (3) | 87·7±4·9 (6) |
| 10mg/kg | | 0 (3) | 97·9±2·7 (3) | 96·5±4·2 (2) | 88·5±3·3 (2) | NA | NA | NA | 90·2±5·3 (3) |
| 200mg | | 0 (7) | 94±4·6 (7) | 89·7±1·7 (7) | 87·6±4 (7) | 86·3±2·1 (6) | 87·4±8·7 (4) | 87·5±4·6 (2) | 85·4±3·1 (5) |

**Supplementary Table 7: Summary of PK Parameters of Tifcemalimab Monotherapy and Tifcemalimab in Combination with Toripalimab Following Single Dose and Multiple Doses in Patients with Relapsed/Refractory Lymphoma**

| | Cycle 1 | | | | | Cycle 4 | | | | | |
|---|---|---|---|---|---|---|---|---|---|---|---|
| **Dose level** | **N** | **$C_{max}$ (μg/mL)** | **$T_{1/2}$ (day)** | **$AUC_{0-\infty}$ (h*μg/mL)** | **$AUC_{Tau}$ * (h*μg/mL)** | **N** | **$C_{max}$ (μg/mL)** | **$C_{trough}$(μg/mL)** | **$T_{1/2}$ (day)** | **$AUC_{Tau}$ * (h*μg/mL)** | **Rac** |
| **Part A(tifcemalimab)** | | | | | | | | | | | |
| 1 mg/kg every 3 weeks | 3 | 23·8 ±6·75 | 11·8 ±2·36 | 5789·7 ± 1912·04 | 4021·4 ± 1058·26 | 3 | 31·4 ±7·08 | 12·9 ±3·21 | 17·3 ±18·5 | 6501·1 ± 1617·99 | 1·8 ±1·19 |
| 3 mg/kg every 3 weeks | 12 | 60·6 ±19·2 | 13·1 ±4·96 | 18476·0 ± 7385·26 | 11923·4 ± 3531·51 | 6 | 81·8 ±28·3 | 34·7 ±14·1 | 12·2 ±4·08 | 17483·0 ± 7119·49 | 1·4 ±0·25 |
| 10 mg/kg every 3 weeks | 3 | 193 ±59·1 | 17·6 ±1·05 | 81731·9 ±25539·43 | 46553·9 ±14172·82 | | ND | ND | ND | ND | ND |
| 200 mg every 3 weeks | 7 | 67·6 ±10·8 | 10·5 ±2·08 | 16699·6 ± 3256·18 | 12542·2 ± 2037·11 | 6 | 86·4 ±13·7 | 45·0 ±10·6 | 16·5 ±4·86 | 22681.3 ± 5358.03 | 1·7 ±0·32 |
| **Part B (tifcemalimab + toripalimab)** | | | | | | | | | | | |
| 100mg+240mg every 3 weeks | 10 | 33·5 ±8·07 | 10·3 ±1·86 | 9202·8 ± 2659·58 | 6877·1 ± 1666·78 | 7 | 41·2 ±7·47 | 20·4 ±6·99 | 11·4 ±3·03 | 10277·0 ± 3523·55 | 1·4 ±0·18 |
| 200mg+240mg every 3 weeks | 10 | 49·0 ±8·77 | 17·2 ±7·73 | 20047·7 ±10422·56 | 10669·4 ± 3754·31 | 10 | 71·6 ±18·5 | 36·9 ±14·5 | 14·8 ±5·85 | 18620·2 ± 7288·23 | 1·6 ±0·36 |

*Tau was defined as 21 days for every 3 weeks.

$AUC_{0-\infty}$, area under the curve from 0 to infinity; $T_{1/2}$, elimination half-life; Rac, accumulation ratio; $C_{max}$, peak concentration; $C_{trough}$, valley concentration; ND, no data.

**Supplementary Table 8: The Ratio of Cytokines Concentration 24 Hours Post-Administration Compared to Pre-Dose Levels**

| Groups | Doses | N | IFN-γ | IL-6 | IL-8 | IL-10 | TNF-α |
|---|---|---|---|---|---|---|---|
| Part A | 1 mg/kg | 3 | 1.59±0.37 | 2.19±0.85 | 1.51±0.39 | 1.22±0.2 | 1.12±0.22 |
| | 3 mg/kg | 12 | 1.82±1.46 | 2.43±3.99 | 1.36±0.51 | 1.13±0.16 | 1.08±0.16 |
| | 10 mg/kg | 3 | 1.17±0.12 | 5.56±5.64 | 2.25±1.22 | 1.13±0.21 | 1.13±0.28 |
| | 200 mg | 6 | 1.43±0.41 | 1.11±0.28 | 1.12±0.48 | 1.19±0.25 | 1.09±0.06 |
| Part B | 100 mg | 4 | 2.17±1.51 | 2.71±2.08 | 3.04±0.87 | 3.35±3.63 | 1.36±0.1 |
| | 200 mg | 3 | 6.21±5.15 | 5.29±3.56 | 1.35±0.51 | 15.96±16.67 | 1.74±0.37 |

The table presented the ratio of cytokines concentration 24 hours post-administration compared to pre-dose levels for each dose group. The results indicated varying degrees of cytokine elevation appeared 24 hours after initial administration in Part A and Part B groups, with a slightly higher ratio observed in the Part B group compared to the Part A group, this observation should be further confirmed due to limited sample size.

**Supplementary Table 9: Summary of Tumor Response for Monotherapy, Combination Therapy and Classical Hodgkin's Lymphoma (Full Analysis Set)**

| | **Best Overall Response** | | | | | **Overall Response rate (%)** | **Disease Control Rate (%)** | **Duration of Response (months)** |
|---|---|---|---|---|---|---|---|---|
| | **Complete response** | **Partial Response** | **Stable disease** | **Progressive disease** | **Not evaluable** | | | |
| Monotherapy (Part A) (N=25) | 0 | 1 | 6 | 17 | 1 | 4.0 | 28.0 | 4.1 |
| Combination therapy (Pat B) (N=46) | 1 | 16 | 20 | 6 | 3 | 37.0 | 80.4 | 12.5 |
| cHL patients who had received PD-(L)1 blockade (Part A 3 mg/kg and 200 mg cohort) (N= 7) | 0 | 0 | 3 | 4 | 0 | 0 | 42.9 | NA |
| cHL patients who had received PD-(L)1 blockade (Part B RP2D cohort) (N= 34) | 0 | 12 | 17 | 4 | 1 | 35.3 | 85.3 | 6.3 |
| cHL patients refractory to PD-(L)1 blockade (Part B RP2D cohort) [1] (N=29) | 0 | 9 | 15 | 4 | 1 | 31.0 | 82.8 | 8.4 |
| Relapsed cHL after PD-(L)1 blockade (Part B RP2D cohort) (N=5) [2] | 0 | 3 | 2 | 0 | 0 | 60.0 | 100.0 | 6.1 |

[1] PD-1 refractory was defined as disease progress within 3 months from the last dose of PD-1.
[2] Relapse was defined as progression after 3 months from the last dose of PD-1.

**Supplementary Table 10 Herpes Virus Entry Mediator (HVEM) and Programmed Cell Death Ligand 1 (PD-L1) Staining**

| Histology | Patient ID | HVEM lymphoma score | PD-L1 TPS |
|---|---|---|---|
| Hodgkin lymphoma | 01017 | 60 | NA |
| Hodgkin lymphoma | 01018 | 60 | ≥50% |
| Hodgkin lymphoma | 01019 | 80 | <1% |
| Hodgkin lymphoma | 01020 | 20 | 1-5% |
| Hodgkin lymphoma | 01021 | 70 | NA |
| Hodgkin lymphoma | 01023 | 20 | NA |
| Hodgkin lymphoma | 01024 | 10 | ≥50% |
| Hodgkin lymphoma | 09004 | 25 | ≥50% |
| Hodgkin lymphoma | 14002 | 5 | NA |
| Hodgkin lymphoma | 14004 | 0 | 26%-49% |
| Hodgkin lymphoma | 01027 | 95 | NA |
| Hodgkin lymphoma | 02008 | 5 | <1% |
| Hodgkin lymphoma | 03011 | <1 | 1%-5% |
| Hodgkin lymphoma | 05002 | NA | ≥50% |
| Hodgkin lymphoma | 06002 | 85 | 11%-25% |
| Hodgkin lymphoma | 08001 | 70 | 6%-10% |
| Hodgkin lymphoma | 08002 | 75 | 11%-25% |
| Hodgkin lymphoma | 08003 | 80 | 26%-49% |
| Hodgkin lymphoma | 09005 | 55 | ≥50% |
| Hodgkin lymphoma | 09007 | 20 | <1% |
| Hodgkin lymphoma | 10002 | NA | 6%-10% |
| Hodgkin lymphoma | 01011 | 100 | 6%-10% |
| Peripheral T-cell lymphoma | 09003 | 20 | NA |
| Follicular lymphoma | 03009 | 3 | <1% |
| Follicular lymphoma | 02006 | NA | <1% |
| Peripheral T-cell lymphoma | 10001 | NA | <1% |
| Hodgkin lymphoma | 01001 | NA | 26%-49% |
| Follicular lymphoma | 01008 | NA | 11%-25% |
| Diffuse large B-cell lymphoma | 01009 | NA | <1% |
| Follicular lymphoma | 01010 | NA | <1% |
| Hodgkin lymphoma | 01013 | NA | ≥50% |
| Diffuse large B-cell lymphoma | 02001 | NA | 26%-49% |
| Diffuse large B-cell lymphoma | 02002 | NA | ≥50% |
| Diffuse large B-cell lymphoma | 02003 | NA | 1%-5% |
| Peripheral T-cell lymphoma | 02005 | NA | <1% |
| Follicular lymphoma | 03001 | NA | <1% |
| Hodgkin lymphoma | 03003 | NA | 11%-25% |
| Follicular lymphoma | 03004 | NA | <1% |
| Follicular lymphoma | 03005 | NA | <1% |
| Follicular lymphoma | 03010 | NA | 1%-5% |
| Diffuse large B-cell lymphoma | 04002 | NA | <1% |

HVEM = herpes virus entry mediator, PD-L1 = programmed cell death ligand 1, TPS = tumor proportion score, NA = not available.

**Supplementary Figure 1: Serum Concentration of Tifcemalimab after First Dosing**

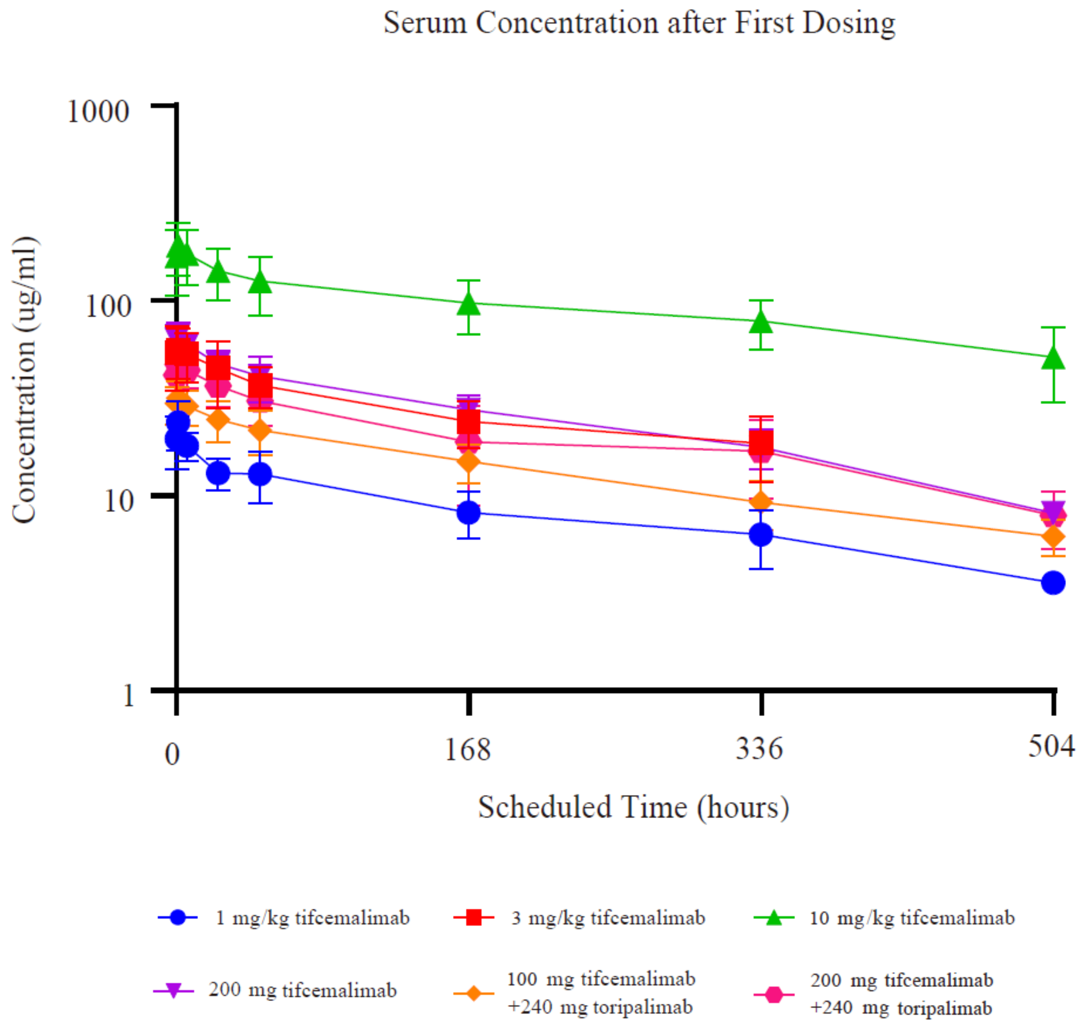


**Figure Legend:** The figure shows the serum concentration of tifcemalimab after the first dose of tifcemalimab 1 mg/kg (N=3), 3 mg/kg (N=12), 10 mg/kg (N=3), 200 mg (N=7), and the tifcemalimab combination therapy (10 patients in the 100 mg cohort and 10 in the 200 mg cohort). 'N' indicates the number of subjects included in the statistics; data are presented as mean values +/- SD. Source data are provided as a Source Data file.

## Supplementary Figure 2: Progression-Free Survival Status

A)

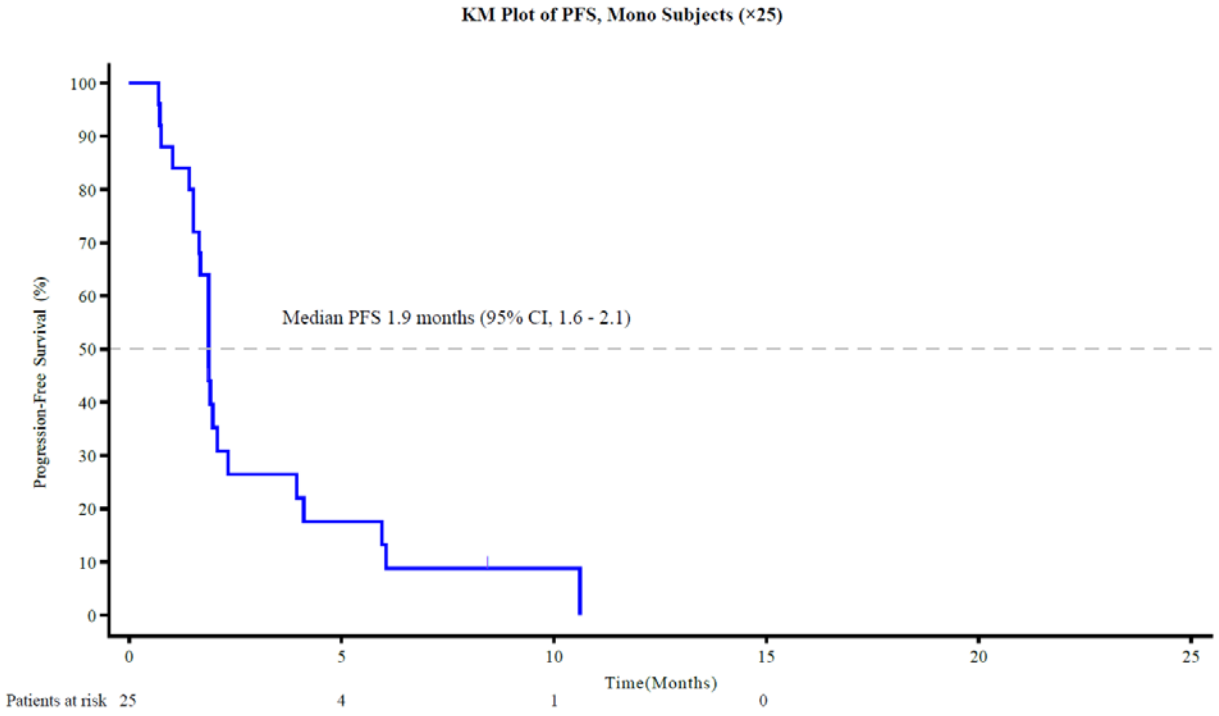


B)

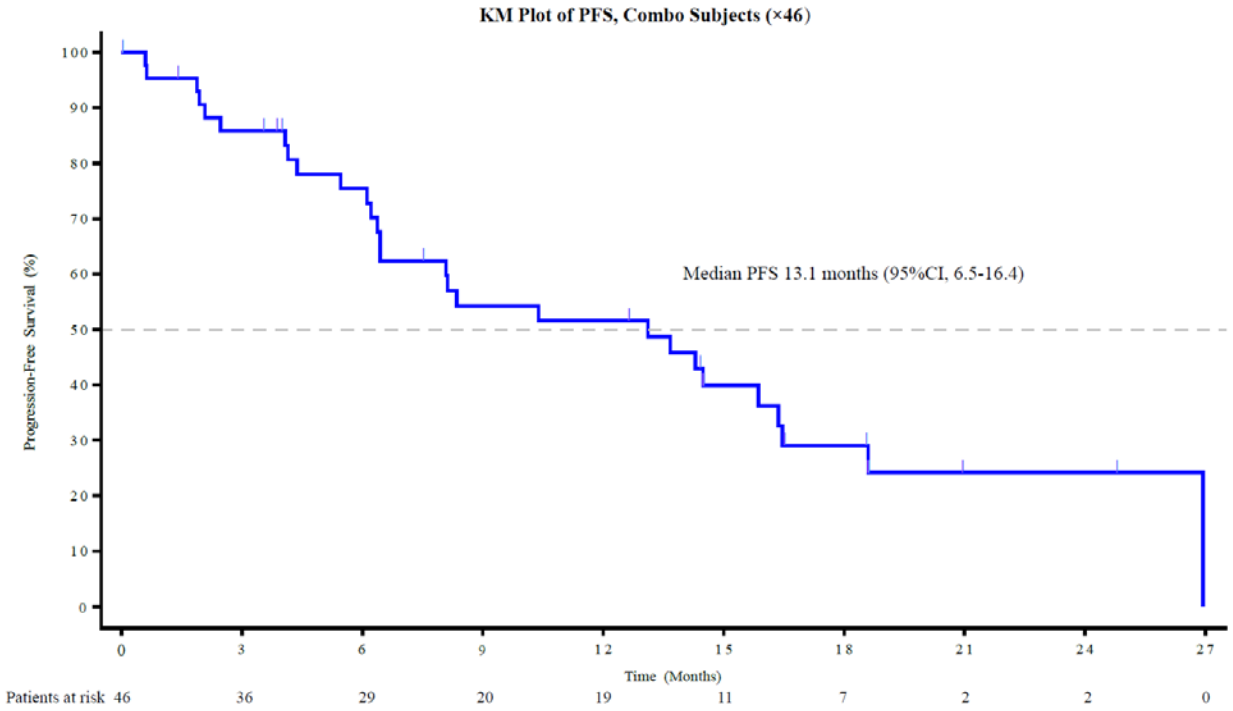

C)

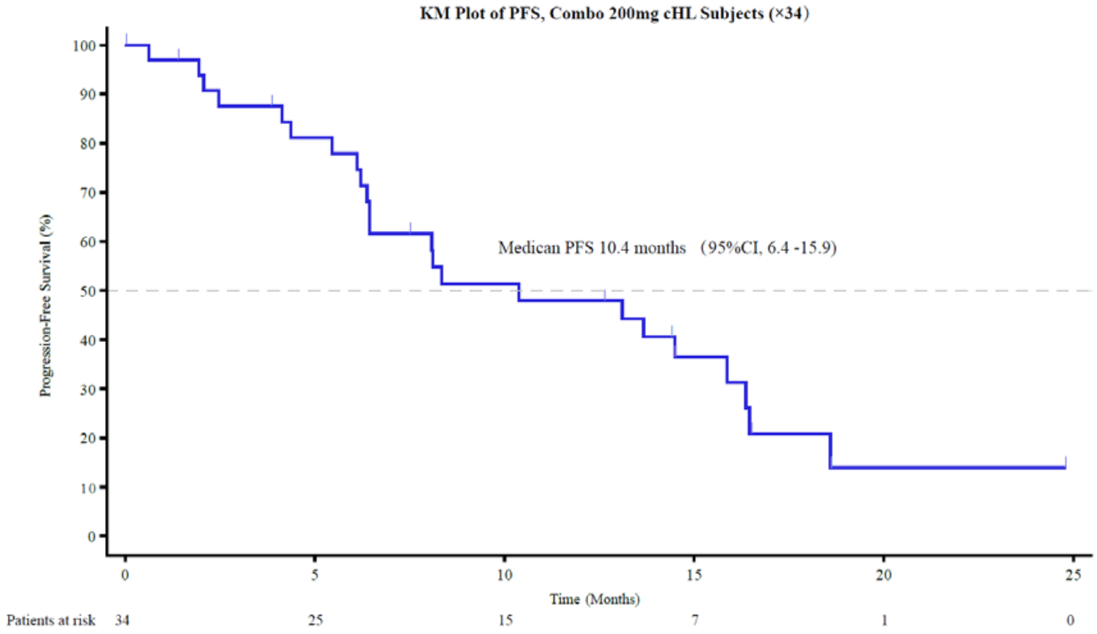


D)

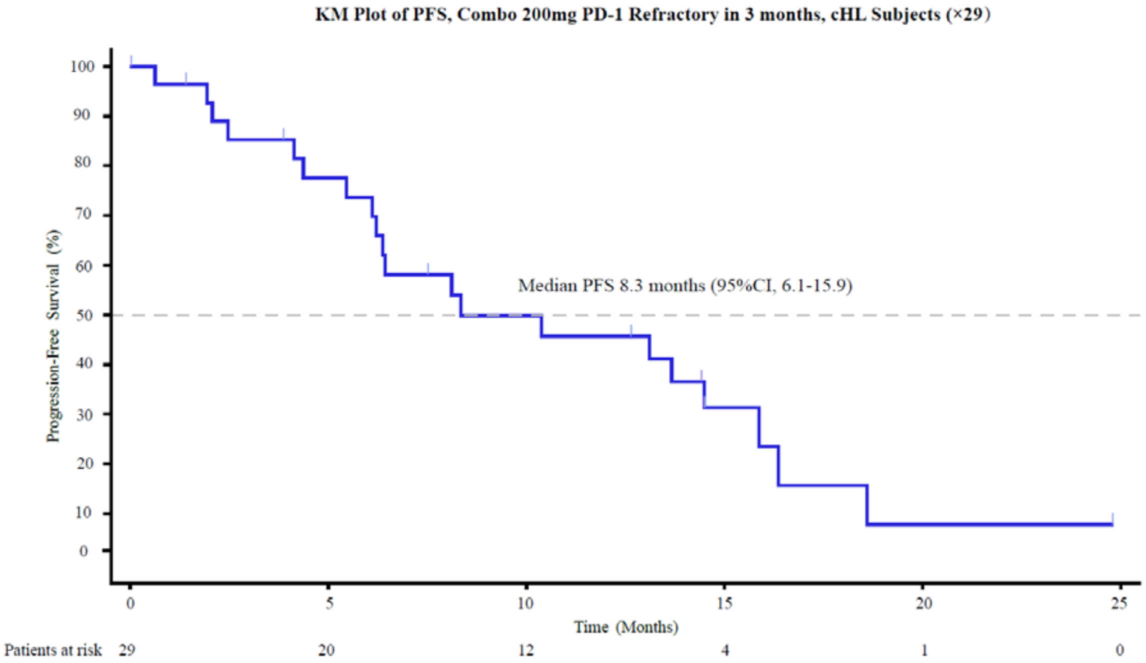


**Figure legend:** Figure A to D shows the Kaplan-Meier curve for progressive-free survival among patients with monotherapy (25 patients), combination therapy (46 patients), patients with classical Hodgkin's lymphoma (cHL) who had previously received PD-(L)1 blockade (N=34), and cHL patients who had progressed on PD-(L)1 blockade within 3 months (29 patients). cHL = classical Hodgkin's lymphoma, DLBCL = diffuse large B-cell lymphoma, FL = follicular lymphoma, KM = Kaplan-Meier, PD-1 = programmed cell death 1, PFS = progression-free survival, PTCL = peripheral T-cell lymphoma.

Kaplan-Meier method was applied in estimating the median of PFS, and associates 95% CI was estimated with Brookmeyer-Crowley method. Source data are provided as a Source Data file.

## Supplementary Figure 3 Tumor response

A)

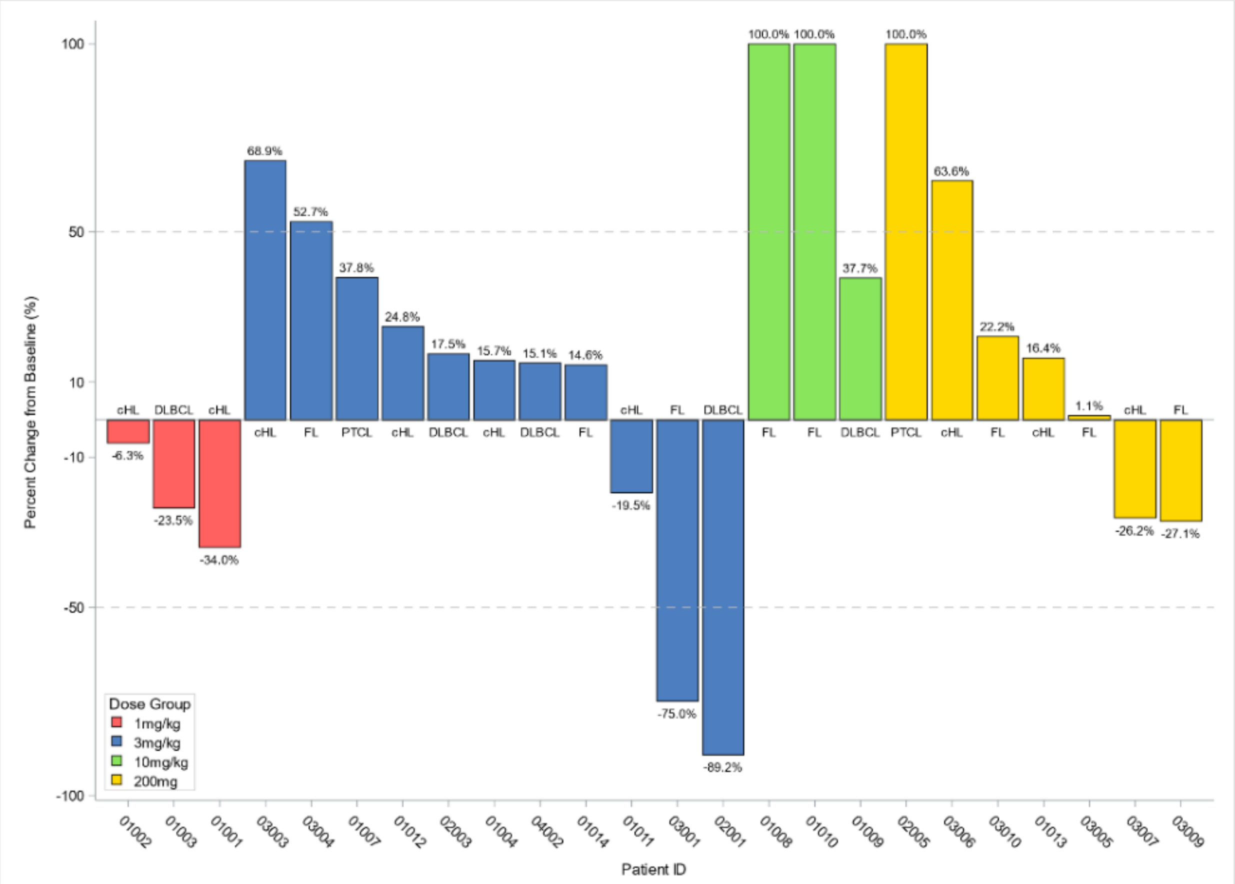


cHL=classical Hodgkin's lymphoma, DLBCL=diffuse large B-cell lymphoma, FL=follicular lymphoma, PTCL=peripheral T-cell lymphoma.

B)

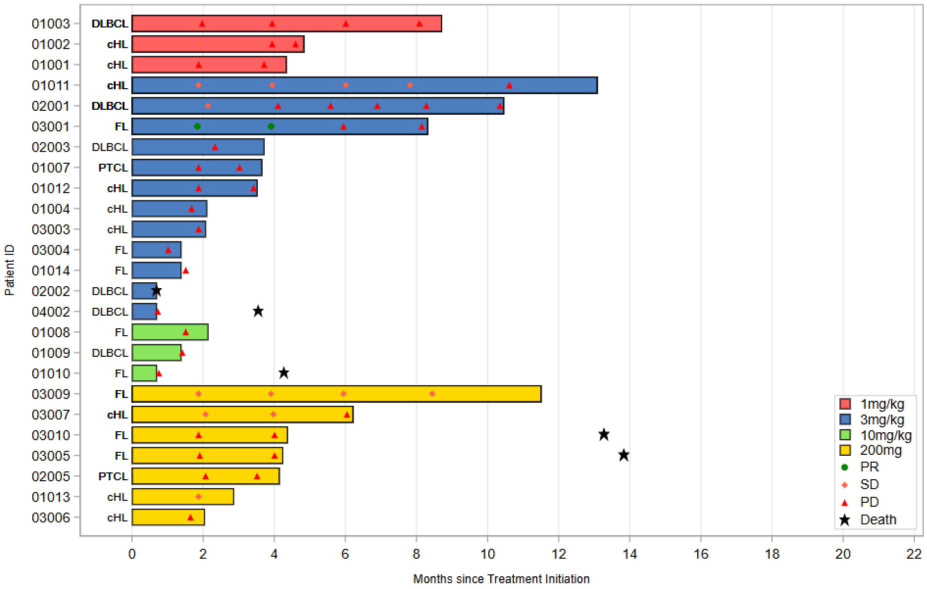


cHL=classical Hodgkin's lymphoma, DLBCL=diffuse large B-cell lymphoma, FL=follicular lymphoma, PTCL=peripheral T-cell lymphoma.

C)

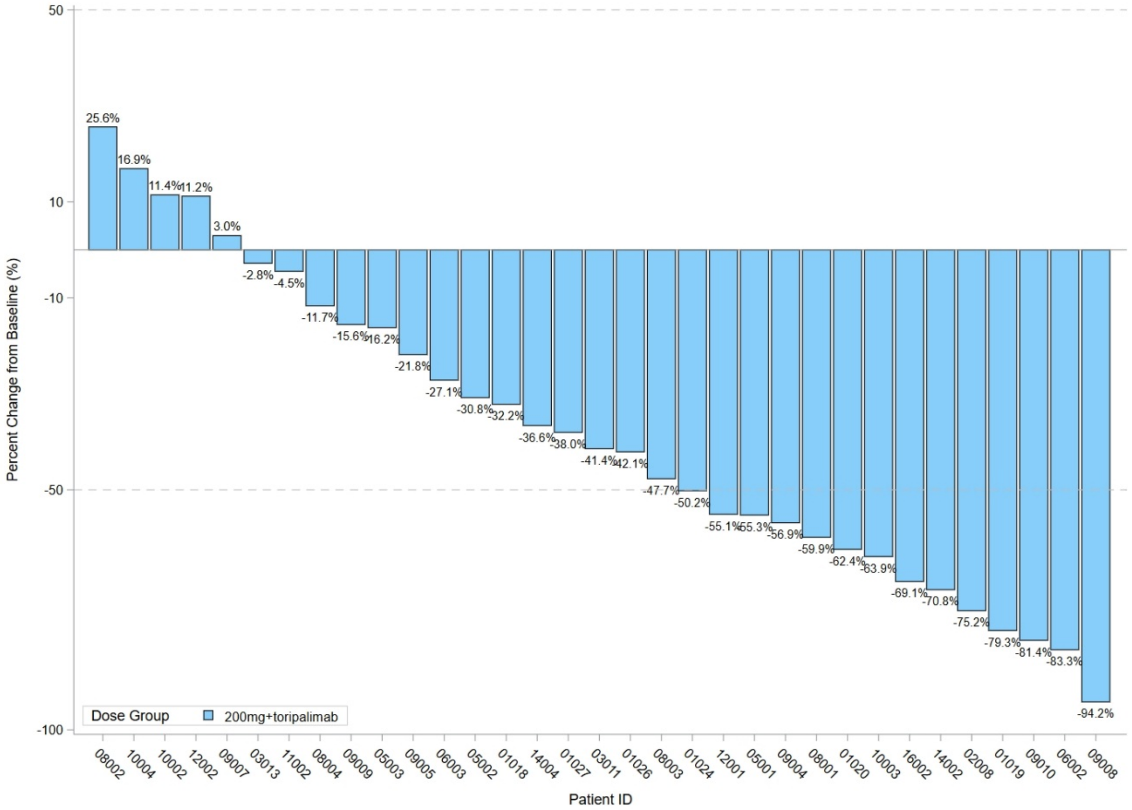


D)

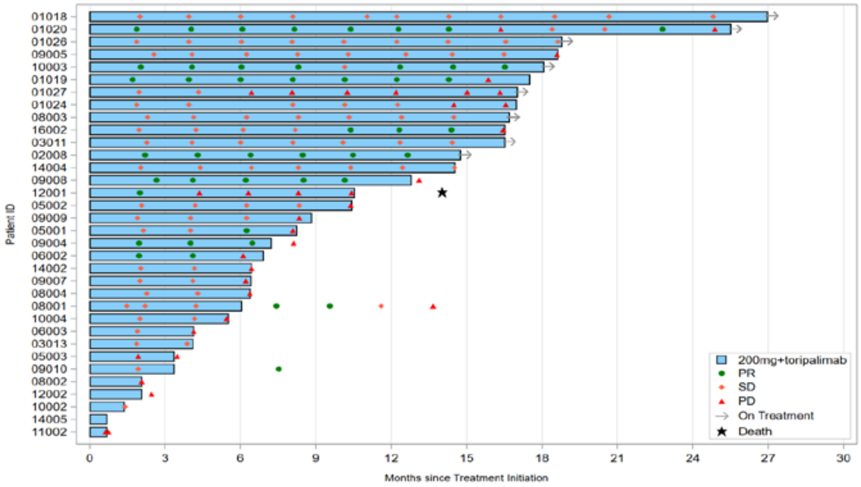


**Figure legend**: Figure A shows the best change of target after administration of tifcemalimab 1 mg/kg, 3 mg/kg, 10 mg/kg and 200 mg cohort (24 patients*). Figure B shows the tumor response after administration of tifcemalimab 1 mg/kg, 3 mg/kg, 10 mg/kg and 200 mg cohort (25 patients). Figure C shows waterfall plot for patients with classical Hodgkin's lymphoma (cHL) who had previously received with PD-(L)1 blockade (34 patients). Figure D shows the tumor response overtime among patients with cHL who had previously received PD-(L)1 blockade (34 patients). Patient 11002 experienced two times of disease progression, and the tumor imaging evaluations were performed in a short interval. * In Figure A, 1 patient in Part A was not included in the figures due to lack of post-baseline tumor evaluation data. cHL = classical Hodgkin's lymphoma, DLBCL = diffuse large B-cell lymphoma, FL = follicular lymphoma, KM = Kaplan-Meier, PD-(L)1 = programmed cell death 1/programmed cell death ligand 1, PFS = progression-free survival, PTCL = peripheral T-cell lymphoma. Source data are provided as a Source Data file.

**Supplementary Figure 4: Representative Immunohistochemistry Staining of HVEM in Patients with Lymphoma**

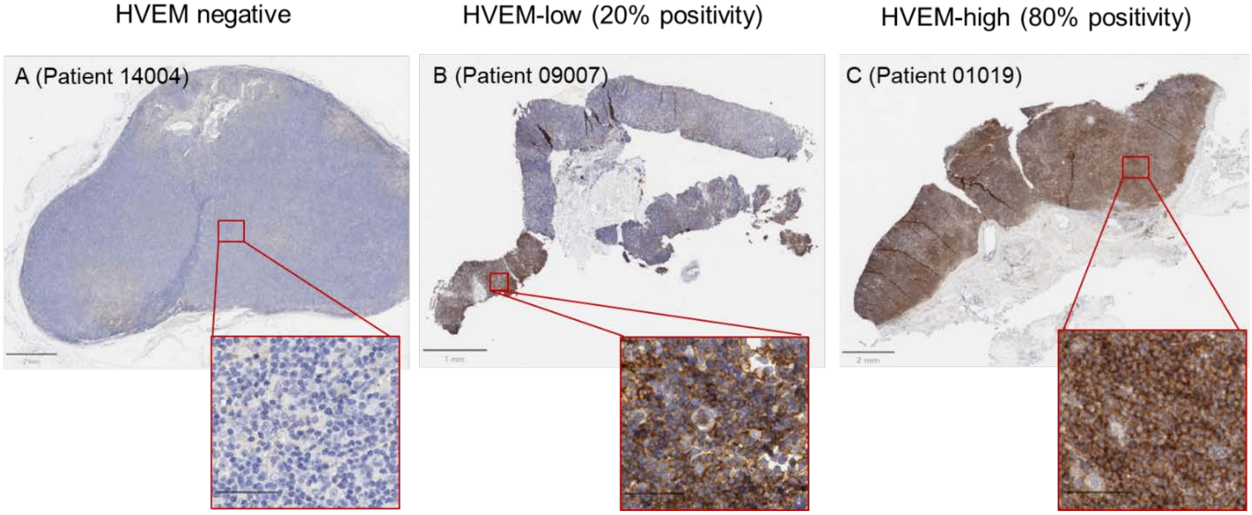


**Figure legend**: HVEM results were reported as the lymphoma score (the positively staining cells over all the nucleated cells within tumor area). The figures shown are the immunohistochemistry staining of tumor samples obtained from patients with a score of 0% (Panel A), a score of 20% (Panel B), and a score of 80% (Panel C). Source data are provided as a Source Data file. Scale bar: 1 or 2mm in whole slide image, 50um in representative area.

# Supplementary Note 1

**List of Ethics Committees**

| No. | Ethics Committees |
|---|---|
| 1 | Peking University Cancer Hospital & Institute |
| 2 | Harbin Institute of Hematology & Oncology |
| 3 | Tianjin Medical University Cancer Institute and Hospital |
| 4 | The First Affiliated Hospital of Guangxi Medical University |
| 5 | Jiangxi Cancer Hospital |
| 6 | The First Affiliated Hospital of Nanchang University |
| 7 | Henan Cancer Hospital |
| 8 | Zhejiang Cancer Hospital |
| 9 | West China Hospital of Sichuan University |
| 10 | The Second Hospital of Dalian Medical University |
| 11 | Shanghai Ruijin Hospital |
| 12 | The Fifth Medical Center of the General Hospital of the Chinese People's Liberation Army |
| 13 | The Affiliated Cancer Hospital of Guizhou Medical University |
| 14 | Hunan Cancer Hospital |

# Supplementary Note 2

# Phase I Study of Recombinant Humanized Anti-BTLA Monoclonal Antibody (JS004) in Patients with Relapsed/Refractory Malignant Lymphoma

| | |
|---|---|
| Protocol No.: | JS004-002-I |
| Study Phase: | Phase I |
| Compound No.: | JS004 |
| Compound Name: | Recombinant Humanized Anti-BTLA Monoclonal Antibody |
| Study Leading Unit: | Beijing Cancer Hospital |
| | Harbin Institute of Hematological Oncology |
| Principal Investigators: | Jun Zhu, Jun Ma |
| Version No.: | 2.1 |
| Version Date: | 15-Feb-2022 |
| **Sponsor:** | Shanghai Junshi Biosciences Co., Ltd. |

## Version/Revision History

| **Document** | **Version date** | **Reason for modifications and summary of amendments** |
|---|---|---|
| 1.0 | 11-Mar-2020 | Not applicable |
| 1.1 | 19-Mar-2020 | 1. The starting dose is modified to 0.3 mg/kg.<br>2. Intensive PK blood sampling was performed in Cycle 4, and PK blood sampling was canceled in Cycle 3. |
| 2.0 | 30-Jun-2021 | See Annex of Amendment Description for details |
| 2.1 | 15-Feb-2022 | See Annex of Amendment Description for details |

## Sponsor Protocol Signature Page

We have read and confirmed this protocol (Protocol No.: JS004-002-I, Version No.: 2.1, Version date: 15-Feb-2022). We agree to perform the relevant duties in accordance with the laws of China, the Declaration of Helsinki, Good Clinical Practice (GCP), and this study protocol.

**Sponsor: Shanghai Junshi Biosciences Co., Ltd.**

| Feiwu Ran, Medical Director | | |
|---|---|---|
| (print) | (Signature) | Date of signature (DD-MM-YYYY) |

## Principal Investigator Protocol Signature Page

I will carefully perform the investigator's duties per the provisions under Good Clinical Practice (hereinafter referred to as GCP). I will provide a copy of the protocol to my study team and discuss it to ensure that they fully understand this study. In addition, I will personally participate in or directly guide the study. I have received the Investigator's Brochure for the study drug in this study, and I have known and read the preclinical study of the study drug and the protocol of the study (Protocol No.: JS004-002-I, Version No.: 2.1, Version date: 15-Feb-2022). I agree to perform the relevant duties in strict accordance with China's relevant laws and regulations, the Declaration of Helsinki, GCP, and this study protocol. Amendments to the protocol will only be made after notifying the sponsor and obtaining its approval and will be implemented upon approval from the Ethics Committee, except where necessary to protect the subjects' safety, rights, and interests. I will be responsible for making medical decisions related to the study to ensure that the subjects can be timely and appropriately treated in case of any adverse events during this study. These adverse events will be recorded and reported under relevant national regulations. I am responsible for ensuring that the data on the study medical record is documented truly, accurately, completely and promptly. I will accept the monitoring and auditing of the monitor or auditor designated by the sponsor and inspections by drug regulatory authorities to ensure the quality of the study. I promise to keep the subject's personal information and related matters confidential. I agree to disclose my full name and profession to the sponsor and the expenses related to the study as required and refrain from engaging in commercial and economic activities related to this study. I agree that the study results will be used for drug registration and publication. I will submit a principal investigator curriculum vitae to the Ethics Committee and the Drug Regulatory Authority (for filing) before the start of the study.

**Test Facility:**

| Prof. Jun Zhu | | |
|---|---|---|
| (print) | (Signature) | Date of signature (DD-MM-YYYY) |

## Principal Investigator Protocol Signature Page

I will carefully perform the investigator's duties per the provisions under Good Clinical Practice (hereinafter referred to as GCP). I will provide a copy of the protocol to my study team and discuss it to ensure that they fully understand this study. In addition, I will personally participate in or directly guide the study. I have received the Investigator's Brochure for the study drug in this study, and I have known and read the preclinical study of the study drug and the protocol of the study (Protocol No.: JS004-002-I, Version No.: 2.1, Version date: 15-Feb-2022). I agree to perform the relevant duties in strict accordance with China's relevant laws and regulations, the Declaration of Helsinki, GCP, and this study protocol. Amendments to the protocol will only be made after notifying the sponsor and obtaining its approval and will be implemented upon approval from the Ethics Committee, except where necessary to protect the subjects' safety, rights, and interests. I will be responsible for making medical decisions related to the study to ensure that the subjects can be timely and appropriately treated in case of any adverse events during this study. These adverse events will be recorded and reported under relevant national regulations. I am responsible for ensuring that the data on the study medical record is documented truly, accurately, completely and promptly. I will accept the monitoring and auditing of the monitor or auditor designated by the sponsor and inspections by drug regulatory authorities to ensure the quality of the study. I promise to keep the subject's personal information and related matters confidential. I agree to disclose my full name and profession to the sponsor and the expenses related to the study as required and refrain from engaging in commercial and economic activities related to this study. I agree that the study results will be used for drug registration and publication. I will submit a principal investigator curriculum vitae to the Ethics Committee and the Drug Regulatory Authority (for filing) before the start of the study.

**Test Facility:**

| Prof. Jun Ma | | |
|---|---|---|
| (print) | (Signature) | Date of signature (DD-MM-YYYY) |

# Table of Contents

Table of Contents ........ 6
List of Tables ........ 11
List of Figures ........ 12
Synopsis ........ 16
1. Introduction: Study Background and Scientific Rationale ........ 42
1.1. Study Background ........ 42
1.1.1. Drug Name ........ 43
1.1.2. Pharmacological Type and Mechanism of Action ........ 44
1.1.3. Pharmacodynamic Study ........ 44
1.1.4. Toxicology Study ........ 46
1.1.5. Tissue Cross-Reactivity in Human and Cynomolgus Monkeys ........ 50
1.1.6. Safety Pharmacology ........ 50
1.1.7. Pharmacokinetic Study ........ 50
1.1.8. Clinical Study Progress ........ 55
1.2. Scientific Rationale ........ 63
1.3. Potential Risks and Benefits ........ 63
2. Study Objectives and Endpoints ........ 64
2.1. Study Objectives ........ 64
2.2. Study Endpoints ........ 64
3. Study Design ........ 65
3.1. Overall Design ........ 65
3.1.1. Determination of Starting Dose for Escalation ........ 69
3.1.2. Determination of Maximum Dose for Escalation ........ 70
3.1.3. Dose Escalation Design ........ 71
3.1.4. Dose Escalation Principle ........ 71
3.2. Definition of DLT ........ 71
3.3. Maximum Tolerated Dose (MTD) and Recommended Phase II Dose (RP2D) ........ 73
3.4. Dose Expansion Phase ........ 73
3.5. Indication Expansion Phase ........ 74
3.6. Safety Monitoring Committee ........ 74
3.7. Safety Meeting ........ 75
3.8. Pharmacokinetic and Pharmacodynamic Studies ........ 75
3.9. Cytokine Study ........ 76

3.10. Immune Cell Subtype Study ........ 76
3.11. Immunogenicity Study ........ 76
3.12. Biomarker Study (Optional) ........ 77
4. Selection of Subjects ........ 77
4.1. Inclusion Criteria ........ 77
4.2. Exclusion Criteria ........ 78
4.2.1. Lifestyle Requirements ........ 81
4.3. Subject’s Withdrawal ........ 81
4.3.1. Criteria for Subject's Withdrawal ........ 81
4.3.2. Procedures for Subject‘’s Withdrawal from the Study ........ 82
4.3.3. Replacement of Early Withdrawn Subjects ........ 82
4.3.4. Identification of Subjects ........ 82
4.4. Early Termination or Suspension of Study ........ 82
5. Study Drug ........ 83
5.1. Drug Dispensation ........ 83
5.2. Supply of study drug ........ 83
5.2.1. Dosage form and Strength ........ 83
5.2.2. Preparation and Dispensation of study drug ........ 83
5.3. Dosing ........ 83
5.3.1. Dosing Regimen for JS004 Monotherapy ........ 83
5.3.2. Dosing Regimen for JS004 Combined with Toripalimab ........ 84
5.3.3. Dose Adjustment ........ 84
5.3.4. Criteria for Dose Resumption ........ 86
5.4. Storage of Study Drug ........ 86
5.5. Concomitant Medications ........ 87
5.5.1. Permitted Medications ........ 87
5.5.2. Prohibited Medications ........ 87
6. Study Procedures ........ 88
6.1. Screening Period (Day -28 to Day 1) ........ 88
6.2. Treatment Period ........ 90
6.3. End of treatment ........ 92
6.4. Follow-up and Unscheduled Visits ........ 93
7. Assessment ........ 94
7.1. Safety Assessment ........ 94

7.1.1. Adverse event assessment ....94
7.1.2. Clinical Laboratory Assessment ....94
7.1.3. Vital Signs ....95
7.1.4. History ....96
7.1.5. Physical Examination ....96
7.1.6. Electrocardiogram ....96
7.2. Pharmacokinetic (PK), Pharmacodynamic (PD), Cytokine, Immune Cell Subtype, Immunogenicity and Biomarker Studies ....97
7.2.1. PK Sample Collection ....97
7.2.2. PD Sample Collection ....97
7.2.3. Sample Collection for Cytokine Study ....97
7.2.4. Sample Collection for Immune Cell Subtype Study ....97
7.2.5. Immunogenicity Sample Collection ....97
7.2.6. Biomarker Sample Collection (Optional) ....98
7.3. Efficacy Assessment ....98
8. Adverse Events ....99
8.1. Definitions ....99
8.1.1. Description of Adverse Events ....99
8.1.2. Definition of Serious Adverse Events ....99
8.2. Recording and Reporting of Adverse Events ....100
8.2.1. Time Period for Reporting of Adverse Events ....100
8.2.2. Follow-up of Adverse Events ....101
8.2.3. Serious adverse event report ....101
8.2.4. E Laboratory Test Abnormality ....102
8.2.5. Progressive Disease ....102
8.2.6. Death ....102
8.2.7. Pregnancy Report ....103
8.2.8. Drug Overdose Reporting ....103
8.3. Criteria for Severity Grading of Adverse Events ....103
8.4. Criteria for Relationship of Adverse Events to the Study Drug ....104
8.5. Toxicity Management for Specific Adverse Events ....105
8.5.1. Infusion reactions ....105
8.5.2. Hypersensitivity ....105
8.5.3. Extravasation ....106
8.5.4. Immune-related adverse events (irAE) ....106

9. Data Analysis/Statistical Method ....... 107
9.1. Analysis Set ....... 107
9.2. Efficacy Evaluation ....... 108
9.3. Sample size determination ....... 109
9.4. PK/PD/Cytokine/Immune Cell Subtype/Immunogenicity/Biomarker Study Analyses ....... 109
9.4.1. PK Analysis ....... 109
9.4.2. PD Analysis ....... 109
9.4.3. Cytokine Analysis ....... 110
9.4.4. Immune Cell Subtype Analysis ....... 110
9.4.5. Immunogenicity Analysis ....... 110
9.4.6. Biomarker Endpoint Analysis ....... 110
9.5. Safety Analysis ....... 110
9.5.1. Adverse Events ....... 110
9.5.2. Primary Endpoint Analysis ....... 111
9.5.3. Laboratory Abnormality ....... 111
9.5.4. ECG ....... 111
10. Data Management Methods ....... 111
10.1. Data Recording ....... 111
10.1.1. Completion of Study Medical Records ....... 111
10.1.2. eCRF Completion ....... 111
10.1.3. eCRF Review ....... 112
10.2. Data Monitoring ....... 112
10.3. Data Management ....... 112
10.3.1. Establishment of EDC Database ....... 112
10.3.2. Data Entry and Reconciliation ....... 112
10.3.3. Data Review and Database Lock ....... 112
10.3.4. Data Archiving ....... 113
11. Termination and End of Study ....... 113
11.1. Criteria for Study Termination by the Sponsor ....... 113
11.2. End of Study ....... 113
12. Regulatory Ethics, Informed Consent and Subject Protection ....... 113
12.1. Regulatory Considerations ....... 113
12.2. Ethical Norms ....... 114
12.3. Institutional Review Board/ Independent Ethics Committee ....... 114

12.4. Informed Consent ........ 115
12.4.1. Informed Consent ........ 115
12.4.2. Informed Consent Process and Records ........ 115
12.5. Confidentiality of Subject Information ........ 116
13. Confidentiality and Publication of Study Results ........ 117
14. Clinical trial progress ........ 117
15. References ........ 118
Annex 1 Lugano 2014 Criteria ........ 120
Annex 2 Eastern Cooperative Oncology Group Performance Status ........ 123
Annex 3 International Prognostic Index (IPI) score ........ 124
Annex 4 ASCO Guidelines Management of Immune-Related Adverse Events (irAE) ........ 125
Annex 5 US NIAID and FAAN Diagnostic Guidelines for Anaphylaxis ........ 127
Annex 6 Bone Marrow Distribution in Adults ........ 128
Annex 7 Traditional Chinese Medicine (TCM) Prohibited during the Trial ........ 130

## List of Tables

Table 1 Schedule of Activities ........ 33
Table 2 Sampling Schedule for PK/PD/Cytokine/Immune Cell Subtype Studies ........ 39
Table 3 Sampling Schedule for Vital Signs/ECG ........ 41
Table 4 Effect of JS004 on MC38-hBTLA Mouse Colon Cancer in Human BTLA Transgenic Mice ........ 45
Table 5 Effect of JS004 on MC38-hBTLA Mouse Colon Cancer Tumor Volume in Human BTLA Transgenic Mice ........ 46
Table 6 Summary of Toxicity Studies ........ 47
Table 7 Summary of Single-dose Toxicity Studies ........ 48
Table 8 Summary of Single-Dose Pharmacokinetic/Toxicokinetic Studies ........ 51
Table 9 Mean Pharmacokinetic Parameters of JS004 in Cynomolgus Monkeys after a Single Intravenous Dose ........ 52
Table 10 Mean Pharmacokinetic Parameters of Serum JS004 after Repeated Intravenous Doses ........ 53
Table 11 Mean Serum Toxicokinetic Parameters of JS004 after a Single Intravenous Dose ........ 53
Table 12 Summary of the Repeat-Dose Toxicokinetic Studies ........ 54
Table 13 Comparison of Exposure in Female and Male Cynomolgus Monkeys after Repeated Doses of JS004 ........ 55
Table 14 Exposure and Dose Proportionality after Repeated Doses of JS004 ........ 55
Table 15 Cumulative Exposure of JS004 (D29 vs D1) ........ 55
Table 16 Summary of Adverse Events for TAB-004-01 ........ 57
Table 17 Summary of Serious Adverse Events for TAB-004-01 ........ 57
Table 18 Study Drug-related Adverse Events Occurring in ≥8% of Subjects in TAB-004-01 58
Table 19 Pharmacokinetic Parameters of TAB004 Following Intravenous Infusion ........ 59
Table 20 Summary of Adverse Events for JS004-001-I ........ 61
Table 21 Summary of Serious Adverse Events for JS004-001-I ........ 61
Table 22 Study Drug-related Adverse Events in JS004-001-I ........ 61
Table 23 Pharmacokinetic Parameters of JS004 after Intravenous Drip in Cycle 1 (n=3) ........ 63
Table 24 JS004 Dose Escalation Design ........ 71
Table 25 NCI-CTCAE V5.0 Common Criteria for Severity of AEs ........ 103
Table 26 Management of Infusion-Related Reactions (NCI CTCAE Grading) ........ 105
Table 27 Management of Allergic Reactions/Hypersensitivity (NCI CTCAE Grading) ........ 106

## List of Figures

Figure 1 Effect of JS004 on MC38-hBTLA Mouse Colon Cancer Tumor Progression in Human BTLA Transgenic Mice ........ 46
Figure 2 Serum Concentration Profiles of JS004 in Cynomolgus Monkeys Following Repeated Doses of JS004 by Intravenous Infusion ........ 54
Figure 3 Mean Serum Concentration Versus Time Curve after Intravenous Infusion of TAB004 (C1: Cycle 1; C4: Cycle 4) ........ 59
Figure 4 Mean Serum Concentration Versus Time Curve of JS004 after Intravenous Infusion in Cycle 1 ........ 62
Figure 5 Overall Study Design Diagram ........ 68

## List of Abbreviations

| Abbreviations | Description |
|---|---|
| 12-Lead ECG | 12-Lead electrocardiogram |
| AE | Adverse event |
| ALP | Alkaline phosphatase |
| ALT | Alanine aminotransferase |
| ANOVA | Analysis of variance |
| AST | Aspartate aminotransferase |
| AUC | Area under the serum concentration |
| $AUC_{0-\infty}$ | Area under the serum concentration versus time curve from time zero to infinity |
| $AUC_{0-t}$ | Area under the serum concentration versus time curve from time zero to the last measurable concentration |
| $AUC_{0-21d}$ | Aarea under the serum concentration versus time curve from time zero to 21d |
| BUN | Blood urea nitrogen |
| CK | Creatine kinase |
| CK-MB | Creatine kinase-MB |
| $Cl^-$ | Blood chlorine |
| CL | Clearance |
| $C_{max}$ | Maximum serum concentration |
| $C_{min}$ | Minimum serum concentration |
| Cr | Creatinine |
| CRF | Case report form |
| D | Day |
| DLT | Dose-limiting toxicity |
| EC | Ethics committee |
| EOI | End-of-Infusion |

| | |
|---|---|
| EOT | End-of-Treatment Visit |
| Fab | Fragment of antigen binding |
| GCP | Good Clinical Practice |
| GLU | Blood glucose |
| GLU-U | Urine glucose |
| h | Hour |
| Hb | Hemoglobin |
| HDL-C | High-density lipoproteincholesterol |
| IB | Investigator's brochure |
| $IC_{50}$ | Half maximal inhibitory concentration |
| LEU | Leukocytes in urine |
| IPI | International Prognostic Index |
| IRR | Infusion related reaction |
| ITIM | Immunoreceptor tyrosine-based inhibitory motif |
| IU | International unit |
| K+ | Serum potassium |
| KET | Urine acetone bodies |
| kg | Kilogram |
| LDH | Lactate dehydrogenase |
| LDL-C | Low-density lipoprotein cholesterol |
| MFD | Maximum feasible dose |
| mAb | Monoclonal antibody |
| mg | Milligram |
| min | Minimum |
| mL | Milliliter |
| mm | Millimeter |
| MTD | Maximum tolerated dose |
| $Na^+$ | Plasma sodium |
| NEUT | Neutrophil |
| NOAEL | No-observed-adverse-effect level |

| | |
|---|---|
| OBD | Optimal biological dose |
| PBMC | Peripheral blood mononuclear cell |
| PLT | Blood platelet |
| PMBL | Primary mediastinal large B cell lymphoma. |
| PRO | Protein in urine |
| RAC | Accumulation ratio |
| RBC | Red blood cell count |
| RP2D | Recommended Phase 2 Dose |
| sec | Second |
| SAE | Serious adverse event |
| SAP | Statistical analysis plan |
| SHP | Protein tyrosine phosphatase with two src-homology 2 |
| sUA | Serum uric acid |
| T-BIL | Total bilirubin |
| TC | Total cholesterol |
| TG | Triglyceride |
| $T_{max}$ | Time to reach maximum serum concentration |
| $T_{1/2}$ | Terminal half-life |
| Vss | Volume of distribution |
| UA | Uric acid |
| UBIL | Urine bilirubin |
| URBC | Urine red blood cell |
| WBC | White blood cell count |

# Synopsis

| | |
|---|---|
| **Study Title** | Phase IStudy of Recombinant Humanized Anti-BTLA Monoclonal Antibody (JS004) in Patients with Relapsed/Refractory Malignant Lymphoma |
| **Protocol Number** | JS004-002-I |
| **Version Number** | 2.1 |
| **Version date** | 15-Feb-2022 |
| **Study Drug** | **JS004** (recombinant humanized anti-BTLA monoclonal antibody injection)<br>Strength: 100 mg/5 mL/vial.<br>Usage: intravenous drip, once every 3 weeks.<br>**Toripalimab (trade name: Tuoyi)**<br>Strength: 240 mg/6 mL/vial.<br>Usage: intravenous drip, once every 3 weeks. |
| **Study Background and Scientific Rationale** | JS004 (also known as TAB004) is a recombinant humanized IgG4k monoclonal antibody specific for human B and T lymphocyte attenuator (BTLA). BTLA is expressed on T and B lymphocytes and inhibits T cell proliferation and cytokine production upon activation by its ligand herpesvirus entry mediator (HVEM). JS004 incorporates a serine substitution for proline at site 227 in the heavy chain hinge region (S227P), aiming to minimize Fab exchange and improve molecular stability. JS004 specifically and effectively blocks the interaction between HVEM and BTLA, relieving the inhibitory effect on T cell-mediated immune responses. Preclinical studies suggest that blocking BTLA-mediated signaling may improve the function of T cells in tumor patients and enhance anti-tumor immunity.<br>JS001 (toripalimab), a newly developed recombinant humanized (up to 97%) anti-PD-1 monoclonal antibody, is a human IgG4/Kappa subtype. Tumor cells can escape from the body's immune surveillance and killing by highly expressing PD-L1 molecules that mediate negative regulatory signals by binding to PD-1 molecules on T lymphocytes, which leads to induced apoptosis and immune incompetence of tumor antigen-specific T cells.<br>Studies have shown that PD1 prefers to recruit SHP-2, while BTLA prefers to recruit SHP-1. PD-1 has an effect on both TCR and CD28 signaling pathways upon binding to its ligand PD-L1. In the absence of SHP-2 in cells, SHP-1, instead of SHP-2, can mediate the effect of PD-1 on TCR and CD28 signaling pathways, suggesting redundancy in T-cell cosuppressors and theoretically supporting the combined targeting of PD-1 and BTLA. |

| | |
|---|---|
| **Study Objectives** | **Primary Objectives:**<br>• To evaluate the safety and tolerability of JS004 monotherapy and in combination with toripalimab in patients with relapsed/refractory malignant lymphoma;<br>• To observe the dose-limiting toxicity (DLT) of JS004 monotherapy and in combination with toripalimab in patients with relapsed/refractory malignant lymphoma and to determine the maximum tolerated dose (MTD) and recommended phase II dose (RP2D). When no MTD is observed, the maximum dose of JS004 specified in the protocol is taken as the maximum feasible dose (MFD). |
| | **Secondary Objectives:**<br>• To evaluate the pharmacokinetic (PK) profile of JS004 monotherapy and in combination with toripalimab;<br>• To evaluate BTLA receptor occupancy (RO) on peripheral blood immune cells;<br>• To evaluate the effect of JS004 monotherapy and in combination with toripalimab on peripheral blood cytokines;<br>• To preliminarily evaluate the anti-tumor activity of JS004 monotherapy and in combination with toripalimab, including objective response rate (ORR), duration of response (DoR), disease control rate (DCR), progression-free survival (PFS) and overall survival (OS);<br>• To evaluate the immunogenicity of JS004 monotherapy and in combination with toripalimab. |
| | **Exploratory Objectives:**<br>• To evaluate the effect of JS004 monotherapy and in combination with toripalimab on peripheral blood immune cell subtypes;<br>• To investigate biomarkers possibly associated with JS004 activity, including BTLA, HVEM and PD-L1, their expression in tumor tissues, the CD8 positive tumor-infiltrating immune cell density, whole exome sequencing (WES) of tumor tissue and paired blood, peripheral blood immune cell surface receptors (PD-1, HVEM, CTLA-4, CD112R, TIM-3, ICOS, CX3CR1, CD183, and CD103), and their relationship with treatment response. |

| | |
|---|---|
| **Endpoints** | **Primary Endpoints**<br>• Safety evaluation: Incidence and severity of adverse events/serious adverse events (graded according to CTCAE 5.0); and incidence of Grade ≥3 adverse events;<br>• MTD and RP2D (RP2DA for monotherapy and RP2DB for combination).<br>**Secondary Endpoints**<br>• PK parameters: including but not limited to maximum serum concentration ($C_{max}$), minimum serum concentration ($C_{min}$), time to reach maximum serum concentration ($T_{max}$), area under the serum concentration versus time curve from time zero to infinity ($AUC_{0-\infty}$), area under the serum concentration versus time curve from time zero to the last measurable concentration timepoint ($AUC_{0-t}$), area under the serum concentration versus time curve from time zero to 21d ($AUC_{0-21d}$), terminal half-life ($t_{1/2}$), clearance (CL), volume of distribution ($V_{ss}$) and accumulation ratio (Rac), etc.;<br>• Pharmacodynamic assessment: BTLA receptor occupancy (RO) on CD3+, CD4+CD45RA+, CD8+CD45RA+, CD3-CD20+, and CD3-CD56+ cells;<br>• Effect of JS004 and in combination with toripalimab on peripheral blood cytokines (IFN-γ, IL-8, IL-4, IL-6, IL-10, and TNF-α);<br>• Efficacy evaluation: Objective response rate (ORR), duration of response (DoR), disease control rate (DCR), progression-free survival (PFS) and overall survival (OS) evaluated per Lugano 2014 Criteria;<br>• Immunogenicity assessment: Anti-drug antibodies (ADAs) and/or neutralizing antibodies (Nabs).<br>**Exploratory Endpoints**<br>• Effect of JS004 monotherapy and in combination with toripalimab on peripheral blood immune cell subtypes (CD4+, CD3+CD4+/CD3+CD8+, CD20+, CD3-CD20-NKG2A+ cells, etc.);<br>Biomarkers possibly associated with JS004 activity, including BTLA, HVEM and PD-L1, their expression in tumor tissues, the CD8 positive tumor-infiltrating immune cell density, whole exome sequencing (WES) of tumor tissue and paired blood, peripheral blood immune cell surface receptors (PD-1, HVEM, CTLA-4, CD112R, TIM-3, ICOS, CX3CR1, CD183, and CD103), and their relationship with treatment response. |

| | |
|---|---|
| **Subjects** | Patients with relapsed/refractory malignant lymphoma (peripheral T-cell lymphoma, Hodgkin lymphoma, follicular lymphoma, etc. are preferred), including 1) those who relapse and progress after autologous stem cell transplantation; 2) those who fail to undergo autologous stem cell transplantation must have failed systemic second-line treatment, including no response (SD or PD), or relapse and progression (patients with peripheral T-cell/NK-cell lymphoma and mediastinal large B-cell lymphoma can be enrolled after failure of first-line treatment); 3) those with B-cell lymphoma must have previously received rituximab. |
| **Sample Size** | A total of approximately 170 subjects are planned to be enrolled in this study.<br>**Part A:** Approximately 9-18 subjects are planned to be enrolled in the dose escalation study, and approximately 18 subjects are planned to be enrolled in the expansion study of the selected dose group. Peripheral T-cell lymphoma, Hodgkin lymphoma, follicular lymphoma or other subtypes are preferentially selected in the indication expansion phase, which can be adjusted according to study progress. Approximately 15 subjects are enrolled for each indication (including the number of subjects in the dose expansion phase, which can be adjusted according to study progress). Approximately 80 subjects are expected to be enrolled in Part A.<br>**Part B:** There are 2 dose groups for dose escalation of the combination treatment, with 3-6 subjects in each dose group, i.e., 6-12 subjects are expected to be enrolled in total. Then, the recommended dose will be determined by the sponsor and the investigator upon discussion, and the enrollment will be extended to 6-18 subjects (If the recommended dose is studied in 2 dose groups, 6-9 subjects will be enrolled in each group). Peripheral T-cell lymphoma, Hodgkin lymphoma, follicular lymphoma or other subtypes are preferentially selected in the indication expansion, which can be adjusted according to study progress. Approximately 20 subjects will be enrolled for each indication (including the number of subjects in the dose expansion phase. The specific number of subjects can be adjusted according to study progress. Approximately 90 subjects are expected to be enrolled in this phase. |
| **Study Design** | This is a multicenter, open-label, dose escalation and dose expansion phase Istudy of JS004 in Chinese patients with relapsed/refractory malignant lymphoma. The study comprises two parts: Part A and Part B, and each consists of three phases: dose escalation, dose expansion and indication expansion. The study process includes a screening period of up to 28 days (from the subject's signature of the informed consent until before the first dose of the study drug), a treatment period (from the first dose of the study drug to drug discontinuation), and a follow-up period (safety follow-up and survival follow-up). |

| | |
|---|---|
| | **Part A: JS004 Monotherapy**<br>1) Dose escalation phase: The "3+3" dose escalation design is adopted, and 3 dose groups are planned, including 1 mg/kg, 3 mg/kg and 10 mg/kg. All doses are administered by intravenous drip. The 10 mg/kg dose is the pre-specified maximum administered dose (MAD) with an interval of 3 weeks between each administration. However, the dose level or administration interval may be adjusted during the study after new data becomes available.<br>Eligible subjects are enrolled sequentially and receive escalating doses from low to high. Each subject can only be assigned to one dosing regimen or interval schedule. There is an interval of at least 7 days between the first and second subjects in each dose group. The DLT observation period for each subject is 3 weeks after the first dose. If none of the 3 subjects in a dose group experiences DLT within 3 weeks, the next dose escalation may be performed. If 1 of 3 subjects experiences DLT, 3 additional subjects should be included. The next dose escalation will be performed if no DLT is observed in the 3 additional subjects. If ≥1 DLT event recurs in the additional 3 subjects, dose escalation will be terminated, and the previous dose will be defined as the MTD. After completing DLT observation in the highest dose group specified in the protocol, dose escalation will not be continued even if the above discontinuation criteria are not met.<br>2) Dose expansion phase: The dose level in this phase will be determined to be 3 mg/kg according to the PK, PD and safety data of different dose groups in the dose escalation phase and JS004 Monotherapy Phase I Study in the US (TAB004-001). Additionally, the body weight of the monoclonal antibody may not be a key factor affecting its exposure, JS004 has large safety margins, and no clear relationship has been found between the body weight of the patient and the PK and efficacy/safety of the drug within the investigated dose range, as well as the mean body weight of the subject is 60 kg. So, a fixed dose group of 200 mg (approximately equivalent to 3 mg/kg) will be added in this phase to further confirm the safety and pharmacokinetic characteristics of monotherapy and explore the mode of administration of a fixed dose to simplify the clinical administration of JS004. This phase can be started after the completion of escalation in the corresponding dose group, with 6-9 subjects enrolled in each dose group. The number of subjects can be adjusted subsequently according to study progress. In this phase, the requirements for subject inclusion, mode of administration and study procedures are the same as those in the first phase, and evaluations will also be performed for the safety, tolerability, PK, PD, immunogenicity and preliminary efficacy. Any DLT or delayed DLT that may occur in this phase will not be used as the basis for dose escalation, but as a reference for subsequent clinical studies in terms of dose design. |

| | |
|---|---|
| | Based on all safety, PK, and PD data during dose escalation, the RP2D may be the MTD/MAD or the dose below the MTD/MAD. The RP2D will be determined based on the study progress. Any delayed immune-related DLT occurring after the first period of treatment may also be regarded as a reference for RP2DA assessment. The study in this phase can be conducted concurrently with that in the Part B dose escalation phase.<br>3) Indication expansion phase: Peripheral T-cell lymphoma, Hodgkin lymphoma, follicular lymphoma or other subtypes are preferred for indication expansion, which can be adjusted according to study progress. Approximately 15 subjects are enrolled for each indication expansion (including the number of subjects in the dose expansion phase; the number of cases can be adjusted according to study progress). All doses are administered by intravenous injection at RP2DA, Q3W. Treatment will continue in a 21-day cycle until progressive disease, intolerable toxicity, treatment with JS004 (since the first dose) for more than 2 years or other reasons as specified in the protocol. During this phase, all study procedures will remain the same as in the dose expansion phase, with the exception that intensive PK (pharmacokinetic) sampling will not be carried out and blood samples for PD (pharmacodynamic), cytokine, and immune cell subtype studies will not be collected.<br>**Part B: JS004 in Combination with Toripalimab**<br>1) Dose escalation phase: The "3+3" dose escalation design is adopted, and 2 dose groups are preliminarily planned. Toripalimab is administered at 240 mg, and JS004 at a fixed dose of 100 mg and 200 mg, with an interval of Q3W. The DLT observation period is 21 days after the first dose.<br>Each dose group will first include 3 subjects. If the first subject experiences no DLT within the DLT evaluation window (21 days) at a given dose, the investigator and the sponsor will discuss and decide to proceed to the next dose level (dose escalation is not allowed in the same subject). If 1 of 3 subjects experiences DLT, 3 additional subjects will be enrolled in the current dose group; If DLT occurs in ≥2 subjects in a dose group of 3 or 6 subjects, dose escalation will be terminated, and the investigator and the sponsor will decide whether to terminate dose escalation or consider dose modification for exploration upon discussion. The maximum tolerated dose (MTD) is defined as the maximum dose at which DLT occurs in <1/3 of the patients. The dose level, administration interval and number of enrolled subjects may be adjusted by the Safety Monitoring Committee (SMC) upon discussion during the study according to the progress of other studies of JS004 and the safety, PK and PD data available. |

| | |
|---|---|
| | 2) Dose expansion phase: The recommended dose group of the combination is selected for expansion, and 6-18 subjects are enrolled (if there are two dose groups for the recommended dose, 6-9 subjects will be enrolled in each group). Subsequently, the number of subjects will be adjusted according to study progress to better confirm the safety of the combination and determine the recommended dose for expansion (RDE) for the indication expansion phase.<br>The RP2DB will be discussed and determined by the Safety Monitoring Committee (SMC) based on all safety, PK, PD and other data obtained during dose escalation and in this phase, as well as the progress of other studies of JS004. If the subject is tolerable at the discretion of the investigator after completing the DLT evaluation, the subject will continue to be administered at the same dose until progressive disease, intolerable toxicity, treatment with JS004 and/or toripalimab (since the first dose) for more than 2 years or other reasons specified in the protocol.<br>3) Indication expansion phase: Peripheral T-cell lymphoma, Hodgkin lymphoma, follicular lymphoma or other subtypes are preferred for indication expansion, which can be adjusted according to study progress. Approximately 20 subjects are enrolled for each indication expansion (including the number of subjects in the dose expansion phase). The number of cases can be adjusted according to study progress. All doses are administered by intravenous injection at RP2DB, Q3W, in combination with toripalimab 240 mg, Q3W. Treatment will continue in a 21-day cycle until progressive disease, intolerable toxicity, treatment with JS004 and/or toripalimab (since the first dose) for more than 2 years or other reasons as specified in the protocol. During this phase, all study procedures will remain the same as in the dose expansion phase, with the exception that intensive PK (pharmacokinetic) sampling will not be carried out and blood samples for PD (pharmacodynamic), cytokine, and immune cell subtype studies will not be collected.<br>In Part A, subjects who have progressive disease but are still benefiting from anti-BTLA treatment, as confirmed by the investigator, or who have stable disease for at least two tumor assessments, may proceed to Part B to receive the recommended dose of the combination with toripalimab (subjects with progressive disease should provide informed consent for continuing treatment after progression). Once enrolled in Part B, the study procedures will follow the protocols outlined for this phase. |
| Pharmacokinetic, Pharmacodynamic, Cytokine, Immune Cell Subtype, Immunogenicity and Biomarker Studies | **Pharmacokinetic Study** |

**JS004 PK sample collection:**

Dose escalation and dose expansion phases of Part A and Part B:

PK parameters, including but not limited to $AUC_{0-\infty}$, $AUC_{0-t}$, $AUC_{0-21d}$, $C_{max}$, $C_{min}$, $T_{max}$, $t_{1/2}$, CL, $V_{ss}$ and Rac, will be calculated using the non-compartmental model of WinNonlin 6.4 or above. The linearity between primary PK parameters ($C_{max}$ and AUC) and doses will be assessed.

Pharmacokinetic (PK) blood sampling time: Intensive blood sampling will be performed within 1 h before the first dose of JS004, at the end of the first dose (+5 min), 1 h (±5 min) after the end of the first dose, 6 h (±15 min), 24 h (1 h), 48 h (±2 h), 168 h (Day 8, ±8 h), 336 h (Day 15, ±12 h) and 504 h (Day 22, ±24 h, i.e., before administration in Cycle 2) after the start of the first dose; within 1 h before administration in Cycle 2 (sample collection may not be repeated if there is no delay in Cycle 2) and at the end of dose (+5 min); In Cycle 4, intensive blood sampling will be performed within 1 h before administration, after the end of administration (+5 min), 1 h (± 5 min) after administration, 6 h (± 15 min), 24 h ±(1 h), 48 h ±(2 h), 168 h (Day 8, ±8 h), 336 h (Day 15, ± 12 h), and 504 h (Day 22, ±24 h, i.e., before administration in Cycle 5) after the start of administration; within 1 h before administration and at the end of administration in Cycles 6, 8, 12, 16, and within 1 h before administration, at the end of treatment visit (±7 days), and at the safety follow-up visit (if applicable) every 8 cycles thereafter. The blood collection volume will be 3 mL.

Indication expansion phase of Part A and Part B:

Three millilters of blood samples will be collected to detect the blood concentration of JS004. Blood sampling timepoints: within 1 h before the dose of JS004 in Cycles 1, 2, 4, 6, 8, 12, 16, and every 8 cycles thereafter, at the end of treatment visit (±7 days), and at the safety follow-up visit (if applicable).

Toripalimab PK sample collection:

Two millilters of blood samples will be collected to detect the blood concentration of toripalimab. Blood sampling timepoints: within 1 h before the dose of JS004 in Cycles 1, 2, 4, 6, 8, 12, 16 and every 8 cycles thereafter, at the end of treatment visit (±7 days), and at the safety follow-up visit (if applicable).

**Pharmacodynamic Study**

Dose escalation and dose expansion phases of Part A and Part B:

The BTLA receptor occupancy (RO) on CD3+, CD4+CD45RA+, CD8+CD45RA+, CD3-CD20+, and CD3-CD56+ cells will be investigated. Blood sampling timepoints: within 1 h before the first dose of JS004, 24 h (±1 h) and 168 h (Day 8, ±8 h) after the start of the first dose, within 1 h before administration in Cycles 2, 4, 6 and 8, at the end of treatment visit (±7 days) and at the safety follow-up visit. The blood collection volume will be 2 mL.

| | |
|---|---|
| | **Cytokine Study**<br>Dose escalation and dose expansion phases of Part A and Part B:<br>The effect of JS004 and in combination on peripheral blood cytokines (IFN-γ, IL-8, IL-4, IL-6, IL-10, TNF-α) will be investigated. Blood sampling timepoints: within 1 h before the first dose of JS004 and 24 h (±1 h) after the start of the first dose. The blood collection volume will be 2 mL.<br>**Immune Cell Subtype Study**<br>Dose escalation and dose expansion phases of Part A and Part B:<br>The effect of JS004 on peripheral blood immune cell subtypes (CD4+, CD3+CD4+, CD3+CD8+, CD20+, CD3-CD20-NKG2A+ cells, etc.) will be investigated. Blood sampling timepoints: within 1 h before the first dose of JS004, 24 h (±1 h) and 168 h (Day 8, ±8 h) after the start of the first dose in Cycle 1, within 1 h before the dose of JS004 in Cycles 2, 4, 6 and 8, at the end of treatment visit (±7 days) and at the safety follow-up visit. The blood collection volume will be 2 mL.<br>**Immunogenicity Study**<br>Immunogenicity assessment: All subjects in Part A and Part B who have received at least 1 dose of JS004 will be tested for anti-drug antibodies ADA (including prior to the first dose), and a positive result must be confirmed again. All confirmed positive samples will be reported as positive and will continue to be analyzed for the presence of Nab. Anti-toripalimab tests (including the test before the first dose) will be performed for all subjects who have received at least 1 dose of toripalimab in Part B. Positive samples will continue to be analyzed for the presence of Nab. The incidence, titer, neutralizing capacity, duration, and impact on PK, efficacy/safety of JS004 of anti-drug antibodies will be assessed, if applicable.<br>Blood sampling timepoints for immunogenicity study: Within 1 h before the dose of JS004 in Cycles 1, 2, 4, 6, 8, 12, 16, and every 8 cycles thereafter, at the end of treatment visit (±7 days), and at the safety follow-up visit. and at the start, end and 30 days (±3 days) after the end of any infusion reaction for any subject. The blood collection volume will be 5 mL. |

| | |
|---|---|
| | In case of any immune-related adverse event during the study, the investigator may perform unscheduled blood sampling for PK, PD, cytokines, immune cell subtypes, and immunogenicity if necessary.<br>**Biomarker Study**<br>Fresh or archived tissues (within 2 years) will be collected at screening for BTLA, HVEM, detection of PD-L1 expression and CD8 positive tumor infiltrating immune cell density, and WES of tumor tissue. Six milliliters of blood will be collected before the first dose of JS004 in Cycle 1, at each imaging assessment and at the end of treatment visit for tumor biomarker study, including immune cell surface receptors PD-1, HVEM, CTLA-4, CD112R, TIM-3, ICOS, CX3CR1, CD183 and CD103. Two milliliters of blood will be collected before the first dose of JS004 in Cycle 1 for WES detection of tumor tissue paired blood.<br>The specific sample collection volume and processing method are detailed in the site Laboratory Manual. |
| **Dosing Regimen** | JS004 will be administered via intravenous drip once every 3 weeks (Q3W). The dose will depend on the group the subject is enrolled (fixed dose of 1 mg/kg, 3 mg/kg, 10 mg/kg, 100 mg or 200 mg). The dose in the indication expansion phase will be RP2D (RP2DA for monotherapy and RP2DB for combination).<br>As specified in the infusion regimen of JS004 monotherapy, subjects will receive the first infusion of JS004 on Cycle 1 Day 1 over at least 60 min, followed by close observation for acute allergic reactions (at least 6 h of observation) within 24 h after the infusion. In case of any infusion related reaction (IRR), clinical treatment should be performed based on the medical practice and relevant guidelines of this hospital. If no IRR during the first infusion, the subsequent infusion time can be shortened to at least 30 min. The infusion will be performed on Day 1 of each cycle and observed for at least 1 h after the infusion (3 weeks as a cycle). The infusion time is up to 2 h.<br>JS004 in combination with toripalimab will be administered by intravenous infusion, Q3W. JS004 is administered first, followed by toripalimab after an interval of about 1 h. JS004 is administered the same as that of JS004 monotherapy. The first intravenous infusion of toripalimab should be at least 60 min. If the infusion of the first dose is well tolerated, the second dose may be infused in a shortened period of at least 30 min. All subsequent infusions can be administered over at least 30 min if the infusion over 30 min is well tolerated. |

| | |
|---|---|
| **Inclusion Criteria** | Each patient eligible to participate in this study must meet the following criteria<br>1. Understand and voluntarily sign an informed consent form;<br>2. Age 18-70 years (inclusive), male or female;<br>3. Pathologically confirmed malignant lymphoma;<br>4. Patients with relapsed/refractory malignant lymphoma (peripheral T-cell lymphoma, Hodgkin lymphoma, follicular lymphoma, etc. are preferred), including 1) those who relapse and progress after autologous stem cell transplantation; 2) those who fail to undergo autologous stem cell transplantation must have failed systemic second-line treatment, including no response (SD or PD), or relapse and progression (patients with peripheral T-cell/NK-cell lymphoma and mediastinal large B-cell lymphoma can be enrolled after failure of first-line treatment); 3) those with B-cell lymphoma must have previously received rituximab;<br>5. ECOG: 0~1;<br>6. Expected survival ≥12 weeks;<br>7. Having at least one measurable lesion that meets the requirements of Lugano 2014 Criteria;<br>8. Voluntary consent to provide fresh biopsy specimen before treatment or archived specimen within 2 years for patients unable to provide fresh one before treatment;<br>9. The function of vital organs meets the following requirements (no need for transfusion, blood products, hematopoietic stimulating factors or other drugs to correct blood cell counts within 14 days before the first dose):<br>• Absolute neutrophil count (ANC) ≥1.5×$10^9$/L;<br>• Platelets (PL) ≥100X$10^9$/L, PL ≥75×$10^9$/L in patients with bone marrow involvement;<br>• Hemoglobin (Hb) ≥90 g/L;<br>• Total bilirubin (TBIL) ≤1.5×ULN; TBIL ≤2×ULN for patients with liver metastases; Direct bilirubin (dBIL) ≤3.0 mg/dL for patients with Gilbert syndrome;<br>• Alanine aminotransferase (ALT) and aspartate aminotransferase (AST) ≤2.5×ULN; ALT and AST ≤5×ULN for patients with liver metastases;<br>• Serum creatinine (Cr) ≤1.5×ULN, or calculated creatinine clearance (Cockcroft-Gault formula) ≥50 mL/min; |

| | |
|---|---|
| | • International normalized ratio (INR) ≤2.0 and activated partial thromboplastin time (aPTT) ≤1.5×ULN for patients not receiving anticoagulant therapy; A stable dose of anticoagulant drugs for at least 4 weeks without dose adjustment is required for patients receiving anticoagulant therapy (such as low molecular weight heparin or warfarin); QTc interval ≤450 ms for males and ≤470 ms for females calculated according to Fridericia's criteria;<br>10. Female patients of childbearing potential and male patients whose partners of childbearing potential are required to use a medically approved contraceptive method (such as an intrauterine device (IUD), contraceptive pill or condom) during the treatment period and within 3 months after the end of the treatment period of the study; Female patients of childbearing potential must have a negative serum or urine HCG test within 7 days before enrollment in the study and must be non-lactating. |
| **Exclusion Criteria** | Patients who met any of the following criteria will be excluded from the study:<br>1. Patients who are known allergic to the components of large molecular protein preparations, JS004 or toripalimab;<br>2. Having previously been treated with anti-BTLA or anti-HVEM antibodies;<br>3. Participation in other clinical studies within 4 weeks before the first dose of JS004, except for an observational (non-interventional) clinical study or the follow-up period of an interventional study;<br>4. Major surgery (as judged by the investigator) within 4 weeks before the first dose of JS004 or in the recovery period of surgery. Having received anti-tumor chemotherapy (within 6 weeks after the last chemotherapy with nitrosourea or mitomycin), radiotherapy, targeted therapy, immunotherapy or biological therapy within 4 weeks before the first dose of JS004. Having received traditional Chinese medicine or Chinese patent medicine with anti-tumor indications within 2 weeks before the first dose of JS004. Local palliative treatment, such as local surgery or radiotherapy, for isolated lesions is acceptable if no impact on response evaluation is expected;<br>5. Patients who discontinue prior immunotherapy due to immune-related adverse reactions;<br>6. Having received immunosuppressive medications within 4 weeks before the first dose of JS004, except for corticosteroid nasal spray, inhalers, or systemic prednisone ≤10 mg/day or equivalent;<br>7. Having previously received allogeneic bone marrow transplantation or solid organ transplantation; |

| | |
|---|---|
| | 8 Having received a live or live attenuated vaccine within 30 days before the first dose of JS004;<br>9. Suffering from two or more malignancies within 5 years before the first dose of JS004, except for early malignancy (carcinoma in situ or stage I tumors) that have been cured (no relapse for 5 years), such as adequately treated carcinoma in situ of the cervix, basal or squamous cell skin cancer;<br>10. Presence of central nervous system (CNS) involvement that is symptomatic, untreated, or requires continued treatment (including corticosteroids and antiepileptic drugs). Patients who have previously received treatment but are clinically stable for at least 4 weeks before enrollment can be enrolled, excluding patients with evidence of new or expanded metastasis and discontinued steroid therapy;<br>11. Presence of unrelieved toxicities from prior anti-tumor therapy, i.e., having not resolved to baseline, to NCI-CTCAE 5.0 Grade 0 or 1, or levels described in the inclusion and exclusion criteria. Patients with irreversible toxicities (such as hearing loss) that are reasonably expected not to be aggravated by the study drug can be enrolled upon consultation with the medical monitor;<br>12. Active autoimmune disease requiring systemic treatment within the past 2 years (e.g., use of disease-modifying agents, corticosteroids, or immunosuppressive agents), including but not limited to systemic lupus erythematosus, multiple sclerosis, rheumatoid arthritis, inflammatory bowel disease, autoimmune thyroid disease, vasculitis, psoriasis, etc.). However, patients with hypothyroidism, hypoadrenalism or hypopituitarism controlled only by hormone replacement therapy, type I diabetes mellitus, psoriasis or vitiligo not requiring systemic treatment can participate in this study;<br>13. History of anaphylaxis and eczema or asthma uncontrolled by topical corticosteroids;<br>14. History of primary immunodeficiency;<br>15. Comorbidities that cannot be controlled by concomitant treatment within 14 days before the first dose of the study drug, including but not limited to ongoing or active infection requiring systemic anti-infective treatment; Unexplained fever >38.5°C (subjects with neoplastic fever can be included at the discretion of the investigator); Symptomatic congestive heart failure ≥Grade 3 according to New York Heart Association (NYHA) functional classification, poorly controlled hypertension (systolic blood pressure ≥160 mmHg and/or diastolic blood pressure ≥100 mmHg) or pulmonary hypertension, unstable angina, or myocardial infarction, coronary artery bypass grafting or stent implantation within 6 months before the dose of the study drug, ventricular and supraventricular arrhythmias ≥Grade 2; Cerebrovascular accident (CVA) or transient ischemic attack (TIA) within 6 months before the dose of the study drug; Active peptic ulcer disease or gastritis; |

| | |
|---|---|
| | 16. Previous or current interstitial lung disease, obstructive lung disease that severely affects lung function, or current active pulmonary tuberculosis;<br>17. Patients with associated clinical symptoms (dyspnea, wheezing, abdominal distension, etc.), uncontrolled pleural/abdominal effusion or pericardial effusion requiring repeated drainage;<br>18. History of active inflammatory bowel disease (such as Crohn's disease or ulcerative colitis);<br>19. Patients who are known to be human immunodeficiency virus (HIV) positive;<br>20. Patients with evidence of hepatitis B virus (HBV) or hepatitis C virus (HCV) infection. Patients who are HBsAg positive, those who had HBV infection and are HBcAb positive and HBsAg negative but with HBV DNA copies less than the upper limit of normal in the site after treatment, and patients who had HCV infection and a negative HCV RNA test result at screening are eligible for this study;<br>21. Female patients who are pregnant or lactating;<br>22. Patients with rheumatoid arthritis and other arthropathies, Sjogren's syndrome, celiac disease and psoriasis controlled by topical application, as well as those with positive antinuclear antibody (ANA), antithyroid antibody and other serological tests should be evaluated for the involvement of target organs and the need for systemic therapy as judged by the investigator;<br>23. Any other medical (such as respiratory, metabolic, congenital, endocrine or central nervous system diseases, etc.), psychiatric or social factors that may affect the rights and interests, safety, compliance, ability to sign the informed consent, as well as interpretation of study results as judged by the investigator;<br>24. Subjects who have a history of psychotropic drug abuse and are unable to withdraw or have mental disorders. |
| **Withdrawal Criteria** | Subjects must discontinue the study drug if any of the following occurs:<br>1. Withdrawal of informed consent by the subject;<br>2. Clear evidence of radiographic progression;<br>3. Intolerable treatment toxicity;<br>4. Become pregnant during the study; |

| | |
|---|---|
| | 5. Loss to follow-up (defined as the subject failing to receive a specified visit and the investigator failing to contact the subject or his/her family at least 3 times through 2 or more routes within 3 months after the visit) or death;<br>6. Treatment with JS004 for up to 24 months;<br>7. Initiation of another anti-tumor therapy;<br>8. Obvious inability of the subject to comply with the study procedures as judged by the investigator and/or other conditions that necessitate withdrawal from the study;<br>9. Study termination required by the sponsor, investigators or regulatory authorities. |
| **Criteria for Study Termination** | The study may be terminated or suspended prematurely for adequate reasons. The sponsor should submit a written notice stating the reason for premature termination or suspension to the investigators, the National Medical Products Administration (NMPA), and relevant authorities if this study is prematurely terminated or suspended. The principal investigators must immediately report it to the Ethics Committee and provide appropriate reasons.<br>The termination criteria of this study include but are not limited to the following:<br>1. The study brings unexpected, significant, or unacceptable risks to subjects<br>2. Major mistakes are found in the protocol during the study<br>3. The treatment with the study drug/investigational product is ineffective, or it is meaningless to continue the study<br>4. The sponsor decides to terminate the study due to serious lagging enrollment, frequent protocol deviations, etc.<br>In accordance with ICH-GCP guidelines and local regulations, the study may be terminated by the NMPA, Ethics Committee, investigators or sponsor for the rights, safety and health of subjects. |
| **Safety Evaluation** | The safety will be assessed by periodic physical examinations, vital signs, ECOG performance status, laboratory variables (hematology, coagulation test, biochemistry, urinalysis, and pregnancy test), 12-lead ECGs, and AEs. AEs will be graded according to the CTCAE version 5.0. Before each dose escalation, the safety monitoring committee (SMC) comprising the investigators and the sponsor will review the safety data after all subjects in the current dose group have completed the first 21-day cycle to determine whether dose escalation will be continued to the next dose level. Patients should be observed in the clinical site for at least 1 h after the end of infusion (EOI) (at least 6 h after the first injection; longer time may be allowed for other conditions at the discretion of the investigator and based on the need for blood sampling). |

| | |
|---|---|
| **Efficacy Evaluation** | The investigators will evaluate tumor response at baseline and every 9 weeks according to Lugano 2014 Criteria. CT scans, MRIs, physical examinations, or other tests will be performed as needed. The methods for imaging evaluation must be the same as possible at baseline and throughout the study. If initial progressive disease occurs at a post-baseline tumor assessment, a second scan should be performed for confirmation within 4 weeks but no more than 8 weeks in case of rapid deterioration of clinical symptoms.<br>Parameters to be assessed for anti-tumor activity include the following measures:<br>• Best overall response: CR, PR, SD, or PD (including (immune unconfirmed progressive disease [iUPD] and immune confirmed progressive disease [iCPD]);<br>• Objective response rate: CR+PR;<br>• DoR;<br>• PFS and OS. |
| **Data Analysis and Statistical Methods** | The primary objectives of this study are to evaluate the safety and tolerability of JS004, observe DLTs, and determine the MTD and RP2D. Secondary objectives are PK/PD/immunogenicity and efficacy studies. Therefore, the primary analysis will be summarized using descriptive statistics in the study, without any formal hypothesis test. Any hypothesis test or model-based statistical explorations will only be supplemented as supportive analyses.<br>In this study, efficacy data will be summarized using descriptive statistics according to the following general principles unless otherwise specified.<br>Bi-categorical and multi-categorical data will be summarized using frequencies and percentages. In addition, multi-categorical ordinal data will be summarized using cumulative frequencies and percentages.<br>Quantitative data will be summarized using mean, standard deviation, median, maximum and minimum. For time-to-event data, the Kaplan-Meier method will be used to estimate survival time and median survival.<br>• Serum concentration data and PK parameters will be summarized using mean, geometric mean, standard deviation, coefficient of variation, geometric coefficient of variation, median, maximum, and minimum. |

| | |
|---|---|
| **Study Progress** | Date of first subject enrollment: Aug-2020<br>Expected date of last subject enrollment: Jun-2022<br>Expected date of end of study: Jun-2023 |

**Table 1 Schedule of Activities**

| Study phase | Screening period | Treatment Period | | | | | | End of treatment visit[23] | Safety follow-up[24] | Survival follow-up[25] |
|---|---|---|---|---|---|---|---|---|---|---|
| | | Cycle 1 (21 days) | | | | | Cycle 2 and above (21 days/cycle) | | | |
| Study day | From -D28 to predose on D1 | C1D1 | C1D2(±1h) | C1D3(±2h) | C1D8(±8h) | C1D15(±12h) | CnD1 (±3 days) | End of treatment (±7 days) | 30, 90 days (±7 days) after the last dose | Every 3 months (±7 days) |
| **Standard Procedures** | | | | | | | | | | |
| Informed consent form[1] | x | | | | | | | | | |
| Inclusion/exclusion criteria | x | | | | | | | | | |
| Demographics/medical history/prior medications[2] | x | | | | | | | | | |
| Vital signs[3] | x | x | x | x | x | x | x | x | x | |
| Weight/height[4] | x | x | | | | | x | | | |
| Physical examination[5] | x | x | x | x | x | x | x | x | x | |
| ECOG score | x | | | | | | x | x | x | |
| 12-lead ECG[6] | x | x | x | x | x | x | x | x | | |
| Pulmonary function test[6] | x | | | | | | | | | |
| Hematology/biochemistry/urinalysis[7] | x | x | | | x | x | x | x | x | |
| Coagulation test[8] | x | | | | | x | x | x | x | |
| Pregnancy test[9] | x | | | | | | x | x | x | |
| Thyroid function test[10] | x | | | | | x | x | x | x | |
| Autoantibody[11] | x | | | | | | | | | |

| Study phase | Screening period | Treatment Period | | | | | | End of treatment visit[23] | Safety follow-up[24] | Survival follow-up[25] |
|---|---|---|---|---|---|---|---|---|---|---|
| | | Cycle 1 (21 days) | | | | | Cycle 2 and above (21 days/cycle) | | | |
| Study day | From -D28 to predose on D1 | C1D1 | C1D2(± 1h) | C1D3(± 2h) | C1D8(± 8h) | C1D15(± 12h) | CnD1 (±3 days) | End of treatment (±7 days) | 30, 90 days (±7 days) after the last dose | Every 3 months (±7 days) |
| Immunogenicity (ADA, NAb) [12] | | x | | | | | x | x | x | |
| HIV, HBV and HCV [13] | x | | | | | | | | | |
| PK [14] | | x | x | x | x | x | x | x | x | |
| Pharmacodynamics, immune cell subtypes[14] | | x | x | | x | | x | x | x | |
| Cytokines[14] | | x | x | | | | | | | |
| Adverse events[15] | x | x | x | x | x | x | x | x | | |
| Concomitant Medications | x | x | x | x | x | x | x | x | | |
| Survival | | | | | | | x | | | |
| Follow-up anti-tumor therapy | | | | | | | | x | x | x |
| Efficacy Evaluation | | | | | | | | | | |
| CT or MRI examination [16] | x | | | | | | x | x | x | |
| FDG-PET examination [17] | x | | | | | | | | | |
| Bone marrow aspiration biopsy[18] | x | | | | | | | | | |
| International Prognostic Index (IPI) score | x | | | | | | | | | |
| B symptom assessment [19] | x | x | | | | | | x | | |
| Investigational Product Infusion | | | | | | | | | | |
| JS004[20] | | x | | | | | x | | | |
| Toripalimab[20] | | x | | | | | x | | | |

| Study phase | Screening period | Treatment Period | | | | | | End of treatment visit[23] | Safety follow-up[24] | Survival follow-up[25] |
|---|---|---|---|---|---|---|---|---|---|---|
| | | Cycle 1 (21 days) | | | | | Cycle 2 and above (21 days/cycle) | | | |
| Study day | From -D28 to predose on D1 | C1D1 | C1D2(±1h) | C1D3(±2h) | C1D8(±8h) | C1D15(±12h) | CnD1 (±3 days) | End of treatment (±7 days) | 30, 90 days (±7 days) after the last dose | Every 3 months (±7 days) |
| Biomarkers | | | | | | | | | | |
| Archived or fresh tissue samples[21] | x | | | | | | | | | |
| Blood sampling for biomarkers[22] | | x | | | | | x | x | | |

**AE: adverse events; C: cycle; D: day; ECOG: Eastern Cooperative Oncology Group; ADA: anti-drug antibody**

1. Subjects should sign the informed consent form (ICF) prior to initiation of any study procedures listed in the protocol. In this study, rescreening is allowed only once for subjects who have previously failed screening.
2. Medical history/prior medications include prior anti-tumor therapies, such as chemotherapy, radiotherapy, surgery, adjuvant therapy, molecular targeted drugs (± chemotherapy), immune checkpoint inhibitors (such as anti-CTLA-4 antibody, anti-PD-1/PD-L1 antibody). The exact time of the last dose of other anti-tumor therapy before the first dose of JS004 must be recorded.
3. Vital signs, including body temperature, pulse, respiratory rate and blood pressure, should be measured after at least 5 min of comfortable rest (the start and end times of rest should be recorded). Vital signs will be measured at screening, within 1 h before the first infusion on Cycle 1 Day 1, every 15 min (±5 min) during infusion, at the first end of infusion (EOI) (+5 min), 1 h (±10 min) after the EOI, on Days 2, 3, 8, and 15 of the DLT observation period, and as necessary. For subsequent cycles (Day 1 only), vital signs will be measured within 1 h before infusion, every 15 min (±5 min) during infusion, EOI (+5 min), at the end of treatment (±7 days), at the safety follow-up visit, and as clinically indicated. Specific requirements for pulse and blood pressure monitoring are detailed in Section 7.1.3.
4. Height will only be measured once at screening. Body weight must be measured and recorded at screening and within 3 days before each dose, and the dose will be calculated according to the current body weight. No dose adjustment is required for fixed-dose groups based on body weight.
5. A complete physical examination will be performed by the investigator at screening and the end of treatment. A brief physical examination may be performed at other visits to assess discomfort and symptoms.
6. A 12-lead ECG will be performed at screening, within 1 h on Day 1 before infusion, within 1 h after EOI, and on Days 2, 3, 8, and 15 of Cycle 1. For each subsequent infusion, a 12-lead ECG will be performed within 1 h before infusion, within 1 h after EOI, at the end of treatment (±7 days), and as clinically indicated. Test items include heart rate, PR, QRS, QT, and QTc (Fridericia's formula) intervals. When ECG and PK sample collection are scheduled at the same timepoint, ECG should be performed before PK sampling. Subjects with obstructive pulmonary disease must have a baseline pulmonary function test. If the percentage of FEV1 is ≥50% of predicted, the subjects may be enrolled in the study, and other subjects may have a pulmonary function test as clinically indicated.

7. Complete blood count (CBC) includes red blood cells, hemoglobin, platelets, white blood cells, absolute neutrophils, lymphocytes, monocytes, eosinophils, and basophils. Biochemistry includes total bilirubin (TBIL), direct bilirubin (dBIL), alanine aminotransferase (ALT), aspartate aminotransferase (AST), gamma-glutamyltranspeptidase (γ-GT), alkaline phosphatase (ALP), albumin (ALB), total protein (TP), lactate dehydrogenase (LDH), blood urea nitrogen (BUN), creatinine (Cr), uric acid, potassium ($K^+$), sodium ($Na^+$), chloride ($Cl^-$), calcium ($Ca^{2+}$), magnesium ($Mg^{2+}$), phosphorus or phosphate, amylase, lipase and blood glucose, etc. Urine analysis (UA) is acceptable for specific gravity, pH, glucose, protein, ketones, and blood cells (including red blood cells and white blood cells), and microscopy will be performed as clinically indicated. If protein is ≥2+ in urine analysis, urine samples will be collected to calculate urine protein/creatinine ratio (UP/CR) or 24-hour urine will be collected. Subjects with 24-hour urine protein ≤2 g can be included in the study. These items will be tested at screening, within 7 days before the first dose of Cycle 1, on Days 8 and 15 of the DLT period, within 72 h before Cycle 2 Day 1, on Day 1 of each subsequent cycle, at the end of treatment (±7 days), at the safety follow-up visit, and as clinically indicated. If the screening assessment is performed within 72 h before the Cycle 1 Day 1 assessment, no re-test is required

8. Coagulation function test, including thrombin time (TT), prothrombin time (PT), activated partial thromboplastin time (aPTT) and international normalized ratio (INR), should be performed within 7 days before Cycle 1 Day 1, on Day 15 during the DLT period, within 72 h before Cycle 2 Day 1, on Day 1 of each subsequent cycle, at the end of treatment (±7 days), 90 days after the end of treatment (±7 days), and as clinically indicated.

9. Women of childbearing potential should have a serum or urine pregnancy test (with a sensitivity of at least 25 mIU/mL and performed in a certified laboratory) within 7 days before the first dose. Serum or urine pregnancy test will be repeated within 72 h before each subsequent dose, at the end of treatment (±7 days), during the follow-up before the safety follow-up visit as clinically indicated, and when a menstrual cycle is missed or a possible pregnancy is suspected. A serum pregnancy test should be performed if a urine pregnancy test is inconclusive. Conclusions should be based on serum pregnancy tests. Subjects with a confirmed positive pregnancy test should not be administered.

10. Thyroid function test includes tri-iodothyronine free (FT3), free T4 (FT4) and thyroid stimulating hormone (TSH), etc. Thyroid function test will be performed at screening, Day 15 (±1 day) of the DLT observation period, within 7 days before the dose of the investigational product from Cycle 2, at the end of treatment (±7 days), at the safety follow-up visit, and as clinically indicated. Other thyroid function markers may be considered if necessary.

11. Autoantibody test includes antinuclear antibodies, anti-double-stranded DNA (dsDNA) antibodies, anti-thyroglobulin antibodies. It will be performed at screening, and as clinically indicated.

12. Blood samples for immunogenicity analysis will be collected within 1 h before JS004 or toripalimab infusion in Cycles 1, 2, 4, 6, 8, 12, 16 and every 8 cycles thereafter, at the end of treatment (±7 days), and at the safety follow-up visit. If JS004 or toripalimab infusion reaction occurs in any subject, blood samples for immunogenicity analysis should be collected at the start of the event (+5 min), resolution of the reaction (+5 min), and 30 days (±3 days) after the reaction.

13. Including hepatitis B virus (HBV), hepatitis C virus (HCV), and human immunodeficiency virus (HIV) antibody test. Hepatitis B test includes hepatitis B surface antigen (HBsAg), hepatitis B surface antibody (HBsAb), hepatitis B core antibody (HBcAb), hepatitis B e antigen (HBeAg), hepatitis B e antibody (HBeAb). If HBsAg and/or HBcAb are positive, HBV DNA test should be performed further. If the anti-HCV antibody is positive for the subject, HCV RNA test is required.

14. The test timepoints for PK/PD/cytokine/immune cell subtypes are detailed in the sampling schedule for PK/PD/cytokine/immune cell subtype studies (Table 2).
15. Laboratory tests and AEs will be assessed according to NCI CTCAE v5.0. Definitions, records, causal relationship, severity, reporting cutoff date, and handling methods of AEs and SAEs are detailed in Section 8.
16. Tumor assessments will be performed per Lugano 2014 Criteria. Tumor imaging examination methods include enhanced CT or MRI or other special examinations of the chest, abdomen, pelvic cavity and any other known lesion site (e.g., neck). Baseline assessments, including cranial examination, should be performed within 28 days before enrollment. The imaging methods used to examine the same lesion during the trial should be the same as at baseline. Tumor imaging assessments will be performed every 9 weeks (±7 days) after the first dose of the investigational product until any of the following occurs: initiation of new anti-tumor therapy, progressive disease, withdrawal of consent, study discontinuation, loss to follow-up or death. Imaging examinations should be repeated for confirmation for subjects who experience the first radiographic progressive disease and meet the requirements of Section 7.3 within 4-8 weeks (±7 days). Imaging assessments should be performed for subjects who need to discontinue treatment for reasons other than progressive disease at the end of treatment (±7 days) and every 9 weeks (±7 days) thereafter until any of the following occurs: initiation of a new anti-tumor therapy, withdrawal of consent, study discontinuation, loss to follow-up or death.
17. FDG-PET examination: Baseline FDG-PET examination is required within 28 days before enrollment. During treatment, FDG-PET examination should be performed within 4 weeks when complete response (CR) is assessed by imaging such as CT or MRI.
18. Bone marrow aspirate biopsy: All subjects, except for Hodgkin's lymphoma, are required to have a baseline bone marrow aspirate biopsy, but the investigational product may be given before the bone marrow biopsy results are available. For subjects with confirmed bone marrow involvement at baseline, a repeat bone marrow aspiration biopsy (only FDG-PET confirmation is needed for Hodgkin lymphoma) is required to confirm radiographic CR during the trial within 2 weeks of completion of imaging. Bone marrow assessment is not required after a negative bone marrow aspirate biopsy.
19. B symptom assessment: B symptoms, including unexplained fever >38°C, night sweats, and weight decrease >10% within 6 months before assessment, will be assessed on Day 1 of each cycle until discontinuation of study treatment. In addition, assessments should also be performed when the subject first achieves PR or CR; If a subject achieves PR prior to CR, re-assessment is required at the time of CR.
20. JS004 monotherapy, or JS004combined with toripalimab. The duration of the first infusion of JS004 in the JS004 monotherapy regimen is at least 60 min. Without an infusion-related reaction (IRR), subsequent doses may be infused in a shortened period of at least 30 min. Cycle 1 is a DLT observation period (21 days). The infusion will be given every 3 weeks from Cycle 2 for up to 24 months until PD, loss to follow-up or death, intolerable toxicity, withdrawal of consent, or other reason for discontinuation, whichever occurs first. For subjects with the first occurrence of clinically stable PD, if the protocol requirements are met (see 7.3), the treatment may be continued by the investigator until the total duration of treatment reaches 24 months or until further PD, loss to follow-up or death, intolerable toxicity, withdrawal of consent, or other reasons for discontinuation as specified in the protocol, whichever occurs first. JS004 in combination with toripalimab is administered by intravenous infusion once every 3 weeks. JS004 is administered first, followed by toripalimab at an interval of approximately 1 h. JS004 is administered with the same regimen as that of JS004 monotherapy. The first intravenous infusion of toripalimab should be at least 60 min. If the infusion of the first dose is well tolerated, the second dose may be infused in a shortened period of at least 30 min. All subsequent infusions can be administered over at least 30 min if the infusion over 30 min is well tolerated.

21. Subjects are required to provide an archived (within 2 years) or fresh tumor tissue sample at screening for biomarker evaluation. See Section 3.12 for sample requirements.

22. In Cycle 1, blood samples (about 6 mL) will be collected in anticoagulant vacutainers for biomarker evaluation before JS004 infusion, at each imaging assessment and at the end of treatment (±7 days). Two milliliters of blood will be collected before the first dose of JS004 in Cycle 1 for tumor tissue paired blood WES test. See Section 3.12 for details.

23. End of treatment visit: If tumor CT or MRI examination is completed within 4 weeks before the decision to end the treatment, and relevant examinations for safety assessment and B symptom assessment are completed within 7 days before the decision, there is no need to repeat these examinations.

24. Safety follow-up: The safety follow-up will be ended 90 days after the last dose of the investigational product or the initiation of a new anti-tumor therapy, whichever occurs first. Subjects should return to the site for the protocol-specified safety follow-up visit 30 days (±7 days) after the last dose of the study drug or before initiating a new anti-tumor therapy. This visit does not have to be repeated if it is within the window of the end of treatment visit. Severe delayed immune-related adverse events will be monitored for 30 to 90 days after the last dose of JS004 or JS004 in combination with toripalimab. If a delayed immune-related adverse event is suspected, the subject will be asked to visit the hospital within 5 calendar days after being informed. The safety information, including AE outcome, new AE/SAE, AE of special interest and concomitant medications, will be collected through telephone follow-up at 60 days (±7 days) after the last dose of JS004 or JS004 in combination with toripalimab. Subjects should return to the hospital for all safety examinations at 90 days (±7 days) after the last dose of JS004 or JS004 in combination with toripalimab as far as possible. For those who fail to return to the hospital, the safety information, including AE outcome, new AE/SAE, AE of special interest, concomitant medications, etc., should be collected through telephone follow-up. Unresolved adverse events at the time of visit should be monitored at least every 4 weeks until resolution to Grade 0 or 1, stabilization, or withdrawal of consent, whichever occurs first.

25. Survival follow-up: The survival follow-up will be performed every 3 months (±7 days) after the last dose for survival status and subsequent anti-tumor therapy information. Telephone follow-up is acceptable.

**Table 2 Sampling Schedule for PK/PD/Cytokine/Immune Cell Subtype Studies**

| Reporting period | Screening period | Treatment Period | | | | | | | | | | | | End-of-treatment visit | Safety follow-up |
|---|---|---|---|---|---|---|---|---|---|---|---|---|---|---|---|
| | | Cycle 1 (21 days) [2] | | | | | | | | | Cycle 2 and above (21 days/cycle) | | | | |
| Study day | -28 to -1 | 1 | | | | | 2 | 3 | 8 | 15 | Day 1 of each cycle (±3 days) | | | End of treatment (±7 days) | 30, 90 days after the end of treatment (±7 days) |
| Sampling time [1] | | -1h | During infusion | Immediately after the end of infusion | 1 h after the end of infusion | 6 h after the start of infusion | 24 h | 48 h | 168 h | 336 h | -1h | During infusion | Immediately after the end of infusion | | |
| Sampling time window | | | | + 5 min | ± 5 min | ± 15 min | ±1 h | ±2 h | ±8h | ±12 h | | | +5 min | | |
| JS004[2] | | | | | | | | | | | | | | | |
| PK[3] | | x | | x | x | x | x | x | x | x | x | | x | x | x |
| PK[4] | | x | | | | | | | | | x | | | x | x |
| PD-RO[5] | | x | | | | | x | | x | | x | | | x | x |
| Cytokine[6] | | x | | | | | x | | | | | | | | |
| Immune cell subtype[7] | | x | | | | | x | | x | | x | | | x | x |
| Toripalimab [8] | | | | | | | | | | | | | | | |
| PK[9] | | x | | | | | | | | | x | | | x | x |

1. Blood should be collected from the opposite arm to which the investigational product is infused.
2. If the dose is delayed on Cycle 2 Day 1 due to an AE or other reasons, an additional sample will be collected at 504 ± 24 h in Cycle 1 (i.e., Day 22).
3. Dose escalation and dose expansion phases in Part A and Part B: In Cycle 2, PK sampling should be performed within 1 h before and immediately after JS004 infusion (+5 min); No PK sampling is required for Cycle 3. In Cycle 4, PK sampling should be performed (intensive sampling, for a total of 9 timepoints) within 1 h before and immediately after infusion (+5 min), 1 h (±5 min) after the end of infusion, and 6 h (±15 min), 24 h (±1 h), 48 h (±2 h), 168 h ± 8 h (Day 8), 336 h ± 12 h (Day 15), and 504 h ± 24 h (Day 22, i.e., within 1 h before infusion in Cycle 5) after the start of infusion. In Cycles 6, 8, 12 and 16, sampling timepoints will be within 1 h

before and immediately after JS004 infusion (+5 min). Blood samples will be collected within 1 h before administration, at the end of treatment (±7 days), and at the safety follow-up visit every 8 cycles thereafter.

4. Indication expansion phase in Part A and Part B: Blood samples will be collected within 1 h before JS004 infusion in Cycles 1, 2, 4, 6, 8, 12, 16, and every 8 cycles thereafter, at the end of treatment and the safety follow-up.

5. It is applicable to dose escalation and dose expansion phases in Part A and Part B. In Cycle 1, 3 sampling timepoints are within 1 h before JS004 infusion, 24 h ± 1 h and 168 h ± 8 h after the start of infusion (Day 8). In Cycles 2, 4, 6 and 8, blood samples will be collected within 1 h before infusion. Blood samples will be collected at the end of treatment (±7 days) and the safety follow-up.

6. Applicable to dose escalation and dose expansion phases in Part A and Part B, sampling timepoints are within 1 h before the first infusion of JS004 and 24 h ± 1 h after the start of infusion.

7. It is applicable to dose escalation and dose expansion phases in Part A and Part B. In Cycle 1, 3 sampling timepoints are within 1 h before JS004 infusion, 24 h ± 1 h and 168 h ± 8 h after the start of infusion (Day 8). In Cycles 2, 4, 6 and 8, blood samples will be collected within 1 h before infusion. Blood samples will be collected at the end of treatment (±7 days) and the safety follow-up visit.

8. Blood sampling for toripalimab PK only applies to Part B JS004 in combination with toripalimab group;

9. It is applicable to Part B. Two milliliters of blood samples will be collected within 1 h before administration of JS004 in Cycles 1, 2, 4, 6, 8, 12, 16, every 8 cycles thereafter, at the end of the treatment visit (±7 days), and 90 days after the last treatment (±7 days, as needed) to detect the blood concentration of toripalimab.

**Table 3 Sampling Schedule for Vital Signs/ECG**

| Study day | Collection time | Vital signs[1] | ECG[3] |
|---|---|---|---|
| C1D1 | 0 (within 1 h before administration) | X | X |
| | Every 15 min ±5 mm during infusion | X | |
| | EOI immediately after the end of dose (within 5 min) | X | |
| | EOI immediately after the end of dose (within 1 h) | | X |
| | 1 h ± 10 min after the end of dose | X[4] | |
| C1D2 | 24 h ± 1 h after the end of dose | X[4] | X[4] |
| C1D3 | 48 h ± 2 h after the end of dose | X[4] | X[4] |
| C1D8 | 168 h ± 8 h after the end of dose | X[4] | X[4] |
| C1D15 | 336 h ± 12 h after the end of dose | X[4] | X[4] |
| C2D1[2] | 0 (within 1 h before administration) | X | X |
| | Every 15 min ±5 mm during infusion | X | |
| | EOI immediately after the end of dose (within 5 min) | X | |
| | EOI immediately after the end of dose (within 1 h) | | X |
| End-of-treatment visit | 0 | X | X |
| 30/90 days after the last dose | 0 | X | |

Remarks:

1. Vital signs will be measured and recorded after the subject has rested comfortably for at least 5 minutes; Blood pressure will be measured using a validated automated blood pressure monitor. If possible, measurements from the same arm should be recorded using the same type of blood pressure recorder.
2. In subsequent treatment cycles, vital signs and ECGs should be measured as in Cycle 2, and as clinically indicated.
3. All ECGs should be performed after the subject has rested in a quiet supine position for at least 10 min. The examination contents include at least heart rate, QT, QTc (Fridericia's formula) and P-R interval. When the ECG assessment coincides with the pharmacokinetic (PK) blood draw simultaneously, it is preferable to perform the ECG assessment before collecting the blood sample.
4. Only applicable to the subjects in dose escalation and dose expansion phases in Part A and Part B.

# 1. Introduction: Study Background and Scientific Rationale

## 1.1. Study Background

Based on GLOBOCAN 2018 [1] data, the incidence and mortality rates of non-Hodgkin's lymphoma (NHL) in China were similar to the global average. However, the incidence and mortality rates of Hodgkin's lymphoma (HL) were lower than the global average. The treatment of malignant lymphoma includes surgery, radiotherapy, chemotherapy, targeted therapy and immunotherapy. In May 2016, the FDA approved Nivolumab, a PD-1 antibody developed by BMS, as a treatment for classical Hodgkin lymphoma (cHL) in patients who have experienced failure of autologous stem cell transplantation (ASCT) and post-transplant Brentuximab Vedotin treatment. This milestone marked the initiation of the era of immune checkpoint inhibitors (ICIs) in lymphoma treatment. However, ICIs for lymphoma are still limited to two indications, classical Hodgkin lymphoma (cHL) and primary mediastinal B-cell lymphoma (PMBL), with similar biological behavior. The PD-1 antibody approved in China is only used in third and further lines of therapy for relapsed/refractory cHL. On the other hand, not all patients receiving anti-PD-1 therapy will benefit. Overall, about 30% of patients will develop primary or secondary drug resistance, and there is still a problem that no drug is available for patients who fail anti-PD-1 therapy.

NHL, accounting for the vast majority of lymphoma (about 85-90%), is still mainly treated with traditional therapy. However, the limited response and significant toxicity of traditional chemoradiotherapy limit further benefits to the patients. Lymphoma is a highly heterogeneous malignant tumor with multiple gene mutations or abnormal expression. Various targeted therapeutic drugs, including antibody-drug conjugates (ADCs), have shown an excellent prospect in treating malignant lymphoma. However, domestic studies on targeted therapy are relatively lagging, and many targeted drugs still need a long time to be marketed in China. The limited response and significant toxicity of traditional chemoradiotherapy limit further benefits to the patients. Therefore, Chinese patients with malignant lymphoma still urgently need more treatment options. Developing new drugs targeting new immunotherapy targets and the clinical studies of montherapy or combination with PD-1 antibody drugs are also important trends in treating malignant lymphoma [2].

BTLA was identified as a member of the CD28 receptor family in 2003 [3]. It has a single IgV extracellular domain with a sequence similar to other CD28 family molecules, such as PD-1 and CTLA-4. BTLA is expressed on T and B lymphocytes as well as dendritic cell (DC) subsets. HVEM is a tumor necrosis factor (TNF) receptor widely expressed in the hematopoietic system and identified as a BTLA ligand [4]. It is expressed in the following cells: T cells, B cells, NK cells, myeloid cells, dendritic cells, as well as various tumor cells, including non-small cell lung cancer (NSCLC), melanoma, colorectal cancer and lymphoma [5-9]. The expression of HVEM in tumors is associated with poor prognosis and immune escape [5,7].

BTLA contains two immunoreceptor tyrosine-based inhibitory motif (ITIM) domains in its cytoplasmic region, which recruit SH2 domain-containing protein tyrosine phosphatases (SHP-1 and SHP-2) upon receptor activation by monoclonal antibody (mAb) crosslinking or ligand binding. BTLA-deficient mice are viable and fertile, but exhibit enhanced T cell activation and disease aggravation in autoimmune and inflammatory animal models, including experimental autoimmune encephalomyelitis (EAE), systemic lupus erythematosus (SLE), allergic airway inflammation, and Con-A-induced hepatitis [3], indicating an inhibitory effect of BTLA on T cell activation in vivo. Studies on peripheral blood mononuclear cells (PBMCs) in patients with melanoma and NSCLC have shown [10,11] that BTLA is highly expressed on tumor-specific cytotoxic T lymphocytes (CTLs) and inhibits T cell function in response to tumor-expressed HVEM, suggesting that blocking BTLA improves T cell function and anti-tumor immunity.

BTLA is also co-expressed with PD-1 on tumor-specific CTLs in patients with melanoma and NSCLC [11,12]. Importantly, co-blockade of the BTLA and PD-1 pathways may increase the number of melanoma-specific CTLs and the production of effector cytokines, whereas blockade of BTLA or PD-1 alone has a limited effect, suggesting that the BTLA pathway is a potential resistance mechanism in patients with anti-PD-1 refractory melanoma [12]. In addition, in vitro and in vivo studies have shown that anti-BTLA agents can promote tumor-specific T cell-mediated immune responses, reduce tumor burden and improve survival in a variety of tumor models [13].

JS004, developed by Shanghai Junshi Biosciences Co., Ltd., is intended for the treatment of patients with relapsed/refractory malignant lymphoma. JS004 is a fully humanized antibody that binds to BTLA. It blocks negative regulatory signals and enhances T-cell activation to improve anti-tumor responses. Preclinical studies have shown that JS004 blocks the interaction between HVEM and BTLA on the cell surface and the downstream signal transduction of BTLA. JS004 can effectively inhibit MC38-hBTLA mouse colon cancer in human BTLA transgenic mice. These results demonstrate the potential benefit of JS004 in the treatment of cancer.

#### 1.1.1. Drug Name

Generic name: JS004 for injection

English name: JS004 for Injection

Chinese pinyin: Zhusheye JS004

Trade name: pending

Study code: JS004

### 1.1.2. Pharmacological Type and Mechanism of Action

JS004 is a humanized IgG4 κmAb targeting BTLA. It consists of two identical light chains (219 amino acids) and two identical heavy chains (446 amino acids) linked by inter-chain disulfide bonds. An S-P mutation was introduced at site 227 (S227P) in the heavy chain hinge region to improve molecular stability. JS004 also contained an N-linked glycosylation site at heavy chain amino acid 296 (N296). The deglycosylated molecular weight of JS004 was 145.7 kD.

BTLA was expressed on T and B lymphocytes and could inhibit T cell proliferation and cytokine production upon activation by its ligand HVEM [4,14]. JS004 specifically and effectively blocked the interaction between HVEM and BTLA and released an inhibitory effect on T cell-mediated immune responses. BTLA-deficient mice were healthy and fertile, with no obvious defects under normal conditions. However, in autoimmune and inflammatory animal models, BTLA knockout mice exhibited enhanced T cell activation and progressive diseases, including EAE, SLE, asthmatic airway inflammation and Con-A-induced hepatitis, suggesting an inhibitory effect of BTLA on T cell activation in vivo [3,15]. PBMC studies in melanoma patients showed that BTLA could be highly expressed on tumor-specific CTLs and inhibit T cell function after the involvement of tumor-expressed HVEM [16]. These results showed that BTLA blockade could improve T cell function and anti-tumor immune function.

### 1.1.3. Pharmacodynamic Study

Nonclinical pharmacological studies of JS004 aimed to assess and explore the pharmacological effects of JS004 at the cellular level in transgenic mice. In addition, the PK/TK and toxicology studies of JS004 were evaluated in the related species cynomolgus monkeys.

#### 1.1.3.1 In Vitro Pharmacology

In vitro pharmacological studies showed that JS004 targeted BTLA with high specificity and affinity and could effectively block the interaction between BTLA and its natural ligand HVEM. JS004 could bind to human and cynomolgus monkey BTLA with similar affinity but not to rat or mouse BTLA. JS004 bound BTLA on the surface of cynomolgus monkey and human PBMC cells with EC50 values ranging from 26.9 to 71.84 ng/mL and 16.64 to 29.26 ng/mL, respectively. The RO study accompanied by the five-week long-term toxicity study showed that JS004 could bind to BTLA on the surface of cynomolgus monkey cells in vivo, and the receptor was almost completely occupied. The complete receptor occupancy rate in all treatment groups lasted until the end of the recovery period (Day 63) without dose-dependence. In the Jurkat reporter gene system, the mechanism of action of JS004 was to alleviate the negative regulatory signal of BTLA and promote downstream NFAT signal transduction, with an EC50 of 0.1057 µg/mL. A stronger NFAT signal could be detected when JS004 and anti-PD-1 antibody (toripalimab/JS001) were administered in combination, suggesting that blocking both BTLA and PD-1 pathways had additive or synergistic effects in promoting T cell activity.

Studies assessing the Fc function of JS004 indicated that JS004 bond weakly to FcγRIIIA (CD16a) V176, FcγRIIA (CD32a), R167, FcγRI (CD64) and FcRn, but had no binding activity to C1q and did not mediate ADCC or CDC.

Detailed results are provided in the Investigator's Brochure.

#### 1.1.3.2 In Vivo Pharmacology

To assess the in vivo anti-tumor activity of JS004, MC38-hHVEM mouse colon cancer (overexpressing human HVEM) in a human BTLA transgenic mouse model was used (Table 4). Although there was no obvious dose effect, JS004 showed significant anti-tumor effects at a dose of 3 mg/kg, with a TGI value of 56.4% (Table 5 and Figure 1).

**Table 4 Effect of JS004 on MC38-hBTLA Mouse Colon Cancer in Human BTLA Transgenic Mice**

| Study Type | Route of Administration | Species | Dosage and Administration | Results of Interest |
|---|---|---|---|---|
| Inhibition of MC38-hHVEM tumor growth in hBTLA transgenic mice | *i.p.* | BALB/c mice<br>HBTLA transgenic mice | JS004 (0, 1, 3, or 10 mg/kg)<br>Anti-KLH Ab (10 mg/kg) or 0.9% saline (BIW×7) | JS004 at 1, 3 or 10 mg/kg induced anti-tumor activity against subcutaneous MC38-hHVEM tumors. Compared with anti-KLH Ab (10 mg/kg), the TGI values of JS004 at 1, 3, and 10 mg/kg were 33.7%, 56.4%, and 48.0%, respectively. JS004 at 3 mg/kg produced significant tumor inhibition ($p<0.05$). |

*i.p.*: Intraperitoneal injection.

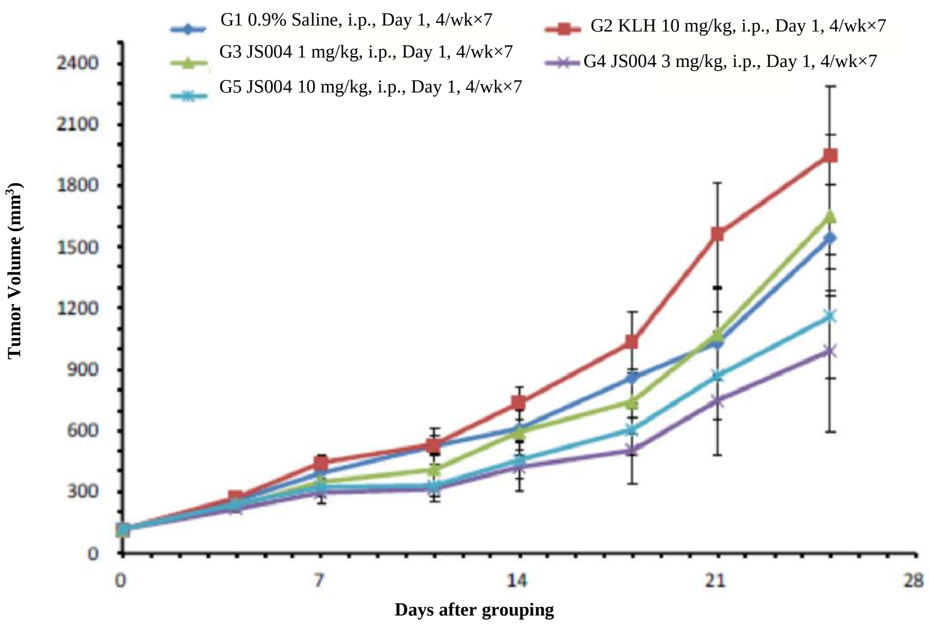


**Figure 1 Effect of JS004 on MC38-hBTLA Mouse Colon Cancer Tumor Progression in Human BTLA Transgenic Mice**

**Table 5 Effect of JS004 on MC38-hBTLA Mouse Colon Cancer Tumor Volume in Human BTLA Transgenic Mice**

| Treatment Group | Study Drug | Tumor Volume ($mm^3$) [a] | | | $P^b$ |
|---|---|---|---|---|---|
| | | Before administration | 21 days after the first dose | TGI (%) | |
| 2 | KLH (10 mg/kg) | 118±2 | 1560±256 | | |
| 3 | JS004 (1 mg/kg) | 118±3 | 1073±224 | 33.7 | 0.175 |
| 4 | JS004 (3 mg/kg) | 118±2 | 747±268 | 56.4 | 0.046 |
| 5 | JS004 (10 mg/kg) | 118±3 | 868±211 | 48.0 | 0.056 |

Note: JS004 = JS004; [a]: mean ± SD; [b]: Statistical analysis of tumor volume (by t-test) for JS004-treated group versus anti-KLH antibody-treated group. TGI (%) = [1-(Ti-T0)/(Vi-V0)]×100, where Ti is the mean tumor volume for treatment group on Day i; T0 is the mean tumor volume for treatment group on Day 0; Vi is the mean tumor volume for saline group on Day i; V0 is the mean tumor volume for the saline group on Day 0.

### 1.1.4. Toxicology Study

Based on the "Guidelines for Non-clinical Safety Evaluation of Therapeutic Biological Products", cynomolgus monkeys were selected as the main test animals in toxicology studies. Preclinical safety and toxicology study results were systematically evaluated in strict compliance with GLP requirements, including single-dose toxicity, repeat-dose toxicity, hemolysis, local irritation, and other toxicity studies (immunotoxicity and immunogenicity, cytokine release (GLP)). See Table 6 for the summary of the results.

**Table 6 Summary of Toxicity Studies**

| Study Type | Species and Strain | Method of Administration | Dosing Period | Dose (mg/kg) | GLP Compliance |
|---|---|---|---|---|---|
| **Non-pivotal** | | | | | |
| Single-dose toxicity | Monkey/cynomolgus monkey | *i.v.* | 1 day | JS004: 1, 200 (single dose) | Non-GLP |
| Single-dose toxicity | Monkey/cynomolgus monkey | *i.v.* | 1 day | JS004 and TAB001: 1, 10, 100 each (single dose) | Non-GLP |
| Single-dose toxicity | Monkey/cynomolgus monkey | *i.v.* | 1 day | JS004: 0, 213 (single dose) | GLP |
| **Pivotal** | | | | | |
| Repeat-dose toxicity | Monkey/cynomolgus monkey | *i.v.* | 5 weeks | JS004: 0, 10, 30, 100 g/kg; (Q1W×6) | GLP |
| Tissue Cross-reactivity Study | Human/tissue | *In Vitro* | NA | 1, 5 μg/mL | Non-GLP |
| Cytokine release | Human | *In Vitro* | NA | 10 ng/mL - 1 mg/mL | Non-GLP |

NA = not applicable; i.v. = intravenous administration. Note that the highest NOAEL is underlined for single- and repeat-dose systemic toxicities unless otherwise specified.

### 1.1.4.1 Single-dose Studies

Key findings from the single-dose toxicity studies are summarized in Table 7. The results showed that a single intravenous injection of JS004 in cynomolgus monkeys was well tolerated at the maximum administered dose (MFD) of 213 mg/kg. The NOAEL under the test conditions was 213 mg/kg.

**Table 7 Summary of Single-dose Toxicity Studies**

| Study | Design | Method of Administration/Dosing Period/Dose (mg/kg) | MTD/NOAEL |
|---|---|---|---|
| Single-dose toxicity | Two groups of cynomolgus monkeys (1/sex/group) received JS004 at 1 or 200 mg/kg (i.v.) | • No death<br>• There were no overall findings other than transient changes in total bilirubin, red blood cell count, hemoglobin, and hematocrit that occurred 2 days after administration but were resolved on SD15 and not considered AEs.<br>• There was no significant sex difference in systemic exposure. Exposure was not dose-proportional at 200 mg/kg due to clearance saturation.<br>• On SD28, ADAs were detected in the 1 mg/kg group.<br>• The NOAEL was 200 mg/kg | NOAEL = 200 mg/kg |
| Single-dose toxicity | Three groups of cynomolgus monkeys (1/sex/group) received TAB001 and JS004 at 10, 30, or 100 mg/kg (i.v.), respectively. | • No death<br>• No overall findings<br>• There was no sex difference in systemic exposure to TAB001 or JS004 at doses ranging from 1 to 10 mg/kg. In the 100 mg/kg group, exposure to both test articles was higher in males than in females.<br>• ADAs to both test articles were detected in the 1 and 10 mg/kg groups, but not in the 100 mg/kg group. JS004 and TAB001: 1, 10, 100 each (single dose) | NOAEL = 100 mg/kg |
| Single-dose toxicity (GLP Study, MFD) | Two groups of cynomolgus monkeys (1/sex/group) received JS004 at 0 or 213 mg/kg (i.v.) | • No death<br>• No overall findings | NOAEL = 213 mg/kg |

*i.v.*: intravenous infusion, MFD: maximum feasible dose, NOAEL: no-observed-adverse-effect level

### 1.1.4.2 Repeat-dose Studies

The toxicity and TK profile of JS004 were evaluated in cynomolgus monkeys after intravenous injection of JS004 for 5 weeks (Q1W×6 doses) under GLP conditions, and the reversibility of the toxicity following a 4-week recovery period was also assessed. In this study, each group of animals (5/sex/group) received JS004 at 0, 10, 30, and 100 mg/kg once a week for a total of 6 doses. The results are summarized below:

- There were no unscheduled deaths during the study period.
- No JS004-related clinical manifestations, administration site irritation, body weight, food consumption, body temperature, cardiovascular and respiratory safety pharmacology, ophthalmology, clinical chemistry, immune function, urinalysis, fecal occult blood, bone marrow analysis, or macroscopic or microscopic findings in organ weights were found in each JS004 group at the end of the dosing period and during the recovery period. No circulating immune complex deposition was found in the kidneys of the animals.

- Changes in hematology and coagulation parameters related to JS004 administration were observed in monkeys in the 100 mg/kg group compared with the control group, including decreased RBC, HGB, HCT, and increased NEU%. However, these changes were resolved after the 4-week recovery period.
- The TK study showed no significant sex differences in exposure ($C_{max}$ and $AUC_{0\text{-}last}$). After 5 consecutive doses, there was no significant accumulation in the 10 mg/kg group, but some accumulation was observed in the 30 and 100 mg/kg groups. Anti-drug antibodies were detected in 5/10 and 1/10 animals in the 10 and 30 mg/kg groups, respectively.

Therefore, considering the severity of JS004-related changes observed in the study and the anti-tumor indication of JS004, the no-observed-adverse-effect level (NOAEL) was determined to be 30 mg/kg, with $C_{max}$ and $AUC_{0\text{-}120\ hr}$ of 1.480 mg/mL and 120.261 mg•hr/mL on Day 29, respectively. Although the highest non-severe toxic dose (HNSTD) was not defined in the study report, JS004 was well tolerated at up to 100 mg/kg without obvious adverse reactions. On Day 29 of the dosing period, the mean $C_{max}$ and AUC corresponding to this dose level were 4.266 mg/mL and 341.061 mg•hr/mL, respectively.

#### 1.1.4.3 Genotoxicity

No genotoxicity studies were conducted with JS004. As described in ICH guideline S6 (R1), the scope and type of conventional genotoxicity studies conducted in the pharmaceutical industry do not apply to biotechnology-developed pharmaceuticals as these drugs are not expected to interact with DNA or other chromosomal components.

#### 1.1.4.4 Immunotoxicity Study

Soluble and plate-bound JS004 (JS004 bound to the plate by plating 0.3-10 µg/mL anti-Fc antibody) , at concentrations ranging from 10 ng/mL to 1 mg/mL, were incubated with PBMCs from healthy donors (PBMCs from 6 donors tested), either non-activated or activated by anti-CD3 plus anti-CD28 beads. After 24 h of incubation, culture supernatants were collected and analyzed for cytokine levels. Soluble anti-CD3 antibody OKT3 (10 µg/mL) or soluble anti-CD3 antibody (50 or 250 ng/mL) plus anti-CD28 antibody (2.5 µg/mL) was used as positive controls, and nivolumab (Opdivo®), pembrolizumab (Keytruda®), or IgG4 antibody (anti-KLH humanized IgG4) was used as negative controls.

Anti-CD3 antibody, or anti-CD3 antibody plus anti-CD28 antibody strongly induced cytokine production (IL-2, IL-4, IL-6, IL-10, TNFα, and IFN-γ), while neither soluble nor plate-bound forms of JS004 over a wide range of concentrations induced massive cytokine release from non-activated and activated human PBMCs. The study showed that JS004 was similar to pembrolizumab, nivolumab and anti-KLH IgG4 antibodies, with a low possibility of inducing a cytokine release storm.

### 1.1.5. Tissue Cross-Reactivity in Human and Cynomolgus Monkeys

The cross-reactivity of JS004 with normal human tissues was investigated under non-GLP conditions by immunohistochemical staining technology to provide a reference for the clinical design of the main observation indicators and the monitoring of clinical toxicity and side effects. JS004 and negative control (anti-KLH-IgG4 antibody) were tested at concentrations of 1 and 5 μg/mL for potential cross-reactivity with cryosections of 36 normal human tissues (each from at least 3 donors).

The results showed that JS004 bound to the membrane and cytoplasm of monocytes in human lymphoid tissues, consistent with the literature report that BTLA was expressed in B cells and T cells [17]. In addition, JS004 staining was observed in the cytoplasm of smooth muscle cells in various human tissues and myofibroblasts in human testes. BTLA expression by these tissue components has not been previously reported. However, the binding of antibodies to cytoplasmic sites in tissue cross-reactivity studies is considered to be of low or no toxicological significance due to the limited ability of antibody drugs to enter the cytoplasm in vivo [18].

### 1.1.6. Safety Pharmacology

Safety pharmacology studies were conducted in strict compliance with Good Laboratory Practice (GLP) requirements to evaluate the effects of JS004 on the central nervous system of SD rats and the cardiovascular and respiratory systems of cynomolgus monkeys. Concomitant with the five-week repeated-dose toxicity study in cynomolgus monkeys, in which JS004 was administered intravenously at doses of 10, 30, 100 mg/kg to cynomolgus monkeys, no effects of JS004 on body temperature, respiration (respiratory rate and amplitude), ECG (heart rate, R wave, P wave, P-R interval, QRS interval, QT interval, ST-segment and T wave), and blood pressure (SBP, DBP, and MBP) were observed.

A single intravenous injection of 10, 30 or 100 mg/kg JS004 to SD rats had no significant effect on central nervous system function.

### 1.1.7. Pharmacokinetic Study

#### 1.1.7.1. Pharmacokinetics in Animals

The in vivo PK/TK of JS004 was evaluated in the relevant species, cynomolgus monkeys, using intravenous drip (i.v.) infusion, which was the same route of administration as in the human clinical studies. The PK profile of JS004 in cynomolgus monkeys showed that the time to peak serum concentration (Tmax) of JS004 ranged from 0.5 to 2 h. After the first dose, a linear pharmacokinetic profile was observed; however, after 5 doses, a nonlinear pharmacokinetic profile was observed. Systemic exposure parameters did not reveal significant differences in exposure between sexes. There was some accumulation in animals, especially at higher doses (i.e., 30 mg/kg and 100 mg/kg).

### 1.1.7.2. Single-Dose Pharmacokinetic Studies

Single-dose PK/TK studies are summarized in Tables 8, 9, 10, and 11.

**Table 8 Summary of Single-Dose Pharmacokinetic/Toxicokinetic Studies**

| Study Type | Route of Administration | Species | Dosage and administration | Results -|
|---|---|---|---|---|
| Single- and multiple-dose PK studies | *i.v.* | Cynomolgus monkeys (3/sex/group) | Single-dose:<br>JS004: 1, 3, 10 mg/kg<br>Repeat-dose:<br>JS004: 3 mg/kg Q1W X5 | After a single dose (1, 3 and 10 mg/kg):<br>• $T_{max}$ ranged from 0.5 h to 1 h.<br>• There were no significant sex differences in exposure AUC ($AUC_{last}$ and $AUC_{INF\_obs}$) and C max at these dose levels (p>0.05).<br>• Systemic exposure $C_{max}$ and $AUC_{last}$ or $AUC_{inf\_obs}$ increased proportionally with increasing dose from 1 to 10 mg/kg.<br>After repeated doses (3 mg/kg):<br>$T_{max}$ ranged between 0.5 and 1 h after the first and last doses, and no significant accumulation was observed as the AR value for $AUC_{0-168}$ was equal to 1.1. |
| Single-dose TK study | *i.v.* | Cynomolgus monkeys (1/sex/group) | JS004: 1, 200 mg/kg | • There were no significant sex differences in systemic exposure ($C_{max}$ and $AUC_{0-t}$).<br>• Systemic exposure was dose proportional to $C_{max}$ but not dose proportional to $AUC_{0-t}$.<br>• At the high dose (200 mg/kg), the elimination saturation of JS004 was similar.<br>• Clearance was slow and comparable between doses and sexes.<br>• Anti-JS004 antibodies were detected in all animals (1 male and 1 female) receiving 1 mg/kg, but not in animals receiving 200 mg/kg.<br>• In animals receiving JS004 at 1 mg/kg, ADAs may have affected the elimination rate. |

| Study Type | Route of Administration | Species | Dosage and administration | Results |
|---|---|---|---|---|
| Single-dose TK study | *i.v.* | Cynomolgus monkeys (1/sex/group) | JS004 + TAB001: 1, 10 and 100 mg/kg each | • At 1 and 10 mg/kg, $AUC_{0-t}$ of TAB001 and JS004 were almost identical and dose-proportional between sexes, but at 100 mg/kg, the systemic dose ($AUC_{0-t}$ ) of TAB001 and JS004 were higher in males than in females.<br>• At the dose of 100 mg/kg, TAB001 and JS004 have similar clearance saturation from serum.<br>• In 1 and 10 mg/kg dose groups, animals in both sexes showed a positive ADA response to JS004 and TAB001, but no ADA was detected at 100 mg/kg for each test article. |

*i.v.*: intravenous infusion

**Table 9 Mean Pharmacokinetic Parameters of JS004 in Cynomolgus Monkeys after a Single Intravenous Dose**

| | | Group 1 (1 mg/kg) | | | | Group 2 (3 mg/kg) | | | | Group 3 (10 mg/kg) | | | |
|---|---|---|---|---|---|---|---|---|---|---|---|---|---|
| **Variable** | **Unit** | N | **Mean** | **SD** | **CV %** | N | **Mean** | **SD** | **CV %** | N | **Mean** | **SD** | **CV %** |
| $AUC_{INF\_obs}$ | μg•hr/mL | 5 | 1950 | 527 | 27.0 | 6 | 8560 | 2620 | 30.6 | 5 | 30200 | 6180 | 20.5 |
| $AUC_{last}$ | μg•hr/mL | 6 | 1630 | 358 | 22.0 | 6 | 8070 | 2600 | 32.2 | 6 | 26200 | 8850 | 33.8 |
| $Cl_{\_obs}$ | mL/h/kg | 5 | 0.543 | 0.146 | 26.9 | 6 | 0.388 | 0.150 | 38.6 | 5 | 0.344 | 0.0831 | 24.1 |
| $C_{max}$ | μg/mL | 6 | 34.4 | 4.79 | 13.9 | 6 | 87.2 | 13.7 | 15.7 | 6 | 288 | 20.9 | 7.28 |
| $MRT_{last}$ | h | 6 | 47.9 | 10.3 | 21.6 | 6 | 116 | 58.4 | 50.4 | 6 | 106 | 35.8 | 33.9 |
| $T_{1/2z}$ | h | 6 | 51.2 | 17.1 | 33.3 | 4 | 90.4 | 34.8 | 38.5 | 6 | 65.5 | 23.8 | 36.3 |
| $T_{max\$}$ | h | 6 | 0.500 | [0.500 | 1.00] | 6 | 0.500 | [0.500 | 1.00] | 6 | 0.500 | [0.500 | 2.00] |
| $V_{ss\_obs}$ | mL/kg | 6 | 37.7 | 6.79 | 18.0 | 6 | 44.8 | 8.97 | 20.0 | 6 | 45.2 | 4.20 | 9.30 |
| $V_{z\_obs}$ | mL/kg | 6 | 37.5 | 6.37 | 17.0 | 6 | 39.4 | 8.86 | 22.5 | 6 | 36.1 | 13.3 | 36.9 |

$T_{1/2}$: elimination half-life; $AUC_{0-last}$: area under the serum concentration versus time curve from time 0 to the last measurable concentration timepoint; CL: clearance; MRT: mean residence time. Data are presented as mean values for males and females. $: Median [min, max] is provided

### Table 10 Mean Pharmacokinetic Parameters of Serum JS004 after Repeated Intravenous Doses

| | Group 4, the first dose | | | | Group 4, the last dose | | | | AR |
|---|---|---|---|---|---|---|---|---|---|
| **Variable** | **N** | **Mean** | **SD** | **CV%** | **N** | **Mean** | **SD** | **CV%** | |
| $AUC_{0\_168}$ | 6 | 5360 | 1100 | 20.5 | 5 | 5870 | 5040 | 85.9 | 1.10 |
| $AUC_{INF\_obs}$ | 1 | NC | NC | NC | 5 | 8720 | 8730 | 100 | |
| $AUC_{1ast}$ | 6 | 5170 | 1040 | 20.2 | 6 | 7260 | 8580 | 118 | |
| $Cl_{\_obs}$ | 1 | NC | NC | NC | 5 | 24.3 | 53.0 | 218 | |
| $C_{max}$ | 6 | 93.8 | 16.8 | 17.9 | 6 | 80.2 | 56.0 | 69.8 | 0.86 |
| $MRT_{1ast}$ | 6 | 58.2 | 13.0 | 22.3 | 6 | 69.1 | 75.3 | 109 | |
| $T_{1/2z}$ | 1 | NC | NC | NC | 5 | 37.0 | 32.8 | 88.6 | |
| $T_{max\$}$ | 6 | 0.500 | [0.500 | 0.500] | 6 | 0.50 | [0.500 | 1.00] | |
| $V_{ss\_obs}$ | 6 | 52.1 | 13.3 | 25.6 | 5 | 54.9 | 52.4 | 95.5 | |
| $V_{z\_obs}$ | 6 | 53.9 | 14.8 | 27.5 | 5 | 57.5 | 78.1 | 136 | |

$T_{1/2}$: elimination half-life, $AUC_{0-last}$: area under the serum concentration versus time curve from time 0 to the last measurable concentration timepoint, CL: clearance, MRT: mean residence time. Data are presented as mean values for males and females. $: Median [min, max] is provided; AR: accumulation ratio.

### Table 11 Mean Serum Toxicokinetic Parameters of JS004 after a Single Intravenous Dose

| | Group 1: 1 mg/kg | | Group 2: 200 mg/kg | |
|---|---|---|---|---|
| **Parameter** | **22371 (M)** | **22372 (F)** | **22373 (M)** | **22374 (F)** |
| $C_{max}$(ng/mL) | 26977 | 28558 | 5415161 | 4725142 |
| $C_{max}$/dose (ng•kg•$mL^{-1}$•$mg^{-1}$) | 26977 | 28558 | 27076 | 23626 |
| $t_{max}$( h) | 0.50 | 0.50 | 1.00 | 0.50 |
| $T_{1ast}$ ( h) | 240 | 240 | 648 | 648 |
| $t_{½}$(h) | 130 | 75.4 | 312 | 312 |
| $AUC_{0-t}$ (ng•h•$mL^{-1}$) | 2126568 | 1851489 | 893635311 | 738323700 |
| $V_d$ (mL•$kg^{-1}$) | 66.5 | 53.8 | 77.9 | 96.8 |
| $V_{ss}$ (mL•$kg^{-1}$) | 62.0 | 52.8 | 74.2 | 85.8 |
| Cl (mL•$h^{-1}$•$kg^{-1}$) | 0.36 | 0.49 | 0.17 | 0.22 |
| MRT ( h) | 92.5 | 84.1 | 232 | 217 |

$T_{1/2}$: elimination half-life; $AUC_{0-last}$: area under the serum concentration versus time curve from time 0 to the last measurable concentration timepoint; CL: clearance; MRT: mean residence time. Data are presented as mean values for males and females. Vd: volume of distribution; Vss: apparent volume of distribution at steady state.

### 1.1.7.3. Repeat-Dose Toxicokinetic Studies

The results of the repeat-dose toxicokinetic study are summarized in Table 12.

**Table 12 Summary of the Repeat-Dose Toxicokinetic Studies**

| Study Type | Route of Administration | Species | Dosage and administration | Results |
|---|---|---|---|---|
| 5-Week Repeat-Dose Toxicokinetic Study in Cynomolgus Monkeys by Intravenous Injection with a 4-Week Recovery Period | i.v. | Cynomolgus monkeys (5/sex/group) | Repeated-dose: JS004: 0, 10, 30, 100 mg/kg; (Q1WX 5) | • There was no significant sex difference in systemic exposure.<br>• Linear regression analysis of dose showed that mean JS004 $C_{max}$ and $AUC_{0\text{-}last}$ values increased dose-proportionally after the first dose, but not linearly after the fifth dose.<br>• Accumulation ratios ($AUC_{0\text{-}120\ hr}$ Day 29/$AUC_{0\text{-}120\ hr}$ Day 1) were generally greater than 1 over the dosing period, indicating that JS004 accumulated to some extent throughout the body, especially at higher doses (30 and 100 mg/kg).<br>• ADAs were detected in monkeys at 10 mg/kg (5/10) and 30 mg/kg (1/10), but not at 100 mg/kg. (Table 12, Figure 2) |

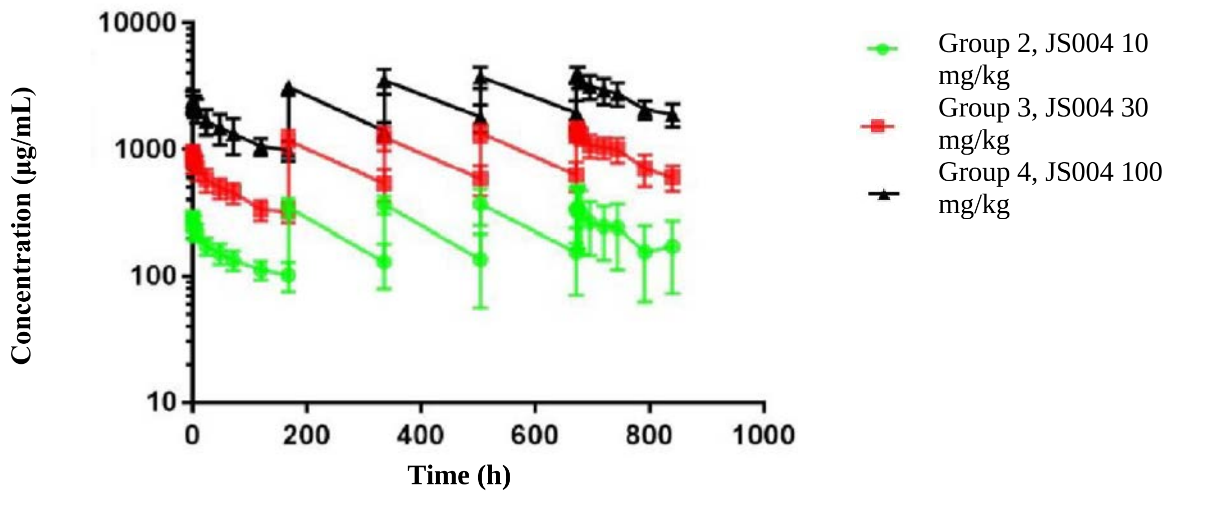


**Figure 2 Serum Concentration Profiles of JS004 in Cynomolgus Monkeys Following Repeated Doses of JS004 by Intravenous Infusion**

**Table 13 Comparison of Exposure in Female and Male Cynomolgus Monkeys after Repeated Doses of JS004**

| Study day | Dose (mg/kg) | Female $C_{max}$ | Male $C_{max}$ | Cmax ratio (F/M) | Female $AUC_{0-120\ h}$ | Male $AUC_{0-120\ h}$ | $AUC_{0-120\ h}$ ratio |
|---|---|---|---|---|---|---|---|
| D1 | 10 | 282 | 291 | 0.97 | 18282 | 18580 | 0.98 |
| | 30 | 819 | 1025 | 0.80 | 52942 | 67028 | 0.79 |
| | 100 | 2591 | 2392 | 1.08 | 186761 | 166141 | 1.12 |
| D29 | 10 | 397 | 341 | 1.16 | 28843 | 24006 | 1.20 |
| | 30 | 1352 | 1607 | 0.84 | 102892 | 137630 | 0.75 |
| | 100 | 4707 | 3824 | 1.23 | 352124 | 329998 | 1.07 |

*: P<0.05, $AUC_{0-120hr}$: area under the serum concentration versus time curve from time 0 to the measurable concentration timepoint (120 hours), $C_{max}$: maximum serum concentration.

**Table 14 Exposure and Dose Proportionality after Repeated Doses of JS004**

| | **Dose ratio*** | Dose ratio | $C_{max}$ ratio | $AUC_{0-120\ h}$ ratio |
|---|---|---|---|---|
| D1 | L/M/H | 1/3.3/10 | 1/3.2/8.7 | 1/3.3/9.6 |
| D29 | L/M/H | 1/3.3/10 | 1/4/11.6 | 1/4.6/12.9 |

*L: 10 mg/kg; M: 30 mg/kg; H: 100 mg/kg; $AUC_{0-120\ hr}$: area under the serum concentration versus time curve from time 0 to the measurable concentration timepoint (120 hours); $C_{max}$: maximum serum concentration.

**Table 15 Cumulative Exposure of JS004 (D29 vs D1)**

| Dose group | Animal No. | $C_{max}$ ratio (mean) | $AUC_{0-120\ hr}$ ratio (mean) |
|---|---|---|---|
| 10 mg/kg | 10 | 1.32 | 1.46 |
| 30 mg/kg | 10 | 1.62 | 2.03 |
| 100 mg/kg | 10 | 1.74 | 1.96 |

$AUC_{0-120\ hr}$: area under the serum concentration versus time curve from time 0 to the timepoint with a measurable concentration (120 hours); $C_{max}$: maximum serum concentration.

JS004 is an anti-BTLA monoclonal antibody of the IgG4 subtype, and no distribution, metabolism, and excretion studies were conducted as it is expected to be degraded to inactive protein substances and eventually to amino acids.

### 1.1.7.4. Pharmacokinetic Drug Interactions

No drug-drug interaction PK studies were conducted. In vivo degradation of monoclonal antibodies by biochemical pathways is expected, independent of CYP enzymes. Therefore, typical monoclonal antibodies like JS004 are not expected to interact with molecules metabolized by these enzymes.

### 1.1.8. Clinical Study Progress

Shanghai Junshi Biosciences Co., Ltd. is conducting drug research and development of JS004 worldwide, and JS004 has not yet been marketed in any country. The results of non-clinical studies and ongoing clinical studies indicate that JS004 has potential therapeutic effects on advanced malignancies. The therapeutic effect of JS004 is currently being evaluated in patients with advanced solid tumors and relapsed/refractory malignant lymphomas who have failed standard treatment or have no acceptable standard treatment.

| Study Number | Tumor Type | Number of Medicated Subjects | Study Title | Study Progress | Study Status |
|---|---|---|---|---|---|
| TAB004-001 (USA FIH) | Solid tumors and hematomas | 25 | A First-in-Human, Multicenter, Open-Label, Phase 1 Dose-Escalation and Cohort Expansion Study to Evaluate the Safety, Tolerability, and Pharmacokinetics of TAB004 in Subjects with Advanced Solid Malignancies including Lymphoma | • 0.3 mg/kg: 3 subjects (hepatic neuroendocrine tumor, colon cancer, intestinal cancer)<br>• 1 mg/kg: 3 subjects (sarcoma uterus, cecal leiomyosarcoma, lung squamous cell carcinoma)<br>• 3 mg/kg: 10 subjects (parotid gland cancer, sarcoma, 2 of melanoma, 2 of lung cancer, 2 of pancreatic cancer, 2 of intestinal carcinoma)<br>• 10 mg/kg: 9 subjects (oesophageal cancer, pancreatic carcinoma, 2 of intestinal carcinoma, oropharyngeal cancer, gastric cancer, carcinoma of tongue, lung cancer, melanoma) | The RP2D has been determined to be 3 mg/kg |
| JS004-001-I | Solid tumors | 11 | Phase I study of recombinant humanized anti-BTLA monoclonal antibody (JS004) in patients with advanced solid tumors | • 0.3 mg/kg: 3 subjects (lung adenocarcinoma, ovarian cancer, and gallbladder cancer)<br>• 1 mg/kg: 3 subjects (esophageal squamous cell carcinoma, esophageal squamous cell carcinoma, and malignant melanoma)<br>• 3 mg/kg: 4 subjects (duodenal papillary adenocarcinoma, breast cancer, and 2 of lung adenocarcinoma)<br>• 10 mg/kg, 3 subjects (lung adenocarcinoma, soft tissue sarcoma, and lung adenocarcinoma) | The RP2D is 3 mg/kg, and the study has entered the 10 mg/kg dose expansion phase |

#### 1.1.8.1.1. Study TAB004-001 (A First-in-Human, Multicenter, Open-Label, Phase 1 Dose-Escalation and Cohort Expansion Study to Evaluate the Safety, Tolerability, and Pharmacokinetics of TAB004 in Subjects with Advanced Solid Malignances including Lymphoma)

In Apr. 2019, FDA approved the application for clinical trials of JS004 in patients with advanced unresectable or metastatic solid tumors (including lymphoma) and PD-1 antibody-resistant patients. The Phase I study (NCT04137900) carried out in the US completed the treatment of the first subject on 30 Oct. 2019. As of 18 Jan. 2020, the dose escalation of 0.3 mg/kg, 1 mg/kg, 3 mg/kg and 10 mg/kg had been completed, with good tolerance and no DLT.

1) **Safety results** Safety data are available for 25 subjects who received at least 1 dose of JS004 (Table 1). Three subjects experienced adverse events leading to study drug interruption or discontinuation, 2 in the 10 mg dose group and 1 in the 0.3 mg dose group. One of these subjects experienced a study drug-related adverse event leading to dose interruption. Of these 25 subjects, 14 subjects (56%) experienced adverse events that were related to the study drug treatment at the discretion of the investigator (Table 2). Common drug-related adverse events were asthenia (24%), diarrhoea (12%), and pruritus (12%). Seven subjects (28%) experienced serious adverse events (Table 16), of which 1 subject in the 10 mg dose group experienced 2 serious adverse events that were unlikely related to the study drug, biliary obstruction and pain abdominal.

**Table 16 Summary of Adverse Events for TAB-004-01**

| | TAB004 0.3 mg/kg(N = 3) | | TAB004 1.0 mg/kg(N = 3) | | TAB004 3.0 mg/kg (N = 10) | | TAB004 10.0 mg/kg(N = 9) | | TAB004 in Total (N = 25) | |
|---|---|---|---|---|---|---|---|---|---|---|
| | Number of Events | Number of Subjects (%) | Number of Events | Number of Subjects (%) | Number of Events | Number of Subjects (%) | Number of Events | Number of Subjects (%) | Number of Events | Number of Subjects (%) |
| **All Adverse Events** | 25 | 3(100) | 28 | 3(100) | 49 | 10(100) | 66 | 9(100) | 168 | 25(100) |
| **Treatment-emergent Adverse Events (TEAEs)** | 25 | 3(100) | 25 | 3(100) | 42 | 10(100) | 54 | 9(100) | 146 | 25(100) |
| **Dose-limiting Toxicity Adverse Events (DLTs)** | 0 | 0 | 0 | 0 | 0 | 0 | 0 | 0 | 0 | 0 |
| **Serious Adverse Events (SAEs)** | 4 | 1(33.3) | 5 | 2(66.7) | 0 | 0 | 5 | 4(44.4) | 14 | 7(28) |
| **Related to the Study Drug** | 0 | 0 | 0 | 0 | 0 | 0 | 2 | 1(11.1) | 2 | 1(4) |
| **Immune-related Adverse Events (irAEs)** | 0 | 0 | 7 | 1(33.3) | 3 | 2(20) | 1 | 1(11.1) | 11 | 4(16) |
| **Related to the Study Drug** | 0 | 0 | 6 | 1(33.3) | 3 | 2(20) | 1 | 1(11.1) | 10 | 4(16) |
| **Adverse Events Leading to Dose Interruption/Discontinuation** | 1 | 1(33.3) | 0 | 0 | 0 | 0 | 2 | 2(22.2) | 3 | 3(12) |
| **Related to the Study Drug** | 0 | 0 | 0 | 0 | 0 | 0 | 1 | (11.1) | 1 | 1(4) |
| **Adverse Events with Outcome of Death** | 0 | 0 | 0 | 0 | 0 | 0 | 0 | 0 | 0 | 0 |
| **Related to the Study Drug** | 0 | 0 | 0 | 0 | 0 | 0 | 0 | 0 | 0 | 0 |

**Table 17 Summary of Serious Adverse Events for TAB-004-01**

| Subject No. | Name of Adverse Event | Onset Date | End Date | CTCAE Grade | Related to the Study Drug or Not | Outcome of Adverse Event | DLT or Not | Immune-related or Not |
|---|---|---|---|---|---|---|---|---|
| 2001-4002 | Small intestinal obstruction | 14-Apr-2020 | 17-Apr-2020 | 3 | Unrelated | Resolved | No | No |
| | Intestinal obstruction | 27-Jun-2020 | 01-Jul-2020 | 3 | Unrelated | Resolved | No | No |
| | Small intestinal obstruction | 05-Aug-2020 | 08-Aug-2020 | 3 | Unrelated | Resolved | No | No |
| | Intestinal obstruction | 28-Sep-2020 | Continuing | 3 | Unrelated | Not recovered/Not resolved | No | No |
| 2004-4002 | Abdominal pain | 01-Mar-2020 | 03-Mar-2020 | 3 | Unrelated | Resolved | No | No |
| | Constipation | 01-Mar-2020 | 03-Mar-2020 | 3 | Unrelated | Resolved | No | No |
| | Backpain | 15-Mar-2020 | 19-Mar-2020 | 3 | Unrelated | Resolved | No | No |
| 2004-4003 | Asthenia | 27-May-2020 | 29-May-2020 | 3 | Unrelated | Resolved | No | No |
| | Hyponatraemia | 27-May-2020 | 29-May-2020 | 3 | Unrelated | Resolved | No | No |
| 2004-4005 | Escherichia sepsis | 17-Jul-2020 | 22-Jul-2020 | 3 | Unrelated | Resolved | No | No |
| 2004-4010 | Peritonitis bacterial | 27-Sep-2020 | 06-Oct-2020 | 3 | Unrelated | Resolved | No | No |
| 2030-4003 | Haemoptysis | 18-Aug-2020 | 20-Aug-2020 | 3 | Unrelated | Resolved | No | No |
| 2035-4001 | Biliary obstruction | 17-Nov-2020 | Continuing | 3 | Unlikely related | Not recovered/Not | No | No |

| | | | | | | | | |
|---|---|---|---|---|---|---|---|---|
| | | | | | | resolved | | |
| | Abdominal pain | 17-Nov-2020 | 21-Nov-2020 | 2 | Unlikely related | Resolved | No | No |

**Table 18 Study Drug-related Adverse Events Occurring in ≥8% of Subjects in TAB-004-01**

**(by System Organ Class and Preferred Term)**

| | TAB004 0.3 mg/kg (N = 3) | | TAB004 1.0 mg/kg (N = 3) | | TAB004 3.0 mg/kg (N = 10) | | TAB004 10.0 mg/kg (N = 9) | | TAB004 in Total (N = 25) | |
|---|---|---|---|---|---|---|---|---|---|---|
| System Organ Class / Preferred Term | Number of Events | Number of Subjects (%) | Number of Events | Number of Subjects (%) | Number of Events | Number of Subjects (%) | Number of Events | Number of Subjects (%) | Number of Events | Number of Subjects (%) |
| Study drug-related adverse events | 12 | 2(66.7) | 7 | 1(33.3) | 14 | 6(60) | 20 | 5(55.5) | 53 | 14(56) |
| Gastrointestinal disorders | 2 | 2(66.7) | 0 | 0 | 2 | 2(20) | 7 | 4(44.4) | 11 | 9(36) |
| Abdominal pain | 0 | 0 | 0 | 0 | 1 | 1(10) | 1 | 1 (11.1) | 2 | 2(8) |
| Diarrhoea | 1 | 1(33.3) | 0 | 0 | 0 | 0 | 2 | 1 (11.1) | 3 | 3(12) |
| Nausa | 1 | 1(33.3) | 0 | 0 | 0 | 0 | 1 | 1 (11.1) | 2 | 2(8) |
| General disorders and administration site conditions | 2 | 1(33.3) | 2 | 1(33.3) | 1 | 1(10) | 4 | 3(33.3) | 9 | 6(24) |
| Pain | 1 | 1(33.3) | 0 | 0 | 0 | 0 | 1 | 1(11.1) | 2 | 2(8) |
| Asthenia | 1 | 1(33.3) | 1 | 1(33.3) | 1 | 1(10) | 1 | 1(11.1) | 4 | 4(16) |
| Neoplasms benign, malignant and unspecified (including cysts and polyps) | 2 | 1(33.3) | 0 | 0 | 1 | 1(10) | 0 | 0 | 3 | 2(8) |
| Cancer pain | 2 | 1(33.3) | 0 | 0 | 1 | 1(10) | 0 | 0 | 3 | 2(8) |
| Musculoskeletal and connective tissue disorders | 3 | 2(66.7) | 0 | 0 | 2 | 2(20) | 1 | 1(11.1) | 6 | 5(20) |
| Bone pain | 1 | 1(33.3) | 0 | 0 | 1 | 1(10) | 0 | 0 | 2 | 2(8) |
| Skin and subcutaneous tissue disorders | 1 | 1(33.3) | 1 | 1(33.3) | 3 | 3(30) | 1 | 1(11.1) | 6 | 6(24) |
| Pruritus | 1 | 1(33.3) | 0 | 0 | 1 | 1(10) | 1 | 1(11.1) | 3 | 3(12) |
| Urticarial | 0 | 0 | 1 | 1(33.3) | 1 | 1(10) | 0 | 0 | 2 | 2(8) |

2) **Efficacy results** Of the 16 efficacy evaluable subjects, 1 with melanoma (3 mg/kg dose group) achieved PR and 6 (2 with intestinal carcinoma, 1 with sarcoma, 1 with neuroendocrine tumor, 1 with non-small cell lung cancer, and 1 with head and neck squamous cell carcinoma) achieved SD.

3) **Pharmacokinetics** The average serum concentration versus time curve of TAB004 after intravenous infusion of 0.3, 1, 3, 10 mg/kg for the first dose and the fourth cycle is shown in Figure 3. Pharmacokinetic parameters are presented in Table 19.

**Table 19 Pharmacokinetic Parameters of TAB004 Following Intravenous Infusion**

| Dosage mg/kg | Cycle | n | $T_{1/2}$ hr | $T_{max}$ hr | $C_{max}$ μg/mL | $AUC_{0-last}$ hμg/mL | $AUC_{0-INF}$ hμg/mL | CL ml/h/kg | $V_{ss}$ ml/kg |
|---|---|---|---|---|---|---|---|---|---|
| 0.3 | C1 | 3 | 251.32±131.32 | 2.33[1.33, 2.37] | 7.01±2.02 | 1529.85±381.57 | 2052.70±638.61 | 0.21±0.05 | 76.26±44.75 |
| | C4 | 1 | 60.95 | 1.62[1.62, 1.62] | 6.77 | 735.46 | 737.13 | 0.41 | 34.6 |
| 1 | C1 | 3 | 255.22±70.60 | 2.00[1.03, 2.00] | 23.24±3.55 | 4491.37±529.25 | 5916.34±1182.53 | 0.23±0.03 | 78.08±22.65 |
| | C4 | 1 | 120.06 | 1.53[1.53, 1.53] | 25.68 | 4270.05 | 4493.15 | 0.23 | 33.27 |
| 3 | C1 | 9 | 432.73±174.88[a] | 1.17[1.03, 6.75] | 59.90±14.30 | 12752.43±3305.86[b] | 22791.59±5882.04[b] | 0.24±0.06[b] | 143.03±76.32[b] |
| | C4 | 5 | 501.77±179.10 | 0.68[0.52, 1.67] | 94.09±23.36 | 24110.38±7107.78 | 46301.66±14728.24 | 0.13±0.04 | 85.88±28.94 |
| 10 | C1 | 9 | 302.02±116.83 | 2.15[0.57, 25][b] | 210.49±35.89[b] | 38683.42±8498.07 | 59171.32±15751.77 | 0.26±0.06 | 103.33±43.18 |
| | C4 | 2 | 370.37[c] | 3.94[1.47, 6.42] | 308.42±30.65 | 54983.93[c] | 117016.02[c] | 0.14[c] | 73.27[c] |

n: number of subjects; a: n=8; b: n=8; c: n=1

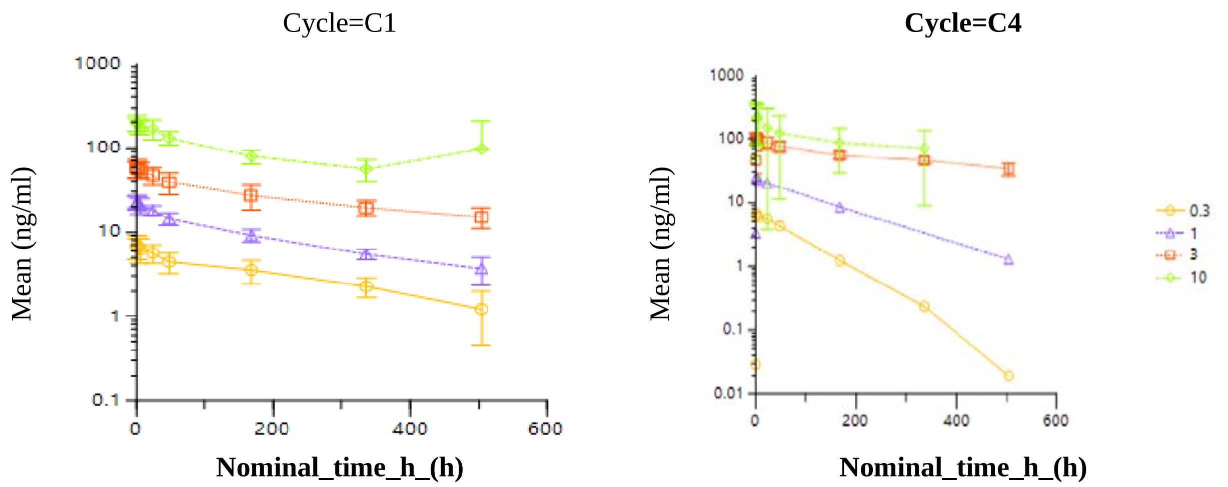


**Figure 3 Mean Serum Concentration Versus Time Curve after Intravenous Infusion of TAB004 (C1: Cycle 1; C4: Cycle 4)**

The preliminary pharmacokinetic data after the first dose showed that the serum exposure of TAB004 increased with the increase of dose within the dose range of 0.3-10 mg/kg, basically showing a linear pharmacokinetic profile. After intravenous drip of TAB004 at different doses (0.3, 1, 3, 10 mg/kg, Q3W), the mean maximum serum concentration ($C_{max}$) increased in direct proportion to the dose, and the $C_{max}$ was 7.01±2.02 μg/mL, 23.24±3.55 μg/mL, 59.90±14.30 μg/mL and 210.49±35.89 μg/mL, respectively; The mean $AUC_{(0-t)}$ was approximately proportional to the dose, and the $AUC_{(0-t)}$ was 1529.85±381.57 hr*μg/mL, 4491.37±529.25 hr*μg/mL, 12752.43±3305.86 hr*μg/mL and 38683.42±8498.07 hr*μg/mL, respectively; The mean elimination half-life ($t_{1/2}$) was 251.32±131.32 hr, 255.22±70.60 hr, 432.73±174.88 hr and 302.02±116.83 hr, respectively. The CL and Vd of JS004 were consistent with the human pharmacokinetic profile of a typical IgG4 antibody.

After multiple intravenous infusions of JS004 at different doses (0.3, 1, 3, 10 mg/kg, Q3W), the serum concentration reached the steady-state level after 4 consecutive doses, the mean trough concentration ($C_{trough}$) after multiple doses was 35.18±8.97 μg/mL in the 3 mg/kg (n=5) dose group, the half-life was 501.77±179.10 hr (20.91±7.46 days), and the $C_{max}$ and $AUC_{(0-t)}$ was 94.09±23.36 μg/mL and 24110.38±7107.78 hr*μg/mL, respectively.

#### 1.1.8.1.2. Study JS004-001-I

In Jan. 2020, the "Clinical Trial Notification" issued by the National Medical Products Administration was obtained for JS004. The first subject was administered in the phase I study of advanced solid tumors in China on 28 Apr. 2020. This was a phase I study of recombinant humanized anti-BTLA monoclonal antibody (JS004) in patients with advanced solid tumors. The primary endpoints included safety evaluation, maximum toxic dose (MTD) and recommended phase 2 dose (RP2D). The study consisted of 3 stages: dose escalation, dose expansion and indication expansion. Patients with advanced solid tumors who had failed standard treatment and had no effective treatment were enrolled. Dose escalation phase: The traditional 3+3 dose escalation design was adopted, and 4 dose groups were planned, including 0.3 mg/kg, 1mg/kg, 3 mg/kg and 10 mg/kg. JS004 would be administered intravenously once every 3 weeks. The DLT observation period for each subject was 3 weeks after the first dose. If none of the 3 subjects in a dose group experienced DLT within 3 weeks, the next dose escalation might be performed. If 1 of 3 subjects experienced DLT, 3 additional subjects should be included. The next dose escalation would be performed if no DLT was observed in the additional 3 subjects. If ≥1 DLT event recurred in the additional 3 subjects, dose escalation would be terminated, and the previous dose would be defined as the MTD. Subject expansion phase: A specific dose group would be selected. This phase could be started after the completion of escalation in the corresponding dose group, with about 9-12 subjects enrolled in each dose group. Indication expansion phase: Specific tumor types would be selected for indication expansion after the completion of subject expansion. A maximum of 40 subjects would be enrolled for each indication. The dose would be administered by intravenous injection at RP2D, Q3W.

As of 28 Feb. 2021, a total of 11 subjects were enrolled. Of the 11 subjects, 3 subjects received 0.3 mg/kg, 3 received 1 mg/kg, 4 received 3 mg/kg, and 1 received 10 mg/kg. Safety data were available for a total of 11 subjects who received at least 1 dose of JS004 (Table 20). No DLT occurred, but 3 subjects (27.3%) experienced adverse events leading to study drug interruption or discontinuation, with 2 subjects in the 0.3 mg/kg dose group and 1 in the 3 mg/kg dose group. One of these subjects experienced a study drug-related adverse event leading to dose interruption. All these 11 subjects experienced adverse events that were related to the study drug treatment at the discretion of the investigator (Table 20). The common drug-related adverse events were thyroid function decreased (45.5%), electrocardiogram QT prolongation (36.4%), proteinuria (36.4%), blood lactate dehydrogenase increased (36.4%), hypoalbuminemia (36.4%), -glutamyltransferase increased (27.3%), hypertension (27.3%), and sinus tachycardia (27.3%), etc. (Table 22). One subject (9.1%) experienced a serious adverse event (Table 21) that was unrelated to the study drug at the discretion of the investigator.

**Table 20 Summary of Adverse Events for JS004-001-I**

| | JS004 0.3 mg/kg(N= 3) | | JS004 1.0 mg/kg(N= 3) | | JS004 3.0 mg/kg(N= 4) | | JS004 10 mg/kg(N= 1) | | JS004 in Total (N=11) | |
|---|---|---|---|---|---|---|---|---|---|---|
| | Number of Events | Number of Subjects (%) | Number of Events | Number of Subjects (%) | Number of Events | Number of Subjects (%) | Number of Events | Number of Subjects (%) | Number of Events | Number of Subjects (%) |
| **All Adverse Events** | 140 | 3(100) | 48 | 3(100) | 100 | 4(100) | 11 | 1(100) | 299 | 11(100) |
| **Treatment-emergent Adverse Events (TEAE)** | 103 | 3(100) | 36 | 3(100) | 63 | 4(100) | 6 | 1(100) | 208 | 11(100) |
| **Related to the Study Drug** | 24 | 3(100) | 21 | 3(100) | 36 | 4(100) | 1 | 1(100) | 82 | 11(100) |
| **Dose-limiting Toxicity Adverse Events (DLT )** | 0 | 0 | 0 | 0 | 0 | 0 | 0 | 0 | 0 | 0 |
| **Serious Adverse Events** | 2 | 1(33.3) | 0 | 0 | 0 | 0 | 0 | 0 | 2 | 1(9.1) |
| **Related to the Study Drug** | 0 | 0 | 0 | 0 | 0 | 0 | 0 | 0 | 0 | 0 |
| **Immune-related Adverse Events (irAEs)** | 0 | 0 | 0 | 0 | 0 | 0 | 0 | 0 | 0 | 0 |
| **Related to the Study Drug** | 0 | 0 | 0 | 0 | 0 | 0 | 0 | 0 | 0 | 0 |
| **Adverse Events Leading to Dose Interruption/Discontinuation Adverse Events** | 6 | 2(66.7) | 0 | 0 | 1 | 1(25.0) | 0 | 0 | 7 | 3(27.3) |
| **Related to the Study Drug** | 0 | 0 | 0 | 0 | 1 | 1(25.0) | 0 | 0 | 1 | 1(9.1) |
| **Adverse Events with Outcome of Death** | 2 | 1(33.3) | 0 | 0 | 0 | 0 | 0 | 0 | 2 | 1(9.1) |
| **Related to the Study Drug** | 0 | 0 | 0 | 0 | 0 | 0 | 0 | 0 | 0 | 0 |

**Table 21 Summary of Serious Adverse Events for JS004-001-I**

| Subject No. | Name of Adverse Event | Onset Date | End Date | CTCAE Grade | Related to the Study Drug or Not | Outcome of Adverse Event | DLT or Not | Immune-related or Not |
|---|---|---|---|---|---|---|---|---|
| 01001 | Intestinal subobstruction | 24-May-2020 | 19-Jun-2020 | 3 | Definitely unrelated | NA | No | No |
| 01001 | Lung infection | 24-May-2020 | 19-Jun-2020 | 3 | Definitely unrelated | NA | No | No |

**Table 22 Study Drug-related Adverse Events in JS004-001-I**

| | N=11 | (%) |
|---|---|---|
| **Common (≥10%) drug-related TRAEs** | **All grades** | **Grade 3 and above** |
| **TRAE occurring at least once** | 11(100) | 1(9.1) |
| Hypothyroidism | 5(45.5) | 0 |

| | | |
|---|---|---|
| Proteinuria | 4 (36.4) | 0 |
| Hypoproteinaemia | 4 (36.4) | 0 |
| Electrocardiogram QT prolongation | 4 (36.4) | 1 (9.1) |
| Blood lactate dehydrogenase increased | 4 (36.4) | 0 |
| Hypertension | 3 (27.3) | 0 |
| Hyperthyroidism | 3 (27.3) | 0 |
| Blood alkaline phosphatase increased | 3 (27.3) | 0 |
| Sinus tachycardia | 3 (27.3) | 0 |
| Gamma-glutamyltransferase increased | 2 (18.2) | 0 |
| Abdominal distension | 2 (18.2) | 0 |
| Hyperglycaemia | 2 (18.2) | 0 |
| Lymphocyte count decreased | 2 (18.2) | 0 |
| Protein urine detected | 2 (18.2) | 0 |

1) **Efficacy results:** Of the 7 efficacy evaluable subjects, 3 subjects achieved SD (1 with ovarian cancer, 1 with breast cancer, and 1 with lung squamous cell carcinoma).

2) **Pharmacokinetics:** The pharmacokinetic analysis data of JS004 after multiple cycles of intravenous drip of 0.3, 1, and 3 mg/kg have not been obtained, and the mean serum concentration versus time curve after the first dose is shown in Figure 4, and PK parameters are presented in Table 23.

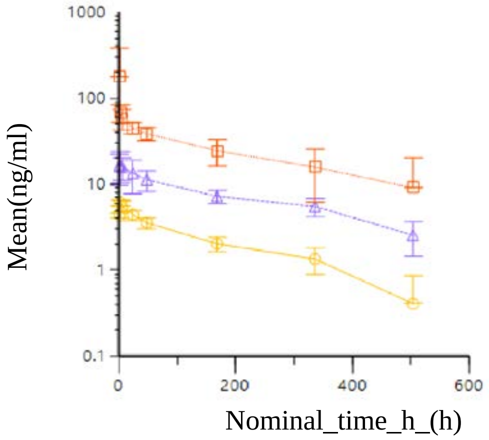


**Figure 4 Mean Serum Concentration Versus Time Curve of JS004 after Intravenous Infusion in Cycle 1**

The preliminary pharmacokinetic data after the first dose showed that the serum exposure of JS004 increased with the increase of dose within the dose range of 0.3-3 mg/kg, basically showing a linear pharmacokinetic profile. After intravenous drip of JS004 at different doses (0.3, 1, 3 mg/kg, Q3W), the mean maximum serum concentration ($C_{max}$) increased in direct proportion to the dose, and the $C_{max}$ was 6.13±0.52 µg/mL, 17.00±6.50 µg/mL and 64.50±0.14 µg/mL, respectively; The mean $AUC_{(0-t)}$ was approximately proportional to the dose, and the $AUC_{(0-t)}$ was 932.11±131.05 hr*µg/mL, 3508.55±329.45 hr*µg/mL and 11988.79±4445.36 hr*µg/mL, respectively; The mean elimination half-life ($t_{1/2}$) was 160.37±59.40 hr, 254.08±104.58 hr and 195.30±117.81 hr, respectively. The CL and Vd of JS004 were consistent with the human pharmacokinetic profile of a typical IgG4 antibody.

**Table 23 Pharmacokinetic Parameters of JS004 after Intravenous Drip in Cycle 1 (n=3)**

| Dose (mg/kg) | $T_{1/2}$ hr | $T_{max}$ hr | $C_{max}$ µg/mL | $AUC_{0\text{-}last}$ h *µg/mL | $AUC_{0\text{-}INF}$ h*µg/mL | CLml/h/kg | $V_{ss}$ml/kg |
|---|---|---|---|---|---|---|---|
| 0.3 | 160.37±59.40 | 2.0[2.00,2.00] | 6.13±0.52 | 932.11±131.05 | 1130.32±285.63 | 0.31±0.06 | 67.85±13.41 |
| 1 | 254.08±104.58 | 2.00[2.00,6.00] | 17.00±6.50 | 3508.55±329.45 | 4555.88±329.45 | 0.29±0.05 | 106.75±54.18 |
| 3 | 195.30±117.81 | 1.00[1.00,1.00] | 64.50±0.14 # | 11988.79±4445.36 | 14462.97±8440.44 | 0.29±0.09 | 66.70±13.91 |

## 1.2. Scientific Rationale

In accordance with the Drug Registration Regulation, Good Clinical Practice and Guidelines for Clinical Pharmacokinetic Studies of New Drugs (Chemicals), the study intends to evaluate the tolerability, safety, pharmacokinetics and preliminary efficacy of JS004 monotherapy and in combination with toripalimab in patients with relapsed/refractory malignant lymphoma, to provide a basis for subsequent clinical studies.

## 1.3. Potential Risks and Benefits

Patients may generally experience fever, headache, nausea, vomiting or hypotension after receiving monoclonal antibodies. Hypersensitivity or allergic (e.g. shortness of breath, urticaria, angioedema) reactions may occur after injection of any protein drug. Immune complex-mediated type 3 hypersensitivity reactions are similar in AEs to type 1 reactions, but may be delayed after infusion, and symptoms may include rash, urticaria, polyarthritis, myalgia, polysynovitis, fever, and, if severe, glomerulonephritis. Patients with clinical signs of allergic reaction/hypersensitivity should be closely observed during the investigational product infusion and continue to be monitored thereafter for clinical signs of systemic reactions.

Appropriate medical therapy and other supportive measures should be provided according to local guidelines to manage hypersensitivity or allergic reactions. Monitoring and treatment guidelines are provided in the study protocol.

# 2. Study Objectives and Endpoints

## 2.1. Study Objectives

**Primary Objectives:**

- To evaluate the safety and tolerability of JS004monotherapy and in combination with toripalimab in patients with relapsed/refractory malignant lymphoma;
- To observe the dose-limiting toxicity (DLT) of JS004 monotherapy and in combination with toripalimab in patients with relapsed/refractory malignant lymphoma and to determine the maximum tolerated dose (MTD) and recommended phase II dose (RP2D). When no MTD is observed, the maximum dose of JS004 specified in the protocol is taken as the maximum feasible dose (MFD).

**Secondary Objectives:**

- To evaluate the pharmacokinetic (PK) profile of JS004 monotherapy and in combination with toripalimab;
- To evaluate BTLA receptor occupancy (RO) on peripheral blood immune cells;
- To evaluate the effect of JS004 monotherapy and in combination with toripalimab on peripheral blood cytokines;
- To preliminarily evaluate the anti-tumor activity of JS004 monotherapy and in combination with toripalimab, including objective response rate (ORR), duration of response (DoR), disease control rate (DCR), best overall response (BoR), progression-free survival (PFS) and overall survival (OS);
- To evaluate the immunogenicity of JS004 monotherapy and in combination with toripalimab.

**Exploratory Objectives:**

- To evaluate the effect of JS004 monotherapy and in combination with toripalimab on peripheral blood immune cell subtypes;
- To investigate biomarkers possibly associated with JS004 activity, including BTLA, HVEM and PD-L1, their expression in tumor tissues, the CD8 positive tumor-infiltrating immune cell density, whole exome sequencing (WES) of tumor tissue and paired blood, peripheral blood immune cell surface receptors (PD-1, HVEM, CTLA-4, CD112R, TIM-3, ICOS, CX3CR1, CD183, and CD103), and their relationship with treatment response.

## 2.2. Study Endpoints

**Primary Endpoints**

- Safety evaluation: Incidence and severity of adverse events/serious adverse events (graded according to CTCAE 5.0); and incidence of Grade ≥3 adverse events;
- MTD and RP2D.

**Secondary Endpoints**

- PK parameters: including but not limited to maximum serum concentration ($C_{max}$), minimum serum concentration ($C_{min}$), time to reach maximum serum concentration ($T_{max}$), area under the serum concentration versus time curve from time zero to infinity ($AUC_{0-\infty}$), area under the serum concentration versus time curve from time zero to the last measurable concentration timepoint ($AUC_{0-t}$), area under the serum concentration versus time curve from time zero to 21d ($AUC_{0-21d}$), terminal half-life ($t_{1/2}$), clearance (CL), volume of distribution ($V_{ss}$) and accumulation ratio (Rac), etc.;
- Pharmacodynamic assessment: BTLA receptor occupancy (RO) on CD3+, CD4+CD45RA+, CD8+CD45RA+, CD3-CD20+, and CD3-CD56+ cells;
- Effect of JS004 on peripheral blood cytokines (IFN-γ, IL-8, IL-4, IL-6, IL-10, and TNF-α) ;
- Efficacy evaluation: Objective response rate (ORR), duration of response (DoR), disease control rate (DCR), progression-free survival (PFS) and overall survival (OS) evaluated per Lugano 2014 Criteria;
- Immunogenicity assessment: Anti-drug antibodies (ADAs) and/or neutralizing antibodies (Nabs).

**Exploratory Endpoints**

- Effect of JS004 on peripheral blood immune cell subtypes (CD4+, CD3+CD4+/CD3+CD8+, CD20+, CD3-CD20-NKG2A+ cells);
- Biomarkers possibly associated with JS004 activity, including BTLA, HVEM and PD-L1, their expression in tumor tissues, the CD8 positive tumor-infiltrating immune cell density, whole exome sequencing (WES) of tumor tissue and paired blood, peripheral blood immune cell surface receptors (PD-1, HVEM, CTLA-4, CD112R, TIM-3, ICOS, CX3CR1, CD183, and CD103), and their relationship with treatment response.

## 3. Study Design

### 3.1. Overall Design

This is a multicenter, open-label, dose escalation and dose expansion phase I study of JS004 monotherapy and in combination with toripalimab in Chinese patients with relapsed/refractory malignant lymphoma. The study consists of Part A and Part B. Each phase of the study includes a screening period (up to 28 days before the subject signs the informed consent form and before the first dose of study drug), a treatment period (from the first dose of study drug to drug discontinuation), and a follow-up period (safety and survival follow-up).

The study process includes a screening period of up to 28 days (from the subject's signature of the informed consent until before the first dose of the study drug), a treatment period (from the first dose of the study drug to drug discontinuation), and a follow-up period (safety follow-up and survival follow-up).

**<u>Part A: JS004 Monotherapy</u>**

1) Dose escalation phase: The "3+3" dose escalation design is adopted, and 3 dose groups are planned, including 1 mg/kg, 3 mg/kg and 10 mg/kg. All doses are administered as intravenous drip. The 10 mg/kg dose is the pre-specified maximum administered dose (MAD) with an interval of 3 weeks between each administration. However, the dose level or administration interval may be adjusted during the study after new data becomes available.

Eligible subjects are enrolled sequentially and receive escalating doses from low to high. Each subject can only be assigned to one dosing regimen or interval schedule. There is an interval of at least 7 days between the first and second subjects in each dose group. The DLT observation period for each subject is 3 weeks after the first dose. If none of the 3 subjects in a dose group experiences DLT within 3 weeks, the next dose escalation may be performed. If 1 of 3 subjects experiences DLT, 3 additional subjects should be included. The next dose escalation will be performed if no DLT is observed in the 3 additional subjects. If ≥1 DLT event recurs in the additional 3 subjects, dose escalation will be terminated, and the previous dose will be defined as the MTD. After completing DLT observation in the highest dose group specified in the protocol, dose escalation will not be continued even if the above discontinuation criteria are not met.

2) Dose expansion phase: The dose levels are 3 mg/kg and 200 mg in this phase to further confirm the safety and pharmacokinetic profile of monotherapy and explore the mode of administration at a fixed dose, aiming to simplify the clinical administration of JS004. Approximately 6-9 subjects will be enrolled in each dose group in this phase, and the number of subjects can be adjusted subsequently according to study progress. In this phase, the requirements for subject inclusion, mode of administration and study procedures are the same as those in the first phase. Evaluations will be performed for the safety, tolerability, PK, PD, immunogenicity and preliminary efficacy in the same manner. Any DLT or delayed DLT that may occur in this phase will not be used as the basis for dose escalation, but as a reference for subsequent clinical studies in terms of dose design.

Based on all safety, PK, and PD data during dose escalation, the RP2D may be the MTD/MAD or the dose below the MTD/MAD. The RP2D will be determined based on the study progress. Any delayed immune-related DLT occurring after the first period of treatment may also be regarded as a reference for the RP2D assessment. The study in this phase can be conducted concurrently with that in Part B dose escalation phase.

3) Indication expansion phase: Peripheral T-cell lymphoma, Hodgkin lymphoma, follicular lymphoma or other subtypes are preferred for indication expansion, which can be adjusted according to study progress. Approximately 15 subjects are enrolled for each indication expansion (including the number of subjects in the dose expansion phase; the number of cases can be adjusted according to study progress). All doses are administered by intravenous injection at RP2DA, Q3W. Treatment will continue in a 21-day cycle until progressive disease, intolerable toxicity, treatment with JS004 (since the first dose) for more than 2 years or other reasons as specified in the protocol. During this phase, all study procedures will remain the same as in the dose expansion phase, with the exception that intensive PK (pharmacokinetic) sampling will not be carried out and blood samples for PD (pharmacodynamic), cytokine, and immune cell subtype studies will not be collected.

**Part B: JS004 in Combination with Toripalimab**

1) Dose escalation phase: The "3+3" dose escalation design is adopted, and 2 dose groups are preliminarily planned. Toripalimab is administered at 240 mg, and JS004 will be administered at fixed doses of 100 mg and 200 mg at a dosing interval of Q3W. The DLT observation period is 21 days after the first dose.

Each dose group will first include 3 subjects. If the first subject experiences no DLT within the DLT evaluation window (21 days) at a given dose, the study will proceed to the next dose level (dose escalation is not allowed in the same subject). If 1 of 3 subjects experiences DLT, 3 additional subjects will be enrolled in the current dose group; If DLT occurs in ≥2 subjects in a dose group of 3 or 6 subjects, dose escalation will be terminated, and the investigator and the sponsor will decide whether to terminate dose escalation or consider dose modification for exploration upon discussion. The maximum tolerated dose (MTD) is defined as the maximum dose at which DLT occurs in <1/3 of the patients. The dose level, administration interval and number of enrolled subjects may be adjusted by the Safety Monitoring Committee (SMC) upon discussion during the study according to the progress of other studies of JS004 and the safety, PK and PD data available.

2) Dose expansion phase: The recommended dose group of the combination is selected for expansion, and 6-9 subjects will be enrolled in each group. Subsequently, the number of subjects can be adjusted according to study progress to better confirm the safety of the combination and determine the recommended dose for expansion (RDE) for the indication expansion phase.

The RP2DB will be discussed and determined by the Safety Monitoring Committee (SMC) based on all safety, PK, PD and other data obtained during dose escalation and in this phase, as well as the progress of other studies of JS004. If the subject is tolerable at the discretion of the investigator after completing the DLT evaluation, the subject will continue to be administered at the same dose until progressive disease, intolerable toxicity, treatment with JS004 and/or toripalimab (since the first dose) for more than 2 years or other reasons specified in the protocol.

3) Indication expansion phase: Peripheral T-cell lymphoma, Hodgkin lymphoma, follicular lymphoma or other subtypes are preferred for indication expansion, which can be adjusted according to study progress. Approximately 20 subjects are enrolled for each indication expansion (including the number of subjects in the dose expansion phase). The number of cases can be adjusted according to study progress. All doses are administered by intravenous injection at RP2DB, Q3W, in combination with toripalimab 240 mg, Q3W. Treatment will continue in a 21-day cycle until progressive disease, intolerable toxicity, treatment with JS004 and/or toripalimab (since the first dose) for more than 2 years or other reasons as specified in the protocol. During this phase, all study procedures will remain the same as in the dose expansion phase, with the exception that intensive PK (pharmacokinetic) sampling will not be carried out and blood samples for PD (pharmacodynamic), cytokine, and immune cell subtype studies will not be collected.

In Part A, subjects who have progressive disease but are still benefiting from anti-BTLA treatment, as confirmed by the investigator, or who have stable disease for at least two tumor assessments, may proceed to Part B to receive the recommended dose of the combination with toripalimab (subjects with progressive disease should provide informed consent for continuing treatment after progression). Once enrolled in Part B, the study procedures will follow the protocols outlined for this phase.

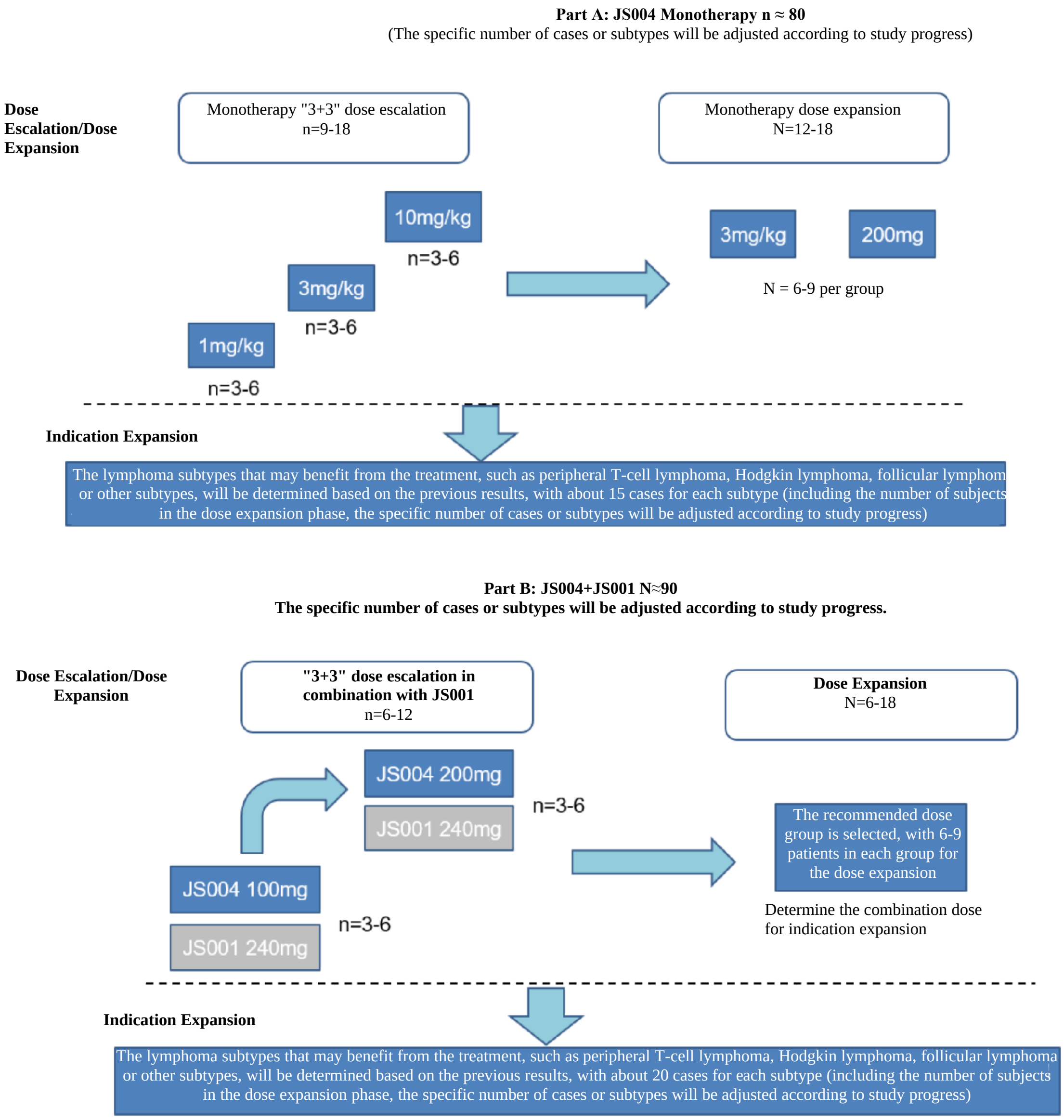


**Figure 5 Overall Study Design Diagram**

### 3.1.1. Determination of Starting Dose for Escalation

**Part A: Selection of Starting Dose of JS004**

1) When JS004 was administered to mice at 1, 3, and 10 mg/kg (BIW×7) to determine its in vivo anti-tumor activity, no clear dose response was found, but 1 mg/kg JS004 resulted in a TGI rate <50%, so we may predict that the proposed starting dose of 0.3 mg/kg once every 3 weeks will not reach 50% of the human pharmacological activity, This activity represents the MABEL in vivo and is more biologically relevant.
2) In the GLP-compliant repeat-dose toxicity study in cynomolgus monkeys (the corresponding mean $C_{max}$ and AUC values after the fifth dose were 4.266 mg/mL and 341.061 mg•hr/mL, respectively), the well-tolerated dose was determined to be 100 mg/kg; The equivalent human dose is 100 mg/kg; The maximum recommended starting dose (MRSD) is determined to be 16.66 mg/kg (1/6 of the well-tolerated dose of 100 mg/kg) in humans.
3) The starting dose for the first-in-human (FIH) study of JS004 is estimated to be 0.3 mg/kg after considering the minimal anticipated biological effect level (MABEL), receptor occupancy in cynomolgus monkeys (RO), pharmacologically active dose (PAD), and no-observed-adverse-effect level. The preliminary clinical data of the first-in-human study of JS004 conducted in the US showed that JS004 was well tolerated in the 0.3 mg/kg and 1 mg/kg dose groups, and no DLT was observed.

The starting dose is calculated in combination with the above different methods and the recommended dose is 0.3 mg/kg to 16.66 mg/kg. Considering that this product is the mechanism of action, the clinical starting dose is calculated by using the minimum anticipated biological effect level (MABEL) method recommended in the guidelines. Considering comprehensively, 1mg/kg is taken as the starting dose for this product.

The levels and dosing intervals for dose escalation will be adjusted as necessary during the study based on the available safety, tolerability, PK and PD data.

**Part B: Selection of Starting Dose of JS004**

1) JS004 was well tolerated in the single-agent phase I study (TAB004-001), and no DLT and unexpected adverse reactions were observed. JS004 showed linear pharmacokinetic profile at 0.3-10 mg/kg. In different dose groups (0.3, 1, 3, 10 mg/kg, Q3W), full BTLA receptor occupancy could be maintained during treatment. The mean steady-state minimum serum concentration of the 3 mg/kg dose group was approximately 35.18 μg/mL, which is more than 10-fold higher than the saturating concentration of BTLA receptors (approximately 3 μg/mL) in in-vitro experiments. Of the 16 subjects evaluable for efficacy, 1 subject achieved PR (3 mg/kg dose group) and 6 achieved SD. No increase in the incidence or severity of adverse events was observed with increasing dose levels. The RP2D of JS004 was determined to be 3 mg/kg, Q3W (3 mg/kg Q3W is approximately equivalent to 200 mg, Q3W, based on the average subject weight of 60 kg), based on the results of pharmacokinetics, receptor occupancy and initial efficacy.

2) The pharmacokinetic and receptor occupancy results obtained in this study were similar to those obtained in the US phase 1 study.

Most monoclonal antibodies show a large safety margin and a relatively flat dose-response relationship. Clinical studies of multiple monoclonal antibody drugs have shown that fixed-dose administration and weight-based administration have similar drug exposures, suggesting that weight may not be a key factor, which affects monoclonal antibody drug exposure. According to the preliminary clinical data from the studies conducted, JS004 has a large safety margin, which is similar to most monoclonal antibody drugs, and no clear relationship has been found between the patient weight and drug PK, efficacy/safety within the investigated dose range. Therefore, evaluation of fixed dose is acceptable and is expected to simplify the clinical administration of JS004. Considering that the strength is 100 mg/vial, a fixed dose of 100 mg (≤1/2 of the RP2D dose of JS004 monotherapy) is selected as the starting dose of JS004 in combination with toripalimab.

**Dose Selection of Toripalimab:**

The recommended dose in the package insert is 3 mg/kg, once every 2 weeks. In vitro experiments showed that toripalimab at a concentration >20 nM or 3 μg/mL enabled complete saturation of PD-1 receptors on the surface of T cells. Given the inaccessibility of antibody macromolecules to the tumor microenvironment, a concentration of toripalimab maintained at 20 μg/mL in peripheral blood is sufficient to ensure PD-1 receptors on lymphocytes being completely occupied. The peak and trough concentrations under the steady state of 240 mg Q3W regimen were 85 μg/mL and 20 μg/mL, respectively, which were similar to the peak and trough concentrations under the steady state of 93 μg/mL and 34 μg/mL for 3 mg/kg Q2W regimen in terms of drug exposure and steady-state concentration. It could maintain PD-1 receptors being completely occupied. The pharmacokinetics, receptor occupancy and phase II pivotal studies of toripalimab in melanoma supported a fixed dose of 240 vmg (Q3W) as the recommended dose of this study.

**Dose Selection of Combination Therapy:**

A fixed dose of 240 mg of toripalimab will be used in the combination dose escalation, and JS004 will be administered at fixed doses of 100 mg and 200 mg at a dosing interval of Q3W. The levels and dosing intervals for dose escalation may be adjusted as necessary based on the available safety, pharmacokinetic (PK) and pharmacodynamic (PD) results.

### 3.1.2. Determination of Maximum Dose for Escalation

Eligible subjects are enrolled sequentially and receive escalating doses from low to high. Each subject can only be assigned to one dosing regimen or interval schedule. Following a 28-day screening period, eligible subjects will be sequentially enrolled and receive their assigned JS004 dosing regimen. The pre-specified maximum administered dose (MAD) is 10 mg/kg Q3W, but the dose level or dosing interval (e.g., every 4 weeks, Q4W) can be adjusted during the study after new data becomes available.

### 3.1.3. Dose Escalation Design

The starting dose is 1 mg/kg in Part A, and the dose will be escalated in the order of 1, 3 and 10 mg/kg, with 3-6 subjects in each group. The starting dose is 100 mg in Part B and will be escalated to 200 mg, with 3-6 subjects in each group.

**Table 24 JS004 Dose Escalation Design**

| | Part A | | | Part B | | |
|---|---|---|---|---|---|---|
| Group No. | 1 | 2 | 3 | 1 | 2 | |
| Dose administered | 1 mg/kg | 3 mg/kg | 10 mg/kg | 100 mg | 200 mg | Decided by the SMC |
| Escalation ratio | / | 200% | 233% | / | 100% | |
| Number of subjects | 3~6 | 3~6 | 3~6 | 3-6 | 3-6 | …… |

### 3.1.4. Dose Escalation Principle

- If none of the subjects (0/3) in a dose group experiences a dose-limiting toxicity (DLT) in Cycle 1, 3 additional subjects will be enrolled to the next higher dose level.
- If 1 of 3 subjects in a dose group experiences a DLT in Cycle 1, 3 additional subjects will be enrolled to that dose group to expand the number of subjects to 6.
- If no DLT occurs in the 3 additional subjects (1 of 6 subjects in total experiences a DLT), escalation will proceed to the next higher dose level.

If 1 or more of the 3 additional subjects experience a DLT (≥2 of 6 subjects in total experience a DLT at this dose level), the dose escalation will be terminated, and the previous dose of this dose will be defined as the MTD. Alternatively, the Safety Monitoring Committee (SMC) may select an intermediate dose to further investigate the safety.

## 3.2. Definition of DLT

AEs will be graded according to the NCI CTCAE 5.0. The investigator will determine whether an AE is an immune-related event (irAE). Subjects receiving any dose of the study drug during the DLT observation period will be included in the DLT assessment. Toxicities that only occur in the DLT observation period can be used for assessing DLT and deciding whether to continue dose escalation. All AEs, except those confirmed to be caused by progressive disease or external factors, should be evaluated to determine whether they meet the criteria for DLT.

A DLT is defined as any of the following AE that occurs during Cycle 1 (21 days) and is possibly related to the study drug:

- Hematological toxicities:
  a. Grade ≥4 hematologic toxicity;
  b. Grade 3 thrombocytopenia with hemorrhage requiring platelet transfusion;
  c. Febrile neutrophils reduced.
- Non-hematological toxicities:
  a. Grade ≥3 non-hematologic toxicity, except:
    i. Grade 3 rash lasting <3 days;
    ii. Grade 3 nausea, vomiting or diarrhoea lasting <3 days after optimal medical management;
    iii. Grade 3 fatigue lasting <7 days or transient (lasting ≤6 h);
    iv. Medically manageable Grade 3 flu-like symptoms or fever that must last less than 3 days (72 h).
  b. Any Grade 3 or 4 non-hematologic laboratory abnormality provided that:
    i. Requiring medical intervention for the patient, or
    ii. Leading to hospitalization or lasting >72 h after appropriate alternative therapy. Except for Grade 3 laboratory abnormality that does not require hospitalization and is not clinically significant at the discretion of the investigator.
  c. Any ≥Grade 3 AST, ALT, or total bilirubin increased.
  d. Any AST or ALT >3×ULN accompanied by total bilirubin >2×ULN without cholestasis (alkaline phosphatase serum increased), i.e., meeting the FDA definition of potential drug-induced liver injury. (Note that this type of DLT should be based on ULN rather than CTC grade).
- All above-mentioned AEs of a specific grade that cannot be clearly attributed to progressive disease or other extraneous factors are considered DLTs.
- Death that cannot be clearly attributed to progressive disease or other extraneous factors.
- Other toxicities leading to premature termination of the study upon discussion between the investigators and the sponsor.
- TEAEs requiring dose interruption (Protocol Section 5.3.1.1 and Annex 3) that result in a ≥7-day delay in the start of Cycle 2 should be considered DLTs.
- TEAEs requiring permanent discontinuation (Protocol Section 5.3.1.2 and Annex 3) should be considered DLTs.

An AE that meets the above criteria but occurs beyond the DLT observation period is a delayed DLT. Delayed DLTs will be assessed by the investigators and the sponsor on a case-by-case basis, to decide whether the enrollment of subjects needs to be suspended upon discussion and propose the study design for the next step. Potential delayed DLTs should be assessed at least at each safety review (approximately every 6-8 weeks) before the decision on dose escalation. Once a delayed DLT is identified by the investigators and the sponsor, the same reporting rules as DLT will be followed.

### 3.3. Maximum Tolerated Dose (MTD) and Recommended Phase II Dose (RP2D)

During the dose escalation study, if 1 of 3 subjects experiences DLT, this dose group will be expanded to include 6 subjects ("3+3" method). If only 1 of 6 subjects experiences DLT, the dose escalation will continue. If 2 of 6 subjects experience DLT, the dose escalation should be terminated. The MTD is the maximum dose at which DLT occurs in ≤1/6 subjects in Cycle 1. The RP2D will be determined based on safety, PK/PD, immunogenicity, and preliminary anti-tumor activity results. The MTD and RP2D may be defined as the same dose level or different dose levels, but the RP2D cannot exceed the MTD. Delayed immune-related DLTs after Cycle 1 may also be considered for RP2D assessment.

### 3.4. Dose Expansion Phase

**Part A: JS004 Monotherapy**

The dose level in this phase will be determined to be 3 mg/kg according to the PK, PD and safety data of different dose groups in the first phase and JS004 Monotherapy Phase I Study in the US (TAB004-001). Additionally, the body weight of the monoclonal antibody may not be a key factor affecting its exposure, JS004 has large safety margins, and no clear relationship has been found between the body weight of the patient and the PK and efficacy/safety of the drug within the investigated dose range, as well as the mean body weight of the subject is 60 kg. So, a fixed dose group of 200 mg (approximately equivalent to 3 mg/kg) will be added in this phase to further confirm the safety and pharmacokinetic characteristics of monotherapy and explore the mode of administration of a fixed dose to simplify the clinical administration of JS004. This phase can be started after the completion of escalation in the corresponding dose group, with at least 9 subjects enrolled in each dose group. The number of subjects can be adjusted subsequently according to study progress. In this phase, the requirements for subject inclusion, mode of administration and study procedures are the same as those in the first phase, and evaluations will also be performed for the safety, tolerability, PK, PD, immunogenicity and preliminary efficacy. Any DLT or delayed DLT that may occur in this phase will not be used as the basis for dose escalation, but as a reference for subsequent clinical studies in terms of dose design.

**Part B: JS004 in Combination with Toripalimab**

The recommended dose group of the combination is selected for expansion, and 6-18 subjects are enrolled (if there are two dose groups for the recommended dose, 6-9 subjects will be enrolled in each group). Subsequently, the number of subjects will be adjusted according to study progress to better confirm the safety of the combination and determine the recommended dose for expansion (RDE) for the indication expansion phase.

The RP2DB will be discussed and determined by the Safety Monitoring Committee (SMC) based on all safety, PK, PD and other data obtained during dose escalation and in this phase, as well as the progress of other studies of JS004. If the subject is tolerable at the discretion of the investigator after completing the DLT evaluation, the subject will continue to be administered at the same dose until progressive disease, intolerable toxicity, treatment with JS004 and/or toripalimab (since the first dose) for more than 2 years or other reasons specified in the protocol.

## 3.5. Indication Expansion Phase

**Part A: JS004 Monotherapy**

To further evaluate the safety, tolerability and preliminary efficacy of JS004, peripheral T-cell lymphoma, Hodgkin lymphoma, follicular lymphoma or other subtypes are preferred for indication expansion, which can be adjusted according to study progress. Approximately 20 subjects are enrolled for each indication expansion (including the number of subjects in the dose expansion phase; the number of cases can be adjusted according to study progress). All doses are administered by intravenous injection at RP2DA, Q3W. During this phase, all study procedures will remain the same as in the dose expansion phase, with the exception that intensive PK (pharmacokinetic) sampling will not be carried out and blood samples for PD (pharmacodynamic), cytokine, and immune cell subtype studies will not be collected.

**Part B: JS004 in Combination with Toripalimab**

Peripheral T-cell lymphoma, Hodgkin lymphoma, follicular lymphoma or other subtypes are preferred for indication expansion, which can be adjusted according to study progress. Approximately 20 subjects are enrolled for each indication expansion (including the number of subjects in the dose expansion phase). The number of cases can be adjusted according to study progress. All doses are administered by intravenous injection at RP2DB, Q3W, in combination with toripalimab 240 mg, Q3W. Treatment will continue in a 21-day cycle until progressive disease, intolerable toxicity, treatment with JS004 and/or toripalimab (since the first dose) for more than 2 years or other reasons as specified in the protocol. During this phase, all study procedures will remain the same as in the dose expansion phase, with the exception that intensive PK (pharmacokinetic) sampling will not be carried out and blood samples for PD (pharmacodynamic), cytokine, and immune cell subtype studies will not be collected.

## 3.6. Safety Monitoring Committee

A Safety Monitoring Committee consisting of investigators and sponsor medical experts (with external experts as optional) will be established for this study to perform regular safety reviews for the study objectives of the protocol, including individual cases in the safety and clinical database and summary data. These reviews include, but are not limited to:

Decision on the escalation process, for example, deciding whether to continue dose escalation or the next escalation dose, and considering addition of an intermediate dose.

SAEs monitoring in accordance with regulatory guidance;

Discussions will be made between the investigator and the sponsor regarding AEs and laboratory changes at each dose level through regular teleconferences and/or meeting visits, aiming to determine the safety profile and risk/benefit rate and confirm whether to continue the enrollment.

## 3.7. Safety Meeting

Before starting the next dose level escalation, the investigator and the sponsor should discuss the available subject safety data via teleconference, web conference, or mail as soon as possible to decide whether to proceed to the next escalation level.

## 3.8. Pharmacokinetic and Pharmacodynamic Studies

**Pharmacokinetic (PK) blood sampling schedule:**

**JS004 PK sample collection:**

Non-compartmental analysis will be performed using WinNonlin 6.4 or above. PK parameters to be reported include but are not limited to $AUC_{0-\infty}$, $AUC_{0-t}$, $AUC_{0-21d}$, $C_{max}$, $C_{min}$, $T_{max}$, $t_{1/2}$, CL, $V_{ss}$ and Rac, etc. Dose proportionality will also be assessed.

Dose escalation and dose expansion phases of Part A and Part B:

Pharmacokinetic (PK) blood sampling will be performed: within 1 h before the first dose, at the end of the first dose (+5 min), 1 h (±5 min) after the end of the first dose, 6 h (±15 min), 24 h (1 h), 48 h (±2 h), 168 h (Day 8, ±8 h), 336 h (Day 15, ±12 h) and 504 h (Day 22, ±24 h, before administration in Cycle 2) after the start of the first dose for intensive blood sampling; within 1 h before administration in Cycle 2 (sample collection may not be repeated if there is no delay in Cycle 2) and at the end of dose (+5 min); In Cycle 4, intensive blood sampling will be performed within 1 h before administration, after the end of administration (+5 min), 1 h (±5 min) after administration, 6 h (±15 min), 24 h ±(1 h), 48 h ±(2 h), 168 h (Day 8, ±8 h), 336 h (Day 15, ±12 h), and 504 h (Day 22, ±24 h, before administration in Cycle 5) after the start of administration, within 1 h before the dose and at the end of administration in Cycles 6, 8, 12, 16, within 1 h before administration, at the end of treatment visit (±7 days), and at the safety follow-up visit every 8 cycles thereafter. The blood collection volume will be 3 mL.

Indication expansion phase of Part A and Part B:

Two millilters of blood samples will be collected to detect the blood concentration of JS004. Blood sampling timepoints: within 1 h before the dose of JS004 in Cycles 1, 2, 4, 6, 8, 12, 16, and every 8 cycles thereafter, at the end of treatment visit, and at the safety follow-up visit (if applicable).

**Toripalimab PK sample collection:**

Part B: Two millilters of blood samples will be collected to detect the blood concentration of toripalimab. Blood sampling timepoints: within 1 h before the dose of JS004 in Cycles 1, 2, 4, 6, 8, 12, 16 and every 8 cycles thereafter, at the end of treatment visit (±7 days), and at the safety follow-up visit (if applicable).

**Pharmacodynamic study:**

Dose escalation and dose expansion phases of Part A and Part B

The BTLA receptor occupancy (RO) on CD3+, CD4+CD45RA+, CD8+CD45RA+, CD3-CD20+, and CD3-CD56+ cells will be investigated. Blood sampling timepoints: within 1 h before the first dose of JS004, 24 h (±1 h) and 168 h (Day 8, ±8 h) after the start of the first dose, within 1 h before administration in Cycles 2, 4, 6 and 8, at the end of treatment visit (±7 days) and at the safety follow-up visit (if applicable). The blood collection volume will be 2 mL.

## 3.9. Cytokine Study

Dose escalation and dose expansion phases of Part A and Part B:

The effect of JS004 on peripheral blood cytokines (IFN-γ, IL-8, IL-4, IL-6, IL-10, TNF-α) will be investigated. Blood sampling timepoints: within 1 h before the first dose and 24 h (±1 h) after the start of the first dose. The blood collection volume will be 2 mL.

## 3.10. Immune Cell Subtype Study

Dose escalation and dose expansion phases of Part A and Part B:

The effect of JS004 on peripheral blood immune cell subtypes (CD4+, CD3+CD4+/CD3+CD8+, CD20+, CD3-CD20-NKG2A+ cells, etc.) will be investigated. Blood sampling timepoints: within 1 h before the first dose of JS004, 24 h (±1 h) and 168 h (Day 8, ±8 h) after the start of the first dose, within 1 h before administration in Cycles 2, 4, 6 and 8, at the end of treatment visit (±7 days) and at the safety follow-up visit. The blood collection volume will be 2 mL.

## 3.11. Immunogenicity Study

Anti-drug antibody ADA tests ((including the test before the first dose) will be performed for all subjects who have received at least one dose of JS004 in Part A and Part B. If the results are positive, reanalysis is required. All confirmed positive samples will be reported as positive and will continue to be analyzed for the presence of Nab. Anti-toripalimab tests (including the test before the first dose) will be performed for all subjects who have received at least 1 dose of toripalimab in Part B. Positive samples will continue to be analyzed for the presence of Nab.

Immunogenicity (ADA) blood sampling schedule:

Within 1 h before the dose of JS004 in Cycles 1, 2, 4, 6, 8, 12, 16, and every 8 cycles thereafter, at the end of treatment visit (±7 days), at the safety follow-up visit, at the onset of the event of infusion reaction (+5 min), resolution of the reaction (+5 min), and 30 days (± 3 days) after the reaction for any subject. The blood collection volume will be 5 mL.

### 3.12. Biomarker Study (Optional)

Tumor tissue specimens provided at screening may be archived within 2 years before the first dose of the study drug or freshly obtained, with fresh biopsy more preferred than archived tissue. Specimens must be formalin-fixed, paraffin-embedded (FFPE) tumor tissue blocks and be cut into at least 10-14 sections with a thickness of 4-6 μm for staining and detection. Fresh specimens should be collected using a core needle (18 gauge or larger) or by excisional or incisional tumor biopsy. Cell smears without pleural effusion drain centrifugation, bone lesions without soft tissue components or decalcified bone tumor specimens, and tissues for punch biopsy are insufficient for biomarker testing;

Note: If the subject fails to provide the specified amount of slides, the subject may enter the screening period after communicating with the sponsor and obtaining approval from the medical monitor.

Six milliliters of blood will be collected before infusion in Cycle 1, at each imaging assessment (before administration) and at the end of treatment visit for tumor biomarker study, including detection of immune cell surface receptors PD-1, HVEM, CTLA-4, CD112R, TIM-3, ICOS, CX3CR1, CD183 and CD103. Two milliliters of blood will be collected before the first dose of JS004 in Cycle 1 for WES detection of tumor tissue paired blood.

See the Laboratory Manual for specific methods for sample collection and test contents.

## 4. Selection of Subjects

Patients with relapsed/refractory malignant lymphoma are target study population. The informed consent must be signed before participation in the clinical trial.

### 4.1. Inclusion Criteria

Each patient eligible to participate in this study must meet the following criteria:

1. Understand and voluntarily sign an informed consent form;
2. Age 18-70 years (inclusive), male or female;
3. Pathologically confirmed malignant lymphoma;
4. Patients with relapsed/refractory malignant lymphoma (peripheral T-cell lymphoma, Hodgkin lymphoma, follicular lymphoma, etc. are preferred), including 1) those who relapse and progress after autologous stem cell transplantation; 2) those who fail to undergo autologous stem cell transplantation must have failed systemic second-line treatment, including no response (SD or PD), or relapse and progression (patients with peripheral T-cell/NK-cell lymphoma and mediastinal large B-cell lymphoma can be enrolled after failure of first-line treatment); 3) those with B-cell lymphoma must have previously received rituximab;
5. ECOG: 0~1;
6. Expected survival ≥12 weeks;
7. Having at least one measurable lesion that meets the requirements of Lugano 2014 Criteria;
8. Voluntary consent to provide fresh biopsy specimen before treatment or archived specimen within 2 years for patients unable to provide fresh one before treatment;
9. The function of vital organs meets the following requirements (no need for transfusion, blood products, hematopoietic stimulating factors or other drugs to correct blood cell counts within 14 days before the first dose):

- Absolute neutrophil count (ANC) ≥1.5×$10^9$/L;
- Platelets (PL) ≥100×$10^9$/L, PL ≥75×$10^9$/L in patients with bone marrow involvement;
- Hemoglobin (Hb) ≥90 g/L;
- Total bilirubin (TBIL) ≤1.5×ULN; TBIL ≤2×ULN for patients with liver metastases; Direct bilirubin (dBIL) ≤3.0 mg/dL for patients with Gilbert syndrome;
- Alanine aminotransferase (ALT) and aspartate aminotransferase (AST) ≤2.5×ULN; ALT and AST ≤5×ULN for patients with liver metastases;
- Serum creatinine (Cr) ≤1.5×ULN, or calculated creatinine clearance (Cockcroft-Gault formula) ≥50 mL/min;
- International normalized ratio (INR) ≤2.0 and activated partial thromboplastin time (aPTT) ≤1.5×ULN for patients not receiving anticoagulant therapy; A stable dose of anticoagulant drugs for at least 4 weeks without dose adjustment is required for patients receiving anticoagulant therapy (such as low molecular weight heparin or warfarin); QTc interval ≤450 ms for males and ≤470 ms for females calculated according to Fridericia's criteria;

10. Female patients of childbearing potential and male patients whose partners of childbearing potential are required to use a medically approved contraceptive method (such as intrauterine device (IUD), contraceptive pill or condom) during the treatment period and within 3 months after the end of the treatment period of the study; Female patients of childbearing potential must have a negative serum or urine HCG test within 7 days before enrollment in the study and must be non-lactating.

## 4.2. Exclusion Criteria

Patients who met any of the following criteria will be excluded from the study:

1. Patients who are known allergic to the components of large molecular protein preparations, JS004 or toripalimab;
2. Having previously been treated with anti-BTLA or anti-HVEM antibodies;
3. Participation in other clinical studies within 4 weeks before the first dose of JS004, except for an observational (non-interventional) clinical study or the follow-up period of an interventional study;
4. Major surgery (as judged by the investigator) within 4 weeks before the first dose of JS004 or in the recovery period of surgery. Having received anti-tumor chemotherapy (within 6 weeks after the last chemotherapy with nitrosourea or mitomycin), radiotherapy, targeted therapy, immunotherapy or biological therapy within 4 weeks before the first dose of JS004. Having received traditional Chinese medicine or Chinese patent medicine with anti-tumor indications within 2 weeks before the first dose of JS004. Local palliative treatment, such as local surgery or radiotherapy, for isolated lesions is acceptable if no impact on response evaluation is expected;
5. Patients who discontinue prior immunotherapy due to immune-related adverse reactions;
6. Having received immunosuppressive medications within 4 weeks before the first dose of JS004, except for corticosteroid nasal spray, inhalers, or systemic prednisone ≤10 mg/day or equivalent;
7. Having previously received allogeneic bone marrow transplantation or solid organ transplantation;
8. Having received a live or live attenuated vaccine within 30 days before the first dose of JS004;
9. Suffering from two or more malignancies within 5 years before the first dose of JS004, except for early malignancy (carcinoma in situ or stage I tumors) that have been cured (no relapse for 5 years), such as adequately treated carcinoma in situ of the cervix, basal or squamous cell skin cancer;
10. Presence of central nervous system (CNS) involvement that is symptomatic, untreated, or requires continued treatment (including corticosteroids and antiepileptic drugs). Patients who have previously received treatment but are clinically stable for at least 4 weeks before enrollment can be enrolled, excluding patients with evidence of new or expanded metastasis and discontinued steroid therapy;
11. Presence of unrelieved toxicities from prior anti-tumor therapy, i.e., having not resolved to baseline, to NCI-CTCAE 5.0 Grade 0 or 1, or levels described in the inclusion and exclusion criteria. Patients with irreversible toxicities (such as hearing loss) that are reasonably expected not to be aggravated by the study drug can be enrolled upon consultation with the medical monitor;
12. Active autoimmune disease requiring systemic treatment within the past 2 years (e.g., use of disease-modifying agents, corticosteroids, or immunosuppressive agents), including but not limited to systemic lupus erythematosus, multiple sclerosis, rheumatoid arthritis, inflammatory bowel disease, autoimmune thyroid disease, vasculitis, psoriasis, etc.). However, patients with hypothyroidism, hypoadrenalism or hypopituitarism controlled only by hormone replacement therapy, type I diabetes mellitus, psoriasis or vitiligo not requiring systemic treatment can participate in this study;
13. History of anaphylaxis and eczema or asthma uncontrolled by topical corticosteroids;

14. History of primary immunodeficiency;
15. Comorbidities that cannot be controlled by concomitant treatment within 14 days before the first dose of the study drug, including but not limited to, ogoing or active infection requiring systemic anti-infective treatment, unexplained fever >38.5°C (subjects with neoplastic fever can be included at the discretion of the investigator); symptomatic congestive heart failure Grade ≥3 according to New York Heart Association (NYHA) functional classification, poorly controlled hypertension (systolic blood pressure ≥160 mmHg and/or diastolic blood pressure ≥100 mmHg; or pulmonary hypertension, angina unstable, or myocardial infarction, coronary artery bypass grafting or stent implantation and Grade ≥2 ventricular and supraventricular arrhythmias within 6 months before study medication; Cerebrovascular accident (CVA) or transient ischemic attack (TIA) within 6 months before study medication; Active peptic ulcer disease or gastritis;
16. Previous or current interstitial lung disease, obstructive lung disease that severely affects lung function, or current active pulmonary tuberculosis;
17. Patients with associated clinical symptoms (dyspnea, wheezing, abdominal distension, etc.), uncontrolled pleural/abdominal effusion or pericardial effusion requiring repeated drainage;
18. History of active inflammatory bowel disease (such as Crohn's disease or ulcerative colitis);
19. Patients who are known to be human immunodeficiency virus (HIV) positive;
20. Patients with evidence of hepatitis B virus (HBV) or hepatitis C virus (HCV) infection. Patients who are HBsAg positive, those who had HBV infection and are HBcAb positive and HBsAg negative but with HBV DNA copies less than the upper limit of normal in the site after treatment, and patients who had HCV infection and a negative HCV RNA test result at screening are eligible for this study;
21. Female patients who are pregnant or lactating;
22. Patients with rheumatoid arthritis and other arthropathies, Sjogren's syndrome, celiac disease and psoriasis controlled by topical application, as well as those with positive antinuclear antibody (ANA), antithyroid antibody and other serological tests should be evaluated for the involvement of target organs and the need for systemic therapy as judged by the investigator;
23. Any other medical (such as respiratory, metabolic, congenital, endocrine or central nervous system diseases, etc.), psychiatric or social factors that may affect the rights and interests, safety, compliance, ability to sign the informed consent, as well as interpretation of study results as judged by the investigator;
24. Subjects who have a history of psychotropic drug abuse and are unable to withdraw or have mental disorders.

#### 4.2.1. Lifestyle Requirements

##### 4.2.1.1. Contraception

After consultation with the investigator or his/her designee, the subjects will select an appropriate contraceptive method from the following and be educated on how to correctly and continuously use the contraceptive methods. In addition, the subjects should be instructed to notify the investigator immediately if they discontinued the selected contraceptive method or if they or their partner have a suspected or confirmed pregnancy.

Highly effective contraceptive methods refer to contraceptive methods with an annual failure rate of less than 1% if continuously and correctly used alone or in combination, including the following types:

1. Commonly used hormonal contraceptive methods associated with inhibition of ovulation (e.g., oral, insertion, injection, implantation, transdermal), provided that they are confirmed to be effective for the female subjects or the partners of male subjects who have been using these methods for an adequate period of time, and will be continuously and correctly used during the study treatment.
2. With properly placed IUD.
3. Male/female condom in combination with topical spermicides (i.e., foam, gel, film, cream or suppository).
4. Sterilized males by vasectomy.
5. Bilateral tubal ligation/bilateral tubal resection or bilateral fallopian tube occlusion (occlusion has been confirmed effective by relevant instruments).

### 4.3. Subject’s Withdrawal

#### 4.3.1. Criteria for Subject's Withdrawal

Under any of the following circumstances, subjects must discontinue the study drug treatment:

1. Withdrawal of informed consent by the subject;
2. Clear evidence of radiographic progression;
3. Intolerable treatment toxicity;
4. Become pregnant during the study;
5. Loss to follow-up or death;
6. Treatment with JS004 for 24 months;
7. Initiation of another anti-tumor therapy;
8. Obvious inability of the subject to comply with the study procedures as judged by the investigator and/or other conditions that necessitate withdrawal from the study;
9. Study termination required by the sponsor, investigators or regulatory authorities.

#### 4.3.2. Procedures for Subject‘’s Withdrawal from the Study

It is important to complete all tests at the end of treatment visit specified in the protocol, complete the safety follow-up visit, and fully record adverse events (AEs) and their outcomes. The investigator may suggest or provide a new or alternative therapy to subjects base on their actual conditions. Patients who withdraw for reasons other than progressive disease need to continue the response follow-up and undergo radiologic assessment until the start of new anti-tumor therapy, progressive disease, or death, etc.

If a subject refuses to come to the site for further visits, the survival status should continue to be followed up unless he/she withdraws informed consent. In this case, no further study evaluation should be conducted, and no further information should be collected. The sponsor may retain and continue to use all information before the subject withdraws their informed consent unless the subject also requests that the collected information be withdrawn.

#### 4.3.3. Replacement of Early Withdrawn Subjects

Subjects who withdraw before completing the treatment in Cycle 1 for reasons other than a DLT will be replaced. If no DLT occurs during the DLT observation period, the subject will not be included in the final analysis set for this dose group or overall DLT, but will only be included in the safety analysis set. One additional subject will be included in the same dose group for replacement.

#### 4.3.4. Identification of Subjects

All subjects who sign the informed consent in this study will receive a subject code, which is a unique 5-digit number, such as 01001; The numbering rule is that the first 2 digits are the site number, such as 01, and the last 3 digits are 001, 002, 003 and so on in the screening order.

### 4.4. Early Termination or Suspension of Study

The study may be terminated or suspended prematurely for adequate reasons. The sponsor should submit a written notice stating the reason for premature termination or suspension to the investigators, the National Medical Products Administration (NMPA), and relevant authorities if this study is prematurely terminated or suspended. The principal investigators must immediately report it to the Ethics Committee and provide appropriate reasons.

The termination criteria of this study include but are not limited to the following:

1. The study brings unexpected, significant, or unacceptable risks to subjects;
2. The protocol is found with major mistakes during the study;
3. The study drug/treatment is ineffective, or it is meaningless to continue the study;
4. The sponsor decides to discontinue the study due to serious lag in enrollment or frequent protocol deviations etc.

# 5. Study Drug

## 5.1. Drug Dispensation

A subject number will be automatically assigned after the subject has signed a written informed consent form. The number will be used in all eCRFs and study documents. The subject will be formally enrolled in the study after the screening process.

No study drug will be received by any subject until the following information is confirmed in written form.

- Confirmation of subject enrollment;
- Confirmation of the dose level for this subject;
- Permission to dose.

## 5.2. Supply of study drug

### 5.2.1. Dosage form and Strength

JS004 drug product is supplied in 6 mL Type I glass vials. Each vial contains a clear, colorless or yellowish solution, with a volume of 5 mL. The vials are sealed with a 20 mm rubber stopper and a 20 mm plastic aluminum seal cap. Each vial contains 100.0 mg of JS004 protein, 3.9 mg of histidine, 15.7 mg of L-histidine hydrochloride, 14.6 mg of sodium chloride, 264.9 mg of α, α-trehalose dihydrate, and 1.0 mg of Polysorbate 80 (PS80). The pH is approximately 5.5.

Toripalimab injection (JS001) is supplied as a clear, colorless or yellowish liquid with slight opalescence. The strength is 6 ml/vial. Each vial contains toripalimab (240.00 mg), citric acid monohydrate (3.06 mg), sodium citrate dihydrate (31.80 mg), sodium chloride (17.52 mg), mannitol (150.00 mg) and Polysorbate 80 (1.20 mg). The pH is 6.0±0.5.

### 5.2.2. Preparation and Dispensation of study drug

A designated person is responsible for the management, dispensing, and return of the study drug in this study. The investigator must ensure that all study drugs are used only for subjects participating in the study, and their dosage and dose should follow the study protocol. The remaining drugs should be returned to the sponsor. The study drugs should not be transferred to anyone other than clinical trial participants.

The monitor is responsible for monitoring the supply, use, storage of the study drug and handling of remaining drugs.

## 5.3. Dosing

### 5.3.1. Dosing Regimen for JS004 Monotherapy

JS004 is administered by intravenous infusion once every 3 weeks. The first infusion duration of JS004 is at least 60 min. If the patient has mild allergic reactions, such as dizziness, chills, and rash during infusion, the infusion rate can be appropriately reduced and the infusion time can be extended accordingly. The longest infusion time should no less than 2 h. If the infusion of the first dose is well tolerated, the second dose may be infused in a shortened period of at least 30 min. All subsequent infusions can be administered over at least 30 min if the infusion over 30 min is well tolerated. All information on administration must be recorded on the medication management page of the eCRF.

Prophylactic medication should not be used before the first dose. Prophylactic medications such as glucocorticoids and acetaminophen can be given according to the occurrence of infusion-related adverse events after the first dose. During infusion, subjects should be closely monitored so that infusion-related adverse events can be promptly found and managed.

### 5.3.2. Dosing Regimen for JS004 in Combination with Toripalimab

JS004 in combination with toripalimab will be administered by intravenous drip, Q3W.

For subjects in the phase of JS004 in combination with toripalimab, JS004 is administered first, followed by toripalimab at an interval of approximately 1 h.

The first infusion time of JS004 is at least 60 min. If the patient has mild allergic reactions such as dizziness, chills, and rash during the infusion, the infusion rate can be appropriately reduced and the infusion time can be extended accordingly. The longest infusion time should no less than 2 h. If the infusion of the first dose is well tolerated, the second dose may be infused in a shortened period of 30 min. All subsequent infusions can be administered over 30 min if the infusion over 30 min is well tolerated. All information on administration must be recorded on the medication management page of the eCRF.

The first intravenous infusion of toripalimab is at least 60 min. If the infusion of the first dose is well tolerated, the second dose may be infused in a shortened period of at least 30 min. All subsequent infusions can be administered over at least 30 min if the infusion over 30 min is well tolerated.

Prophylactic medication should not be used before the first dose. Prophylactic medications such as glucocorticoids can be given according to the occurrence of infusion-related adverse events after the first dose. During infusion, subjects should be closely monitored so that infusion-related adverse events can be promptly found and managed.

All doses must be given at the clinical site under the supervision of appropriately trained staff. Blood samples cannot be collected from the arm on the infusion side until at least 24 hours post-dose. If infused via a central venous catheter, blood samples for PK/PD/immunogenicity studies should be collected from a peripheral vein.

### 5.3.3. Dose Adjustment

In this study, no dose modification is allowed, but dose delays are allowed.

During the DLT observation period (21 days) of the dose escalation phase, the maximum delay of the investigational product should not exceed 7 days. A maximum dose delay of 42 days is allowed for JS004 and toripalimab during the treatment period thereafter (calculated based on the scheduled dosing date).

Dose interruptions for reasons other than toxicity, such as surgical procedures, may be allowed after being approved by the sponsor or the CRO's medical monitor, and the acceptable duration of dose interruptions is subject to agreement between the medical monitor and the investigator. If toripalimab is permanently discontinued due to intolerable toxicity during the combination treatment period, the subject may continue JS004 monotherapy if he/she can benefit from JS004 at the discretion of the investigator. If JS004 is permanently discontinued due to intolerable toxicity, the subject will be advised to discontinue the investigational product.

#### 5.3.3.1. Criteria for Dose Interruption Due to Treatment-emergent Adverse Events (TEAEs)

Study drug should be interrupted in case of any of the following adverse events during the treatment:

- Grade 2 pulmonitis;
- Grade 2 colitis;
- Grade 2 nephritis;
- Grade ≥3 severe skin adverse reactions, Stevens-Johnson Syndrome (SJS) or toxic epidermal necrolysis (TEN);
- Grade 3 or 4 endocrinopathy;
- AST or ALT >3×ULN and ≤5×ULN, or total bilirubin >1.5×ULN and ≤3×ULN;
- Patients with liver metastases have Grade 2 AST or ALT at baseline, with AST or ALT increasing ≥50% from baseline, which persisted for less than 1 week;
- Other Grade 2 or 3 TEAEs, except for Grade 2 drug-related asthenia or laboratory abnormalities not requiring treatment interruption.

#### 5.3.3.2. Criteria for Permanent Discontinuation

The study drug should be discontinued in case of any of the following adverse events during the treatment:

- Grade ≥3 drug-related pulmonitis, increased creatinine, or neuropathy;
- Grade ≥3 colitis;
- Any Grade 4 drug-related AE or laboratory abnormality, with the following exceptions:
    - Isolated Grade 4 amylase or lipase abnormalities without any symptoms or clinical manifestations of pancreatitis, which decrease to less than grade 4 within 1 week of occurrence;
    - Endocrinopathy controlled after treatment with hormone replacement therapy;
- Corticosteroids should not be reduced to ≤15 mg/day prednisone or other equivalent doses within 12 weeks (except for endocrine diseases controlled by hormone replacement therapy);
- AST or ALT >5×ULN or total bilirubin >3×ULN;

- Patients with liver metastases who have Grade 2 AST or ALT at baseline, with AST or ALT increasing ≥50% from baseline, and persisted for at least 1 week;
- Persistent drug-related AEs that do not return to Grade 0-1 or baseline within 12 weeks, unless otherwise agreed between the sponsor and the investigator based on evidence of clinical benefit;
- Recurrence of any recurrent Grade 2 pulmonitis or Grade 3 AEs;
- Grade ≥3 myocarditis;
- Grade ≥3 Stevens-Johnson Syndrome (SJS) or toxic epidermal necrolysis (TEN).
- Any AE, laboratory abnormality, or other condition that, at the discretion of the investigator, will pose a significant clinical risk to the patient with continued use of JS004 and/or JS001.

After discontinuation of treatment, subjects should complete the EOT visit as described in the study flow chart.

### 5.3.4. Criteria for Dose Resumption

If the drug-related AE resolves to Grade ≤1 or baseline, the subject may resume the study drug. Treatment with study drug may also be resumed if:

- Grade 2 asthenia;
- Subjects who have not experienced Grade 3 drug-related skin toxicity, but only Grade 2 skin toxicity;
- Drug-related pulmonary toxicity, diarrhoea, or colitis must resolve to baseline;
- Drug-related endocrinopathy that can be adequately controlled with hormone replacement therapy only.

## 5.4. Storage of Study Drug

JS004 drug product should be stored at 2-8°C and protected from light. JS004 drug product should be diluted with 0.9% sodium chloride for injection to concentrations ranging from 0.5 to 10.0 mg/mL before use, and then administered intravenously. Diluted JS004 drug product should be infused within 4 h if stored at room temperature.

JS001 drug product should be stored at 2-8°C and protected from light. JS001 drug product should be diluted with 0.9% sodium chloride for injection to concentrations ranging from 1 to 3 mg/mL before use, and then administered intravenously. Diluted JS001 drug product should be infused within 8 h if stored at room temperature.

Handling instructions for JS004 and JS001 will be provided in a separate Pharmacy Manual. The site will be responsible for the infusion of the study drug to subjects and documenting details of receipt, storage, assembly, handling, and return according to the Good Clinical Practice.

## 5.5. Concomitant Medications

All concomitant medications taken by the subjects during the study from 28 days before voluntarily signing the informed consent form should be recorded in the source documents and appropriate pages of the eCRF, The name, dosage, duration and indications of each medication should be noted. In addition, non-pharmacological interventions (except vitamins) and any changes in concomitant medications or other interventions should also be recorded in the source document and eCRF.

### 5.5.1. Permitted Medications

Any medication, other than those excluded by the clinical protocol, that is considered necessary to protect the subject's rights and interests and does not interfere with the investigational product may be administered according to the medical judgement on the subject. All treatments and medications used concomitantly within 28 days before signing the informed consent form and during the study should be recorded in the original medical records as specified in GCP.

Permitted medications include analgesics, antinauseants, antihistamines, diuretics, anxiolytics, and analgesics (including anesthetics).

Prophylaxis with H1 blockers (diphenhydramine 50 mg IV or equivalent) and acetaminophen or paracetamol (500 to 650 mg oral or IV) is allowed 30 to 60 minutes before each JS004 infusion, as necessary.

During the study, the subject should be given the best supportive care. Clinical concomitant diseases and various adverse reactions, especially immune-related adverse reactions, should be closely observed and active symptomatic treatment should be given, if necessary, and the medications used should be recorded and explained in the original medical records.

Subjects are allowed to use bisphosphonates for bone metastases. Subjects will be allowed to use palliative radiotherapy to small areas (radiotherapy area must be <5% of bone marrow area, see the figure in Annex 6 for percent of bone marrow content) if bone metastases are not adequately controlled by systemic therapy or local analgesia. However, it is required that these lesions are known pre-existing at the time of enrollment and that the investigator is required to clearly clarify that the use of palliative radiotherapy does not imply disease progression. Due to the lack of information on the interaction between JS004 and radiation therapy, treatment with JS004 will be interrupted during palliative radiation therapy. The study drug will be resumed after completion of radiation therapy.

### 5.5.2. Prohibited Medications

For details of medications that should not be used within a specific time before enrollment, see the inclusion criteria. The following medications are prohibited during the study:

- Any other anti-tumor therapy, including traditional Chinese medicine preparations and Chinese herbal medicine treatments with anti-tumor indications (including but not limited to the list of drugs in Annex 8);

- Immunotherapy, immunosuppressive agents (such as systemic corticosteroids prednisone >15 mg/day or other equivalent drugs), methotrexate, azathioprine and tumor necrosis factor-α (TNF-α) retardant are prohibited. However, short-term administration of systemic steroid therapies are allowed, such as the treatment for allergic reactions or immune/infusion-related AEs;
- Granulocyte colony-stimulating factor, erythropoietin, denosumab, darbepoetin alfa, etc. are prohibited during the DLT observation period. Such medications can be used beyond the DLT observation period at the discretion of the investigator;
- Prophylactic medications for diarrhoea, nausea and vomiting during the DLT observation period are prohibited. Prophylactic medications can be used beyond the DLT observation period at the discretion of the investigator;
- Live or live attenuated vaccines are prohibited.

## 6. Study Procedures

Both Part A and Part B of the study are generally divided into three phases, dose escalation, dose expansion, and indication expansion. Each phase includes a screening period, a study drug treatment period, and a follow-up period. Each subject will only participate in one phase of the study. Subjects must read and sign an Ethics Committee-approved informed consent form before the start of all study procedures. All study procedures must be completed at the timepoint and time window defined in the protocol, as shown in Flow Chart 1, Table 2, Table 3.

### 6.1. Screening Period (Day -28 to Day 1)

Unless otherwise specified, the following screening procedures must be completed within 28 days before the first dose of JS004, and all laboratory tests must be completed within 7 days before the first dose of JS004.

Examinations are not required after signing the informed consent if these examinations have already been performed as part of routine clinical diagnosis at the study site prior to the subjects signing the informed consent form, these examinations meet the specified examination requirements in the protocol, and the time elapsed since the first administration of the study drug does not exceed the specified screening period. Abnormal screening results related to the eligibility criteria are only repeated once within the screening period. The last result before the first study drug dose is used to decide whether a patient is eligible.

[Informed consent signing]: Signed informed consent must be obtained from the subjects before the start of any study procedures.

[Demographic data]: Sex, birth year and month, nationality, height and weight, etc.;

[Previous tumor history]: It includes pathological diagnosis and clinical diagnosis:

- Tumor diagnosis: date of first tumor diagnosis, histological type, location of primary and metastatic lesions, pathological stage, and clinical stage;
- Surgical history: surgical history of primary lesions, metastatic lesions;

- Medical treatment history (including neo/adjuvant therapy): history of systemic chemotherapy, targeted drug therapy, immune checkpoint drug therapy, local chemotherapy, etc.;
- Radiotherapy history: radiotherapy method, time, dose, site (systemic/local), etc.;
- Gene mutation test results (if applicable): the presence of gene mutation, mutation sites, test time, etc.;
- The date of progressive disease or recurrence after the last systemic treatment;

[Concomitant diseases]: Concomitant diseases and relevant treatment history (such as diabetes, hypertension and other chronic diseases);

[Vital signs]: Body temperature, heart rate, respiratory rate and blood pressure should be measured after at least 5 min of comfortable rest (the start and end times of rest should be recorded);

[Physical examination]: General conditions, head and face, skin, lymph nodes, eyes (sclera, pupil), ears, nose, throat, respiratory system, cardiovascular system, abdomen (including liver and spleen), reproductive-urinary system, musculoskeletal system, nervous system, mental status, etc.;

[ECOG performance status score]: See Annex 2 for the scoring criteria;

[Hematology]: White blood cell count (WBC), neutrophil count (ANC), red blood cell count (RBC), hemoglobin (Hb), platelet count (PLT), lymphocytes, monocytes, eosinophils, basophils, etc.;

[Biochemistry]: Including total bilirubin (TBIL), direct bilirubin (dBIL), alanine aminotransferase (ALT), aspartate aminotransferase (AST), gamma-glutamyltranspeptidase (γ-GT), alkaline phosphatase (ALP), albumin (ALB), total protein (TP), lactate dehydrogenase (LDH), blood urea nitrogen (BUN), creatinine (Cr), uric acid, potassium ($K^+$), sodium ($Na^+$), chloride ($Cl^-$), calcium ($Ca^{2+}$), magnesium ($Mg^{2+}$), phosphorus or phosphate, amylase, lipase and fasting blood glucose, etc. If necessary, other tests might be added by the investigator;

[Coagulation function]: Including thrombin time (TT), prothrombin time (PT), activated partial thromboplastin time (aPTT) and international normalized ratio (INR), etc.;

[Urinalysis]: Urine specific gravity, PH, urine glucose, protein urine (if protein urine ≥2+, 24-hour protein urine quantification must be performed; the subject can be enrolled if 24-hour protein urine ≤2 g), ketone bodies, red blood cells urine, white blood cells urine, etc.;

[Thyroid function]: Including tri-iodothyronine free (FT3), free T4 (FT4) and thyroid stimulating hormone (TSH), etc.;

[Autoantibody]: Antinuclear antibodies, anti-double-stranded DNA (dsDNA) antibodies, anti-thyroglobulin antibodies, etc.;

[HIV/HBV/HCV]: Including human immunodeficiency virus (HIV), antibody hepatitis B virus (HBV), and hepatitis C virus (HCV) tests. Hepatitis B test includes hepatitis B surface antigen (HBsAg), hepatitis B surface antibody (HBsAb), hepatitis B core antibody (HBcAb), hepatitis B e antigen (HBeAg), hepatitis B e antibody (HBeAb). If HBsAg and/or HBcAb are positive, HBV DNA test should be performed further. If the anti-HCV antibody is positive for the subject, HCV RNA testing is required;

[Pregnancy test]: Only applicable to women of childbearing age. The test should be performed within 7 days before the first dose; A serum pregnancy test should be performed if a urine pregnancy test is inconclusive. Conclusions should be based on serum pregnancy tests;

[PK/PD/cytokines/immune cell subtype/immunogenicity/biomarker blood sampling]: See PK/PD/Cytokine/Immune Cell Subtype/Immunogenicity/Biomarker Studies for details;

[12-lead ECG]: If abnormal, echocardiography and other necessary examinations should be performed at the discretion of the investigator;

[Pulmonary function test]: Subjects with obstructive pulmonary disease must have a baseline pulmonary function test. If the percentage of FEV1 accounting for the predicted is ≥50%, the subjects may be enrolled in the study, and other subjects may have a pulmonary function test as clinically indicated.

[Imaging examination]: CT or MRI of brain, chest, abdomen, pelvis and any other known lesions. FDG-PET examination. A bone scan is required when there is known or clinical suspicion of metastases to bone;

[Bone marrow aspiration biopsy]: Baseline bone marrow aspiration biopsy is performed for all patients, except for Hodgkin lymphoma, but the study drug can be started before the bone marrow biopsy results are obtained;

[IPI assessment]: See Annex 3 for assessment criteria;

[B symptom assessment]: B symptoms include unexplained fever >38°C, night sweats, weight decrease >10% within 6 months before assessment;

[Tumor tissue sample collection]: Fresh or archived (within 2 years) tissue blocks or pathological sections will be collected from subjects. See Section 3.12 for sample requirements in detail;

[Concomitant medications]: See the section of Concomitant Medications for details;

[Adverse events]: See the section of Adverse Events for details;

[Enrollment]: Subjects must meet all inclusion criteria and none of the exclusion criteria to be included in the study.

## 6.2. Treatment Period

The treatment period starts from Day 1 of the dosing of the study drug and continues until the end of treatment (EOT) visit.

According to study progress, subjects will be assigned to the designated dose group to receive the corresponding treatment:

Subjects in the dose escalation study of Part A will first undergo DLT evaluation. If the subject is tolerable as judged by the investigator after completing the DLT evaluation, the subject will continue to be administered at the same dose until progressive disease, intolerable toxicity, treatment with JS004 (since the first dose) for more than 2 years or other reasons specified in the protocol. In the dose expansion study, DLTs that possibly occur will be used as a reference for subsequent clinical studies int terms of dosing design and safety, and the duration of treatment with JS004 (since the first dose) is up to 2 years.

Subjects in the dose escalation study of Part B will first undergo DLT evaluation (21 days after the first dose). JS004 and toripalimab will be continued at the same dose after the completion of DLT evaluation. The duration of treatment with JS004 and/or toripalimab (since the first dose) is up to 2 years.

In the dose escalation phase, Cycle 1 (21 days) is the DLT observation period. Subjects should follow the relevant ward management system and arrange the admission time according to the local management of phase I ward and the protocol requirements. Subjects will receive the first infusion of JS004 on Cycle 1 Day 1 over at least 60 min, followed by close observation for acute allergic reactions (at least 6 h of observation) within 24 h after infusion. In case of any infusion related reaction (IRR), clinical treatment should be performed based on the medical practice and relevant guidelines of the hospital (see 8.5.1 Infusion Reactions). If no IRR during the first infusion, the subsequent infusion time can be shortened to at least 30 min. The infusion will be performed on Day 1 of each cycle and observed for at least 1 h after the infusion (3 weeks as a cycle). The infusion time is up to 2 h. The next scheduled dose of the study drug should be determined based on the date of the last dose.

DLTs or delayed DLTs that may occur in subjects in the dose expansion study will not be used as the basis for dose escalation, but as a reference for the selection of the recommended dose for expansion. All subjects in Part B will receive protocol-specified doses of JS004 and toripalimab until progressive disease, intolerable toxicity, treatment with JS004 and/or toripalimab (since the first dose) for more than 2 years, or other reasons as specified in the protocol. The other study procedures in the second and third phases are the same as those in the first phase.

The following activities will be performed on Cycle 1 (21 days):

C1D1: [Vital signs], [Height/weight], [Physical examination], [B symptom assessment], [Hematology], [Biochemistry], [Urinalysis], [PK/PD/immunogenicity/biomarker blood sampling], [12-lead ECGs], [JS004 intravenous drip], [Adverse events], and [Concomitant medications]. Laboratory tests performed within 72 hours before the first dose should not be repeated on C1D1;

C1D2 (±1 h): [Vital signs], [Physical examination], [Blood sampling for PK/PD/cytokine/immune cell subtype studies], [12-Lead ECG], [Adverse events] and [Concomitant medications];

C1D3 (±2 h): [Vital signs], [Physical examination], [12-lead ECG], [PK], [Adverse events] and [Concomitant medications];

C1D8 (±8 h): [Vital signs], [Physical examination], [Hematology], [Blood chemistry], [Urinalysis], [PK/PD/immune cell subtype], [12-lead ECGs], [Adverse events], and [Concomitant medications];

C1D15 (±2 h): [Vital signs], [Physical examination], [Hematology], [Blood chemistry], [Urinalysis], [Coagulation function], [Thyroid function], [PK], [12-lead ECG], [Adverse events], and [Concomitant medications];

The following main procedure items will be performed in Cycle 2 and subsequent cycles (21-day as a cycle):

CnD1 (± 3 days): [vital signs], [height/weight], [physical examination], [ECOG performance status score], [B symptom assessment], [Hematology], [Biochemistry], [Coagulation function], [Urinalysis], [Thyroid function], [Pregnancy test], [PK/PD/immune cell subtype/immunogenicity/biomarker blood sampling], [12-lead ECG], [Concomitant medication] and [Adverse events];

[Imaging examination] Tumor imaging assessments will be performed every 9 weeks (±7 days) after the first dose of the investigational product until any of the following occurs: initiation of a new anti-tumor therapy, progressive disease, withdrawal of consent, study discontinuation, loss to follow-up or death. Imaging examinations should be repeated for confirmation for subjects who experience the first radiographic progressive disease and meet the requirements of Section 7.3 within 4-8 weeks (±7 days). The imaging methods during the treatment period are consistent with those at baseline. FDG-PET examination should be performed within 4 weeks when complete response (CR) is assessed by imaging such as CT or MRI. Whole body bone scan can be performed as clinically indicated.

[Bone marrow aspirate biopsy]: For subjects with baseline confirmed bone marrow involvement who achieve radiographic CR during the trial, a second bone marrow aspirate biopsy is required to confirm CR status within 2 weeks after the completion of imaging (FDG-PET confirmation for Hodgkin lymphoma only is required). Bone marrow assessment is not required after a negative bone marrow aspirate biopsy.

[B symptom assessment] Assessments will be performed on Day 1 of each cycle until discontinuation of study treatment. In addition, assessments should also be performed when the subject first achieves PR or CR; If a subject achieves PR prior to CR, re-assessment is required at the time of CR;

See the sections of PK/PD/Cytokine/Immune Cell Subtype/Immunogenicity/Biomarker Study Analyses for details of other PK/PD/immune cell subtype/immunogenicity/biomarker blood sampling timepoints in each cycle.

## 6.3. End of treatment

The subject must discontinue the treatment with the investigational product in cases of the following: withdrawal of consent, presence of clear evidence of radiographic progression, intolerance of treatment toxicity, occurrence of pregnancy event, loss to follow-up or death, treatment with JS004 or/and toripalimab for 24 months, initiation of another anti-tumor therapy, obvious inability of the subject to comply with the study as judged by the investigator, or other conditions that necessitate withdrawal from the study.

In addition to loss to follow-up, death, or withdrawal of consent, subjects who discontinue treatment for any reason should have an end of treatment (EOT) visit. The date of the end of treatment is the date when the investigator makes the end of treatment decision, and the date of the end of treatment visit is the end of treatment + 7 days. The following examinations will be performed:

[Vital signs], [Physical examination], [ECOG performance status score], [B symptom assessment], [Hematology], [Biochemistry], [Coagulation function], [Urinalysis], [Thyroid function], [Pregnancy test], [Blood sampling for PK/PD/immune cell subtype/immunogenicity/biomarker studies], [12-Lead ECG], [Imaging examination], [Concomitant medications] and [Adverse events].

The end of treatment visit does not need to be repeated if the subject has undergone imaging examination and response evaluation within 4 weeks before the end of treatment visit and laboratory tests within 7 days.

Subjects will be informed that the study drug will no longer be provided after the end of treatment. They should consult their treating physician for the subsequent treatment.

## 6.4. Follow-up and Unscheduled Visits

Subjects will enter the follow-up period after the end of treatment of the study drug.

The safety follow-up will be ended 90 days after the last dose of the study drug or initiation of a new anti-tumor therapy, whichever occurs first. Subjects should return to the site for the protocol-specified safety follow-up visit 30 days (±7 days) after the last dose of the study drug or before initiating a new anti-tumor therapy. This visit does not have to be repeated if it is within the time window of the end of treatment visit. Severe delayed immune-related adverse events will be monitored for 30 to 90 days after the last dose of the study drug. If a delayed immune-related adverse event is suspected, the subject will be asked to visit the hospital within 5 calendar days after being informed. The safety information, including AE outcome, new AE/SAE, AE of special interest and concomitant medications, will be collected through telephone follow-up at 60 days (±7 days) after the last dose of JS004. Subjects should return to the hospital for all safety examinations at 90 days (±7 days) after the last dose of the study drug as far as possible. For those who fail to return to the hospital, the safety information, including AE outcome, new AE/SAE, AE of special interest, concomitant medications, etc., should be collected through telephone follow-up. Unresolved adverse events at the time of visit should be monitored at least every 4 weeks until resolution to Grade 0 or 1, stabilization, or withdrawal of consent, whichever occurs first.

Survival data and subsequent information on anti-tumor therapy will be collected every 3 months (± 7 days). Subjects who have not experienced radiographic progression at the end of treatment should continue to undergo imaging examination and response evaluation at a frequency of every 9 weeks until progressive disease, withdrawal of consent, loss to follow-up, death, or study termination.

In the subject's interest, the investigator may arrange unscheduled visits and tests and record the information in the original data and the unscheduled visit page of the eCRF. If subjects discontinue treatment during an unscheduled visit, the corresponding tests and records should be completed according to the end of treatment requirements instead of recording it as an unscheduled visit.

# 7. Assessment

Every effort should be made to ensure that protocol-required visits and tests are performed as scheduled. In case of any deviation from the study plan beyond the control of the investigator, the investigator should take all necessary measures to protect the safety and interests of subjects. When a test required by the protocol is not feasible, the investigator should record the reasons and state what corrective and preventive actions can be taken to ensure that the clinical trial can be carried out according to the protocol requirements. In addition, the study team should be informed of any unexpected condition.

## 7.1. Safety Assessment

The safety assessment includes collecting AEs, serious adverse events (SAEs), vital signs and physical examination, 12-lead ECGs, laboratory tests, including pregnancy tests, and concomitant therapies.

### 7.1.1. Adverse event assessment

Adverse events (AEs) assessment includes type, incidence, severity (graded according to NCI CTCAE v5.0), onset and end time, serious adverse events and causal relationship, and outcome.

AEs occurring during the study, including signs and symptoms at screening, will be recorded on the AE page of the eCRF.

### 7.1.2. Clinical Laboratory Assessment

Before starting the study, the investigator will provide copies of all laboratory certificates and reference ranges for all laboratory tests to the sponsor (or designee).

***Hematology:*** including red blood cells, hemoglobin, platelets, white blood cells, absolute neutrophils, lymphocytes, monocytes, eosinophils, and basophils, etc. The tests should be performed at screening within 7 days before Day 1 of Cycle 1, within 72 h before infusion on Day 1 of Cycle 1, on Day 8 and Day 15 during the DLT period, within 72 h before Day 1 of Cycle 2 and each subsequent cycle, at the end of treatment (±7 days), at the safety follow-up visit, and as clinically indicated. Cycle 1 Day 1 assessment does not need to be repeated if the screening assessments are performed within 72 hours before C1D1.

***Biochemistry:*** including ALT, AST, gamma-glutamine transferase (γ-GT), ALP, $K^+$, $Na^+$, Cl-, $Ca^{2+}$, $Mg^{2+}$, TBIL, dBIL, BUN/Urea, creatinine, uric acid, glucose (fasting), protein total, albumin, phosphorus or phosphate, amylase and lipase, LDH, and TSH. The tests should be performed at screening within 7 days before Day 1 of Cycle 1, within 72 h before infusion on Day 1 of Cycle 1, on Day 8 and Day 15 during the DLT period, within 72 h before Day 1 of Cycle 2 and each subsequent cycle, at the end of treatment (±7 days), at the safety follow-up visit, and as clinically indicated. Cycle 1 Day 1 assessment does not need to be repeated if the screening assessments are performed within 72 hours before C1D1. The tests should be performed before the dose.

***Coagulation tests:*** including TT, PT, aPTT, and INR. The tests should be assessed at screening within 7 days before Day 1 of Cycle 1, on Day 15 during the DLT period, within 72 h before Day 1 of Cycle 2 and every subsequent cycle, at the end of treatment (±7 days), at the safety follow-up visit, and as clinically indicated.

***Thyroid function test:*** including serum tri-iodothyronine free (FT3), free T4 (FT4), and thyroid-stimulating hormone (TSH), etc. The tests should be performed at screening, on Day 15 during the DLT observation period, within 7 days before the dose of the study drug from Cycle 2, at the end of treatment (±7 days), at the safety follow-up visit, and as clinically indicated.

***Urinalysis:*** The test should be performed with urine dipsticks at screening within 7 days before Day 1 of Cycle 1, within 72 h before infusion on Day 1 of Cycle 1, on Day 8 and 15 during the DLT period, within 72 h before Day 1 of Cycle 2 and each subsequent cycle, at the end of treatment (±7 days), at the safety follow-up visit, and as clinically indicated. Cycle 1 Day 1 assessment does not need to be repeated if the screening assessments are performed within 72 hours before C1D1. Microscopic analysis may be performed as clinically indicated. If urine protein is ≥2+ on urine dipsticks, urine samples will be collected to calculate urine protein/creatinine ratio (UP/CR) or 24-hour urine will be collected. Subjects with 24-hour urine protein ≤2 g can be included in the study.

***Pregnancy test (serum/urine):*** For female subjects of childbearing potential, a serum or urine pregnancy test with a sensitivity of at least 25 mIU/mL should be performed in a qualified laboratory 7 days before the first dose of the study drug. The blood pregnancy test will be regularly performed at screening, within 72 h before each study drug infusion,at the end of treatment (± 7 days), and at the safety follow-up visit, and additionally in the absence of a menstrual cycle or when pregnancy is suspected. Additional pregnancy tests may also be performed if required by the Institutional Review Board/Ethics Committee (IRB/EC) or required by local regulations. If the urine pregnancy test is inconclusive, an additional serum pregnancy test should be performed. Conclusions should be based on serum pregnancy tests. Subjects who are determined to have a positive pregnancy test should not be dosed. See Section 8.2.6 for the reporting of pregnancy events in detail.

***Autoantibody:*** Hematology includes antinuclear antibodies, anti-double-stranded DNA (anti-dsDNA) antibodies, and anti-thyroglobulin antibodies. These tests will be performed at screening and as clinically indicated.

### 7.1.3. Vital Signs

Vital signs include temperature, pulse, respiratory rate, and blood pressure. Vital signs will be measured and recorded after the subject has rested comfortably for at least 5 minutes; Blood pressure will be measured using a validated automated blood pressure monitor. If possible, measurements from the same arm should be recorded using the same type of blood pressure recorder.

Vital signs should be measured at screening, within 1 h before infusion, every 15 min (±5 min) during infusion, at the EOI (+5 min) and 1 h (±10 min) after the EOI on C1D1, on Days 2, 3, 8, and 15 during the DLT period, and as needed. In subsequent cycles (Day 1 only), vital signs will be measured within 1 h before infusion, every 15 min (±5 min) during infusion, at the EOI (+5 min), at the end of treatment (±7 days), at the safety follow-up visit, and as clinically indicated. See the Timetable of Events (Sampling Schedule for PK/PD/Cytokine/Immune Cell Subtype Studies) for details.

### 7.1.4. History

A complete medical history will include an evaluation (previous and current) of the following systems and/or diseases: general, head and neck, eyes, ears, nose and throat, chest/respiratory, cardiac/cardiovascular, gastrointestinal/liver, urogenital, musculoskeletal/extremities, dermatological, neurological/psychiatric, endocrine/metabolic, hematologic/lymphatic, allergic/drug allergy, previous surgical history, drug abuse, or other diseases or conditions.

Medical history should include prior medications and treatments for initial diagnosis, including chemotherapy, radiation therapy, surgery, adjuvant therapy, molecularly targeted agents (± chemotherapy), and immune checkpoint inhibitors (e.g., anti-CTLA-4 antibodies and anti-PD-1/PD-L1 antibodies). The exact time of the last prior anticancer therapy before the first dose of the study drug must be recorded.

### 7.1.5. Physical Examination

Any clinically significant changes in the physical examination, once confirmed, will be recorded as an AE or treatment-emergent adverse event (TEAE).

The physical examination will be performed by physicians, and include general appearance, height (at screening) and weight (at screening and each pre-dose calculation), abdomen, head and neck, eyes, ears, nose, throat, chest/respiratory system, heart/cardiovascular, gastrointestinal/liver, musculoskeletal/extremities, dermatology/skin, thyroid, lymph nodes, neurological/psychiatric conditions.

A complete physical examination will be performed at screening and the EOT visit; A brief physical examination will be performed at all other visits with the primary objective of assessing physical complaints and symptoms.

### 7.1.6. Electrocardiogram

A single 12-lead ECG should be collected at screening, within 1 h before infusion and within 1 h after the EOI on Cycle 1 Day 1, on Days 2, 3, 8, 15 in Cycle 1, within 1 h before infusion and within 1 h in each subsequent cycle, as clinically indicated, and at the EOT visit. ECG assessment, if scheduled at the same timepoint as the pharmacokinetic (PK) blood draw, should be performed before the collection of the blood sample. All ECGs should be performed after the subject has rested in a quiet supine position for at least 10 min. The examination contents include at least heart rate, QT, QTc (Fridericia's formula) and P-R interval. The test (each at least 10 minutes apart) will be repeated three times at screening. The average of the three QTcBs will be used as the baseline QTcB.

## 7.2. Pharmacokinetic (PK), Pharmacodynamic (PD), Cytokine, Immune Cell Subtype, Immunogenicity and Biomarker Studies

### 7.2.1. PK Sample Collection

Blood samples will be collected from all subjects at specified timepoints (see Section 3.8 for details) and serum concentrations of JS004 and toripalimab will be determined using a validated immunoassay method for PK analysis. Serum concentration measurements will be collected at the same time as ADA sample collection. Additional blood samples will be collected from subjects who experience unexpected and/or serious AEs.

The actual collection time of each blood sample will be recorded. See the Laboratory Manual for specific requirements for centrifugation, storage, and shipment of samples.

### 7.2.2. PD Sample Collection

In the dose escalation and dose expansion phases of Part A and Part B, blood samples will be collected within 1 h before the first dose of JS004, 24 h (±1 h) and 168 h (Day 8, ±8 h) after the start of the first dose, within 1 h before administration in Cycles 2, 4, 6 and 8, at the end of treatment (±7 days), and at the safety follow-up visit for assessment of BTLA receptor occupancies (RO) on CD3+, CD4+CD45RA+, CD8+CD45RA+, CD3-CD20+, CD3-CD56+ cells. See the Laboratory Manual for specific operation requirements in detail.

### 7.2.3. Sample Collection for Cytokine Study

In the dose escalation and dose expansion phases of Part A and Part B, blood samples will be collected within 1 h before the first dose of JS004 and 24 h (±1 h) after the start of the first dose for evaluation of the effect of JS004 on cytokines (IFN-γ, IL-8, IL-4, IL-6, IL-10, TNF-α). See the Laboratory Manual for specific operation requirements in detail.

### 7.2.4. Sample Collection for Immune Cell Subtype Study

In the dose escalation and dose expansion phases of Part A and Part B, blood samples will be collected within 1 h before the first dose of JS004, 24 h (±1 h) and 168 h (Day 8, ±8 h) after the start of the first dose, within 1 h before administration in Cycles 2, 4, 6 and 8, at the end of treatment (±7 days), and at the safety follow-up visit for evaluation of the effect of JS004 on peripheral blood immune cell subtypes (CD4+, CD3+CD4+/CD3+CD8+, CD20+, CD3-CD20-NKG2A+ cells, etc.). See the Laboratory Manual for specific operation requirements in detail.

### 7.2.5. Immunogenicity Sample Collection

Blood samples will be collected within 1 h before infusion of JS004, at the end of treatment (±7 days), and at the safety follow-up visit in Cycles 1, 2, 4, 6, 8, 12, 16 and every 8 cycles thereafter for immunogenicity analysis in Part A and Part B. The blood collection volume will be 5 mL. It mainly includes the incidence and titer analyses of JS004 anti-drug antibodies (ADA) and toripalimab anti-drug antibodies (if applicable) and the incidence analysis of serum neutralizing antibodies (Nab). In addition, if an IRR event occurs, ADA samples will be collected at the start of infusion reactions (+5 min), at the resolution of the reaction (+5 min), and at 30 days (±3 days) after the reaction. If ADA is positive, neutralizing activity will be assessed.

### 7.2.6. Biomarker Sample Collection (Optional)

Tumor tissue samples will be collected within 28 days before the first dose of JS004 (screening period). Fresh biopsy tissues will be collected as far as possible, and archived tumor tissues are acceptable for those from whom fresh tissues cannot be collected. Formalin-fixed, paraffin-embedded tumor tissue sections will be analyzed for detection of BTLA, HVEM, PD-L1 expression in tumor tissue and CD8 positive tumor infiltrating immune cell density and whole-exome sequencing (WES) using validated immunohistochemical methods;

Blood samples (approximately 6 mL) will be collected into vacuum anticoagulated tubes for biomarker study before the first dose in Cycle 1, at each imaging assessment, and at the end of treatment visit. Two milliliters of blood will be collected before the first dose of JS004 in Cycle 1 for WES detection of tumor tissue paired blood. See the Laboratory Manual for specific operation requirements in detail.

## 7.3. Efficacy Assessment

Efficacy assessments include all known or suspected sites of disease. Computed tomography (CT) or magnetic resonance imaging (MRI) scans of the brain, chest, abdomen, pelvis, and any other known sites of disease (eg, neck) and FDG-PET are required at baseline. Bone scan and/or bone X-ray can be used for subjects with known or suspected bone metastases. Head imaging examination will be used for subjects with known or suspected brain metastases during the trial.

Tumor imaging assessments will be performed every 9 weeks (±7 days) after the first dose of the investigational product until any of the following occurs: initiation of new anti-tumor therapy, progressive disease, withdrawal of consent, study discontinuation, loss to follow-up or death. Efficacy assessments will be performed per Lugano 2014 Criteria (Annex 1).

For subjects with lymphoma, complete response (CR) assessed by CT or MRI should be confirmed by FDG-PET within 4 weeks. For subjects with confirmed bone marrow involvement at baseline, a repeat bone marrow aspiration biopsy (only FDG-PET confirmation is needed for Hodgkin lymphoma) is required to confirm radiographic CR during the trial within 2 weeks of completion of imaging. For subjects who achieve PR or CR for the first time, the presence of B symptoms should be evaluated; If a PR is obtained prior to CR, then B symptoms are to be assessed again at the time of CR.

Subjects who experience the progression as defined by Lugano 2014 Criteria may continue treatment but should undergo repeat imaging within 4-8 weeks (± 7 days) for confirmation of progression if they meet the following conditions:

a. No clinically significant symptoms or signs of progressive disease;

b. No decrease in Eastern Cooperative Oncology Group (ECOG) performance status;

c. No symptomatic rapid progression that requires urgent medical intervention (such as symptomatic pleural effusion or spinal cord compression, etc.).

For subjects who meet the above criteria, the informed consent must be obtained again before continuing the treatment at the time of initial radiographic progressive disease. The informed consent form should fully inform all available standard therapies and the risks and benefits of continuing the treatment with the study drug. Sufficient communication and discussion should be made between the investigators and the sponsor, as well as between the investigators and the subjects. The study can be conducted only after they consider that the benefits of continued treatment outweigh the risks.

# 8. Adverse Events

The principal investigators should ensure that all personnel involved in the study, including the clinical coordinator, are aware of this section. Investigators are responsible for monitoring, recording, and entering events that meet the definition of an adverse event (AE) or serious adverse event (SAE).

## 8.1. Definitions

### 8.1.1. Description of Adverse Events

Adverse events refer to any adverse medical event in a clinical trial subject administered with a medicinal product, which does not necessarily have a causal relationship with the treatment. Adverse events can be any adverse undesirable symptoms, signs, laboratory abnormalities or diseases, including the following:

1) Aggravation of pre-existing (before enrollment) medical conditions (including aggravation of symptoms, signs, laboratory abnormalities), excluding events definitely related to tumor progressive disease;

2) Any new adverse events: Any new adverse medical conditions (including symptoms, signs, newly diagnosed diseases);

3) Clinically significant abnormal laboratory values or results not caused by concomitant diseases.

### 8.1.2. Definition of Serious Adverse Events

A serious adverse event (SAE) refers to an AE that meets one or more of the following criteria:

- Fetal;
- Life-threatening (defined as an event in which the subject is at immediate risk of death at the time of the event, not an event that hypothetically might have caused death if it were severe in the future);
- Requiring hospitalization or prolongation of existing hospitalization;
- Leading to permanent or significant disability/incapacity;

- Congenital anomaly or birth defect;
- **Other significant medical events** (defined as events that are not immediately life-threatening, or result in death or hospitalization. However, based upon appropriate medical and scientific judgment, they may jeopardize the subject or require medical or surgical intervention to prevent one of the serious outcomes listed in the definition above). Examples of such events include but are not limited to the following: allergic bronchospasm requiring intensive treatment in the emergency room or at home, hematologic cachexia or convulsions that do not result in hospitalization, potential drug-induced liver injury, the suspected transmission of pathogens (e.g., pathogenic or nonpathogenic pathogens) through the study drug, secondary tumors and so on.

SAE reporting waiver rationale:

- Death caused by progressive disease is an indicator of efficacy endpoint and does not need to be reported as an SAE;
- The following specific hospitalizations and prolonged existing hospitalizations are not required to be reported as SAEs:
    - Hospitalization for a pre-existing condition or scheduled visit, and hospitalization specified in the study protocol during the study without new adverse events or worsening of pre-existing conditions (e.g., procedures, administration and efficacy evaluation as required by the study protocol are not considered as hospitalization; Routine clinical treatment for CTCAE Grade 1-2 laboratory abnormalities at the time of hospitalization for scheduled visits is not considered to result in prolongation of existing hospitalization, which may be recorded as a non-serious AE according to the definition of AE);
    - Being hospitalized before entering the study and no change in medical condition during hospitalization. Also including scheduled elective surgeries/treatments before entering the study;
    - Hospitalization for administrative reasons (e.g., routine yearly physical examination);
    - Hospitalization due to medical insurance reimbursement and other reasons (such as common cold, conditioning, and other conditions that routinely do not require hospitalization).

## 8.2. Recording and Reporting of Adverse Events

Investigators are responsible for ensuring that all AEs are recorded on the AE page of the EDC and reported to the sponsor in accordance with instructions in the protocol.

Each AE recorded on the EDC will be evaluated for seriousness, severity, and causality by the investigators.

### 8.2.1. Time Period for Reporting of Adverse Events

Investigators will actively collect information on AEs at each study visit or patient contact. All AEs (whether spontaneously reported by the patient or evaluated and found by the investigator) should be recorded on the AE page of the EDC.

All SAEs should be reported from the time the patient signs the informed consent form until before the first dose.

After initiation of the study drug, all treatment-emergent AEs should be recorded and all SAEs/pregnancies should be reported until the end of the safety follow-up period (90 days after the last dose of the study drug or initiation of a new anti-tumor therapy, whichever occurs first).

After the safety follow-up period, any SAE assessed as related to the previous investigational product at the discretion of the investigator should be reported to the sponsor.

### 8.2.2. Follow-up of Adverse Events

1) After the initial report of an AE/SAE, the investigator is required to proactively follow up each subject at subsequent visits/contacts.
2) Whenever medically indicated (possibly beyond the safety follow-up period after the last dose of the study drug), an AE that is not resolved at the time of the last AE evaluation or other assessments during the study should be followed up by the investigator, but not recorded in the EDC. The sponsor will reserve the right to request additional information on any subject with any ongoing AE/SAE at the end of the study, if deemed necessary.
3) The investigator should follow up all AEs until any of the following occurs:
    - AE is relieved or improved to baseline level or better;
    - The event is stable and no further improvement is expected, as confirmed by the investigator;
    - Death of the subject;
    - The subject has lost contact or withdrawn the informed consent;
    - The investigator confirms that the AE is not related to the study treatment;
    - The subject starts a new anti-tumor therapy.

### 8.2.3. Serious adverse event report

The investigator should complete the standard SAE reporting form provided by the sponsor and report SAEs and protocol-specified adverse events of special interest (AESIs, if applicable) to the sponsor within 24 h after being informed. If the investigator fails to obtain all information on SAEs at the time of the initial report (e.g., the subject visits another hospital), the investigator should be responsible for continuing to follow up and promptly reporting the additional information within 24 h after being informed. For fatal or life-threatening SAEs where important or relevant information is missing, active follow-up should be undertaken immediately.

If the investigator believes the information previously reported for an SAE is misreported, corrections, withdrawals, or downgrades may be made in the follow-up report and reported according to the SAE reporting procedure.

In addition, the investigator should promptly clarify SAE medical queries raised by the sponsor or provide specific supplementary information.

### 8.2.4. Laboratory Abnormality

Blood samples/urine samples/other samples for laboratory tests will be collected according to the study flowchart for testing at the central laboratory.

Hepatic function abnormal defined by Hy's Law should be reported as an SAE: liver biochemistry should be further evaluated if elevated. Obstructive jaundice due to progressive disease or hyperbilirubinemia for other reasons will be excluded if AST or ALT ≥3×ULN and total bilirubin ≥2×ULN. If the event does not result in hospitalization of the subject, etc., "significant medical events" can be selected for SAE criteria.

### 8.2.5. Progressive Disease

The progressive disease may increase the severity of the disease under study and/or worsen disease-related symptoms. The appearance of new lesions or increases in the size of pre-existing lesions in the primary tumor under study should be considered a progressive disease rather than an AE. Events that are unequivocally due to progressive disease should not be reported as an AE during the study.

### 8.2.6. Death

All deaths that occur during the study treatment period or during the safety follow-up period after the last dose of study treatment as defined in this protocol must be reported as follows:

- It is clear that death caused by disease progression is defined as an indicator of efficacy endpoints and does not need to be reported as SAE, but should be recorded on the death record page of the eCRF. Such deaths will not be reported as SAEs.
- When the death is not due to (or is undetermined) the progression of the disease under study, the AE leading to death must be reported as an SAE. It should also be recorded on the death record page in the eCRF. The report should describe possible PD and complications, if applicable, and indicate the primary and secondary causes of death.
- Unexplained deaths should always be reported as SAEs. It should also be recorded on the death record page in the eCRF. Necropsy may contribute to assessing the cause of death, and a copy of the autopsy findings should be sent to the sponsor if an autopsy is performed.

Deaths that occur after the protocol-specified safety follow-up period after the last dose of study treatment should be recorded on the death record page. If a death is due to an event that occurs after the protocol-specified safety follow-up period, and the event is considered to be due to delayed toxicity of the study drug, the death should also be reported as an SAE.

#### 8.2.7. Pregnancy Report

All pregnancies must be reported after the start of the study drug until the end of the safety follow-up period. Female subjects who become pregnant during the clinical trial will be withdrawn from the study. The investigator should report it to the sponsor within 24 hours of awareness and complete the Pregnancy Report/Follow-up Form for Junshi clinical trials.

If the spouse of a male subject becomes pregnant during the study, the subject will continue to participate in the study. The investigator should report this event to the sponsor within 24 h after being informed and complete the Pregnancy Report/Follow-up Form for Junshi Clinical Trials.

The investigator should follow up on the pregnancy outcome until 1 month after delivery and report the outcome to the sponsor.

Abnormal pregnancy outcomes (e.g., ectopic pregnancy, stillbirth, spontaneous abortion, fetal malformation, fetal death) are considered SAEs and should be reported within the specified time limit for SAE reporting.

If a female subject experiences a concurrent SAE during the pregnancy, the appropriate SAE Report Form also needs to be completed and reported according to the SAE reporting procedures.

#### 8.2.8. Drug Overdose Reporting

Drug overdose refers to the accidental or deliberate use of an excessive and medically significant dose. A dose exceeding the protocol-prescribed dose of any study drug can be considered an overdose. Simple drug overdose events do not need to be reported as SAEs, but if an AE/SAE occurs due to drug overdose, the AE/SAE should be recorded respectively. Otherwise, the drug overdose event should only be recorded as a protocol violation.

### 8.3. Criteria for Severity Grading of Adverse Events

Refer to the NCI-CTCAE v5.0 AE grading criteria.

**In case of AEs not listed in NCI-CTC AE V5.0, please refer to the following criteria in the table below:**

**Table 25 NCI-CTCAE V5.0 Common Criteria for Severity of AEs**

| Grade | Clinical description of the severity |
|---|---|
| 1 | Mild; asymptomatic or mild symptoms; only clinical or laboratory abnormalities; treatment not indicated |
| 2 | Moderate; minimal, local, or noninvasive intervention indicated; limiting age-appropriate instrumental activities of daily living*. |
| 3 | Severe or medically significant but not life-threatening; hospitalization or prolongation of existing hospitalization indicated; disabling; limiting activities of daily living**. |
| 4 | Life-threatening consequences, urgent intervention indicated |
| 5 | Fatal due to adverse event |
| Note: *Daily life using tools: refers to cooking, shopping, calling, financial management, etc.<br>**Self-care in daily life refers to bathing, dressing, undressing, eating, using the toilet, taking medicine, and not bedridden | |

## 8.4. Criteria for Relationship of Adverse Events to the Study Drug

The investigator should determine whether the adverse event is reasonably related to the study drug, based on his/her knowledge of the patient, the patient's condition before and after the adverse event, and evaluation of any potential causes. The assessment should be based on the following principles:

- Temporal relationship between the occurrence of the event and the start of the study drug treatment
- Event course, especially response to dose reduction, discontinuation or re-introduction (if applicable)
- Whether the event is consistent with the known pharmacological/toxicological profile of the study drug
- Whether the event is related to the disease under study
- Whether the patient has risk factors or concomitant medications known to cause an increase in the incidence of the event
- Non-therapeutic related factors known to be associated with the occurrence of the event.

The investigator will determine the correlation between AEs and the study drug based on his/her clinical judgment and the following definitions into five levels: related, probably related, possibly related, unlikely related, and unrelated. The first four items are "yes" for the correlation with the drug. The specific judgment criteria are as follows:

| | |
|---|---|
| Definitely related | Occurrence after administration of the study drug or with a reasonable temporal relationship between the onset of AE and administration of the study drug, and the AE is more plausibly explained by the study drug than by other causes. Positive dechallenge and rechallenge (if applicable) results. |
| Probably related | Occurrence after administration of the study drug or with a reasonable temporal relationship between the onset of AE and administration of the study drug, and the AE is more plausibly explained by the study drug than by other causes. Positive dechallenge results. |
| Possibly related | Occurrence after study drug administration or with a reasonable temporal relationship between the onset of AE and administration of the study drug, but there are some other explanations for the etiology of AE. Positive dechallenge results. |
| Unlikely related | Occurrence after administration of the study drug or with a temporal relationship between the onset of AE and administration of the study drug(e.g., a delayed side effect), but there are other plausible explanations for the etiology of AE. Negative or ambiguous dechallenge results. |
| Definitely unrelated | The study drug is not used, or there is no reasonable relationship between the use of the study drug and the time of occurrence of AE, or the adverse event has another definite cause. |

## 8.5. Toxicity Management for Specific Adverse Events

### 8.5.1. Infusion Reactions

After the first infusion of an antibody drug, such as JS004, subjects may experience fever, headache, nausea, vomiting, or hypotension. These AEs usually result from target cell lysis, cytokine release, or complement activation. The investigator will manage AEs related to infusion reactions according to local standard procedures or recommendations listed in Table 26.

**Table 26 Management of Infusion-Related Reactions (NCI CTCAE Grading)**

| | |
|---|---|
| Grade 1 | Decrease the infusion rate by 50%, and monitor closely for any signs of worsening. Medical management actions are taken as needed. |
| Grade 2 | Temporarily stop the infusion; Strat intravenous infusion of normal saline; IV administer diphenhydramine 50 mg (or equivalent) and/or acetaminophen 325 to 1000 mg (paracetamol); Consider administration of corticosteroids and bronchodilators; Bedside monitor the subject until recovery from symptoms.<br>Once infusion-related reactions are resolved within 1 hour of drug infusion interruption or resolved to at least Grade 1 in severity, The infusion will be restarted at 50% of the initial infusion rate. If no further complications occur after the 30-minute infusion, the infusion rate can be increased to 100% of the initial infusion rate; Monitor subjects closely.<br>Symptom recurrence: discontinue further treatment at this visit; Administer diphenhydramine 50 mg IV, stay at the bedside, and monitor the subject until recovery from symptoms. The amount of the infused study drug must be recorded in the source documents and the eCRF. |
| Grade 3 to 4 requires treatment discontinuation. | Stop the infusion; monitor the subject until recovery from symptoms. If needed, bronchodilators, epinephrine 0.2 to 1 mg as a 1:1,000 solution for subcutaneous injection or 0.1 to 0.25 mg as a slow 1:1,000 solution for intravenous injection, and/or diphenhydramine 50 mg IV and methylprednisolone 100 mg (or equivalent), are recommended.<br>Subjects should be monitored until symptoms will not recur in the opinion of the investigator. Investigators should follow their institutional guidelines for the treatment of anaphylactic reactions. Symptomatic treatment (e.g., oral antihistamines or corticosteroids) may be given for symptoms (e.g., local or generalized pruritus within 1 week of treatment) of delayed-type hypersensitivity. Subjects will no longer be treated with JS004. |

### 8.5.2. Hypersensitivity

Type I hypersensitivity reactions or allergic reactions (e.g., shortness of breath, urticaria, anaphylactic reactions, angioedema) could theoretically be adverse reactions of all protein injections. Immune complex-mediated type III hypersensitivity reactions are similar in AEs to type I reactions but may be delayed post-infusion reactions, and symptoms may include, for example, rash, urticaria, polyarthritis, myalgia, synovitis multiplex, fever, and, if severe, glomerulonephritis.

All subjects should be closely observed during the infusion of the investigational product, and thereafter should be monitored for clinical symptoms of systemic reactions to prevent clinical signs of allergic reactions/hypersensitivity reactions. The investigator will manage anaphylaxis/hypersensitivity reactions according to local standard procedures or following the recommendations in Table 27.

**Table 27 Management of Allergic Reactions/Hypersensitivity (NCI CTCAE Grading)**

| | |
|---|---|
| Grade 1 (allergic reaction or cytokine release syndrome) | Monitor the subject for worsening of the condition. If the reaction worsens, discontinue the infusion. Prophylactic medication should be given for subsequent infusions. |
| Grade 2 (allergic reaction or cytokine release syndrome) | Suspend the infusion of JS004.<br>If medically indicated, give bronchodilators, oxygen, acetaminophen, etc. Once the severity of the reaction is resolved to ≤Grade 1, the infusion will be resumed at 50% of the previous infusion rate. Monitor subjects closely for worsening symptoms.<br>If the reaction recurs, stop the infusion. Prophylactic medication should be given for subsequent infusions as described above. |
| Grade 3 to 4 (Allergic reaction or cytokine release syndrome or anaphylaxis) | Stop the infusion of JS004 immediately and disconnect the subject's intravenous line.<br>If medically indicated, give epinephrine, bronchodilators, antihistamines, glucocorticoids, intravenous fluids, vasopressors, oxygen, etc.<br>Call the sponsor or designated representative to report serious adverse events (SAEs); Study treatment should be discontinued for NCI-CTCAE Grade 3 or 4 hypersensitivity reactions. |

PK, pharmacodynamics, and ADA sampling should continue as long as the sampling does not interfere with the subject's study drug treatment. If cytokine release syndrome is suspected, a serum sample should be provided for cytokine release assay by a central laboratory as long as the sampling does not interfere with the subject's study drug treatment.

### 8.5.3. Extravasation

If extravasation occurs, the infusion should be stopped immediately and the investigator should be consulted immediately. Handling of extravasation should follow the local standard of care.

### 8.5.4. Immune-related adverse events (irAE)

Investigators should carefully document, monitor, and evaluate irAEs. If deemed necessary by the treating investigator, the patient should receive appropriate supportive care measures and treatment.

An irAE may occur after the first dose or months after treatment discontinuation and may involve multiple organ systems. Suspected irAEs should be adequately evaluated to determine etiology or to rule out alternative contributors; Additional procedures or tests may be conducted in this assessment. Dose modification and toxicity management guidelines for irAEs related to immune checkpoint inhibitor therapy are described above and recently published by the American Society of Clinical Oncology (ASCO) in December 2018 and are provided in Annex 4. Given the observations in the BTLA-deficient mouse study showing exacerbation of asthma, an autoimmune disease involving central nervous system autoimmunity, and systemic lupus erythematosus[3], it is considered that the irAEs of JS004 may include these diseases.

Therefore, regardless of the organ involved, the following are general recommendations to be followed for JS004 treatment. See Annex 3 for specific organ management. (Note: Grade definitions are provided in each table and follow the CTCAE [version 5.0] in most cases).

In general, investigators are advised to manage irAE toxicities as follows:

- For Grade 1 toxicities, except for some neurological, hematological, and cardiac toxicity, JS004 infusion should be continued and closely monitored.
- For most Grade 2 toxicities, JS004 infusion should be interrupted and resumed when symptoms and/or laboratory values recover to ≤Grade 1. Corticosteroids (starting dose 0.5 to 1 mg/kg/day prednisone or equivalent) may be administered according to ASCO or institutional guidelines. Corticosteroids should be withdrawn gradually over a period of at least 4 to 6 weeks.
- Hold JS004 infusion for Grade 3 toxicities and initiate high-dose corticosteroids (prednisone 1 to 2 mg/kg/d or methylprednisolone IV 1 to 2 mg/kg/d). Corticosteroids should be withdrawn gradually over a period of at least 4 to 6 weeks. If symptoms do not resolve within 48 to 72 hours after high-dose corticosteroid therapy, infliximab may be administered per ASCO or institutional guidelines to treat toxicities.
- Restart JS004 infusion when symptoms and/or laboratory values return to Grade ≤1; However, caution is recommended, especially in patients with early-onset irAEs.

## 9. Data Analysis/Statistical Method

The detailed methods for summarization and statistical analysis of the data collected for this study will be included in the Statistical Analysis Plan (SAP) and finalized and filed by the sponsor. If any change to this study protocol will significantly impact the SAP at the discretion of the sponsor or the principal investigator, the SAP should be revised to keep consistency with the protocol. The statistical analysis plan must be finalized prior to database lock.

### 9.1. Analysis Set

(1) Full analysis set: It includes all subjects who receive at least one dose of the study drug. This analysis population is the primary analysis population for this study.

(2) Evaluable analysis set: It includes all enrolled subjects who receive at least one dose of the investigational product and have baseline data and at least one tumor evaluation.

(3) DLT analysis set: It includes all subjects who receive at least one dose of the investigational product, complete the first treatment course or withdraw from the trial prematurely due to adverse events during the first treatment course. This analysis set will be used to analyze and summarize DLT events.

(4) PK analysis set: The analysis set of this study includes all subjects enrolled who receive at least one dose of the investigational product, and have post-dose PK evaluation data.

(5) Immunogenicity analysis set: It includes all subjects enrolled who receive at least one dose of the investigational product, and have baseline and at least one post-baseline immunogenicity evaluation data.

(6) PD analysis set: It includes all subjects enrolled who receive at least one dose of the investigational product, and have baseline and at least one post-baseline PD evaluation data.

(7) Cytokine analysis set: It includes all subjects enrolled who receive at least one dose of the investigational product, and have baseline and at least one post-baseline cytokine evaluation data.

(8) Immune cell subtype analysis set: It includes all subjects enrolled who receive at least one dose of the investigational product, and have baseline and at least one post-baseline immune cell subtype evaluation data.

(9) BTLA, HVEM, PD-L1 and CD8 expression analysis set: It includes all subjects enrolled who receive at least one dose of the investigational product and have evaluation data of protein expression of any one of the four proteins of BTLA, HVEM, PD-L1 and CD8.

(10) Gene mutation analysis set: It includes all subjects enrolled who receive at least one dose of the investigational product and have whole exome sequencing data.

(11) Peripheral blood immune cell surface receptor analysis set: It includes all subjects enrolled who receive at least one dose of the investigational product, and have baseline and at least one post-baseline peripheral blood immune cell surface receptor evaluation data.

## 9.2. Efficacy Evaluation

The investigator will perform tumor assessment per Lugano 2014 Criteria. The evaluation data will be based on the following response measures: complete response (CR), partial response (PR), stable disease (SD), progressive disease (PD), and not evaluable (NE).

Efficacy measures in this study include ORR, BoR, DoR, DCR and PFS. Objective response rate (ORR): 95% confidence interval of the estimated ORR will be calculated using the Clopper-Pearson method.

Best overall response (BoR) will be assessed preliminarily.

Disease control rate (DCR): 95% confidence interval of its estimate will be estimated using the Clopper-Pearson method.

Duration of response (DoR): The median DoR will be estimated based on the Kaplan-Meier (KM) method and its 2-sided 95% confidence interval will be calculated.

Progression-free survival (PFS): The median PFS will be estimated based on the KM method and its 2-sided 95% confidence interval will be calculated. Survival curves will be plotted using the Kaplan-Meier method.

## 9.3. Sample size determination

A total of approximately 170 subjects are planned to be enrolled in this study.

**Part A:** Approximately 9-18 subjects are planned to be enrolled in the dose escalation study, and approximately 18 subjects are planned to be enrolled in the expansion study of the selected dose group. Peripheral T-cell lymphoma, Hodgkin lymphoma, follicular lymphoma or other subtypes are preferentially selected in the indication expansion phase, which can be adjusted according to study progress. Approximately 15 subjects are enrolled for each indication (including the number of subjects in the dose expansion phase, which can be adjusted according to study progress). Approximately 80 subjects are expected to be enrolled in Part A.

**Part B:** There are 2 dose groups for dose escalation of the combination treatment, with 3-6 subjects in each dose group, and 6-12 subjects are expected to be enrolled. Then, the recommended dose will be determined by the sponsor and the investigator upon discussion, and the enrollment will be extended to 6-18 subjects (for example, the recommended dose is used in 2 dose groups, and 6-9 subjects will be enrolled in each group). The number of subjects will be adjusted subsequently according to study progress. Peripheral T-cell lymphoma, Hodgkin lymphoma, follicular lymphoma or other subtypes are preferentially selected in the indication expansion, which can be adjusted according to study progress. Approximately 20 subjects will be enrolled for each indication (including the number of subjects in the dose expansion phase. The specific number of subjects can be adjusted according to study progress. Approximately 90 subjects are expected to be enrolled in this phase.

## 9.4. PK/PD/Cytokine/Immune Cell Subtype/Immunogenicity/Biomarker Study Analyses

### 9.4.1. PK Analysis

The PK population will include all subjects with at least one blood sample yielding evaluable PK data.

Plasma PK parameters of JS004 will be estimated using the non-compartmental model, including $AUC_{0-\infty}$, $AUC_{0-t}$, $AUC_{0-21d}$, $C_{max}$, $C_{min}$, $T_{max}$, $t_{1/2}$, CL, $V_{ss}$ and $R_{ac}$, etc. Descriptive statistics will be summarized for PK parameters of JS004. All statistical summaries will be presented by dose group.

### 9.4.2. PD Analysis

Statistical variables of BTLA receptor occupancy (e.g., mean and standard deviation, median and minimum/maximum) will be determined at screening and post-treatment. The percent change in the same parameter from baseline will also be calculated for each pair of specimens.

#### 9.4.3. Cytokine Analysis

Based on the cytokine analysis set, descriptive analysis will be performed for pre- and post-dose cytokine data.

#### 9.4.4. Immune Cell Subtype Analysis

Descriptive analysis will be performed for immune cell subtypes at different timepoints pre- and post-dose based on the immune cell subtype analysis set.

#### 9.4.5. Immunogenicity Analysis

Descriptive statistics will be summarized for the time to first occurrence and duration of positive anti-JS004 antibody and/or anti-toripalimab antibody, if applicable, based on the immunogenicity analysis set, and the positive rate of anti-JS004 antibody and/or anti-toripalimab antibody, if applicable, will be calculated.

#### 9.4.6. Biomarker Endpoint Analysis

Biomarker data may be analyzed using graphical methods and descriptive statistics, such as linear regression, t-test, and analysis of variance (ANOVA). The correlation of biomarker study results with PK parameters and anti-tumor efficacy measurements will be explored using statistical methods.

### 9.5. Safety Analysis

All subjects who receive one dose of JS004 at any dose will be included in the summaries and listings of safety data. The overall safety profile and tolerability will be characterized by type, frequency, severity, timing, duration, and relationship of adverse events and laboratory abnormalities to study treatment.

#### 9.5.1. Adverse Events

AEs will be coded using MedDRA (version 22 or higher). The severity of toxicities will be graded according to NCI CTCAE (version 5.0).

In all summaries, the focus will be on treatment-emergent adverse events (TEAEs), i.e., events that first occur or worsen in severity after the first dose of JS004. Adverse events will be summarized by the frequency of TEAE corresponding to MedDRA system organ class, preferred term, and the most severe grade of NCI CTCAE (version 5.0). A summary of TEAEs, i.e., events that are related or possibly related to JS004 at the discretion of the investigator, will also be provided.

AEs leading to discontinuation of JS004 treatment or withdrawal from the study, Grade 3 or higher AEs, serious adverse events, and death events will be listed.

All DLTs will be reported and the MTD will be identified.

### 9.5.2. Primary Endpoint Analysis

DLT is the primary endpoint in the dose escalation part of the study. As described in the study design section, the MTD is estimated based on the occurrence of DLTs observed in the dose cohorts. AEs constituting DLTs will be listed by dose level.

### 9.5.3. Laboratory Abnormality

The number and percentage of subjects experiencing laboratory abnormalities will be summarized according to the worst toxicity grade observed for each laboratory test, and the laboratory tests will be presented by whole study period and cycles (C1 and subsequent cycles). Cross tables will be provided to investigate the distribution of laboratory toxic effects.

For laboratory tests without a CTCAE grade definition, the results will be classified as normal, abnormal, or not tested.

### 9.5.4. ECG

Changes in QTc and any other ECG measurements from baseline will be summarized by standard timepoint using descriptive statistics.

# 10. Data Management Methods

## 10.1. Data Recording

### 10.1.1. Completion of Study Medical Records

As the source documents of the study, the original medical records should be kept complete. The authorized investigator is responsible for recording and keeping the original medical records at the study site. The true name and subject number on the cover of the medical records should be checked before each completion. The writing of the medical records should be neat and legible for data verification with the eCRF by the monitor of the sponsor each time.

### 10.1.2. eCRF Completion

Clinical study data will be collected using RAVE EDC.

Completion: The data in eCRF are derived from original medical records and laboratory test reports and should be consistent with the source documents. Any observation and examination results in the study should be timely, correctly, completely, clearly, normatively and truly completed in the eCRF, and no changes can be made at will. All items in the eCRF should be completed without empty or missing items.

Modification: If necessary, the reason for data modification should be filled in when data correction is made in the eCRF as prompted by the system.

Laboratory test: The investigators should perform each item according to the follow-up time window, and the subject information and data will be collected, entered and reported. Laboratory test report documents as one of the source documents should be complete, and test results should be timely entered into the eCRF.

In addition, other source data, including original prints of data recorded or generated by automated instruments, photographic negatives, X-rays, CT or MRI, ECG recordings, etc., must indicate, at a minimum, the subject number and the date the procedure is performed. If possible, the necessary documentation should be provided for the medical evaluation of these records, signed and dated by the investigator. Information on such source documents should be entered into the eCRF in a timely manner.

#### 10.1.3. eCRF Review

The investigator should complete, review and submit the eCRF for each subject within 3-5 working days after the end of treatment course. The investigator should promptly respond to the queries from the monitor, data manager and medical reviewer. After data cleaning, the investigator should sign the completed eCRFs for confirmation.

### 10.2. Data Monitoring

Performed by: Monitor.

Monitoring focus: protocol compliance ; correctness, completeness and consistency with medical records and laboratory test reports of eCRF filling; data errors or omissions. The monitor needs to check the consistency of the contents in the eCRF with the original documents.This process is known as Source Data Verification (SDV).

### 10.3. Data Management

#### 10.3.1. Establishment of EDC Database

The data manager establishes the study data collection system and database according to the study protocol, which will be ready for online use before the first subject is enrolled. Prior to use, all EDC users need to receive sufficient training and obtain the corresponding account to log in the system.

#### 10.3.2. Data Entry and Reconciliation

After the investigator completes and submits the eCRF, the monitor, data manager and medical personnel should review the data one by one, and ask the investigator to answer the questions in the review in the form of queries. After all is clear, the eCRF must be signed by the investigator.

#### 10.3.3. Data Review and Database Lock

After the clinical trial is completed, the study director, sponsor, statistician and data manager will jointly conduct a data review before statistical analysis. The important content is assigning each case to the analysis data set (including FAS, PPS and SS), the judgment of missing values and the handling of outliers. Any decisions made in the data review will be documented.

After reviewing that the established database is correct, the database will be locked. After the database is locked, the data should be properly stored for future reference, and the data should be submitted to statistical experts for statistical analysis.

#### 10.3.4. Data Archiving

Upon completion of the study, eCRFs in PDF format will be generated in the EDC system, stored on a non-rewritable memory (CD/DVD), and submitted to the sponsor and each site for future audits and/or inspection.

The storage and management of trial data must be carried out in accordance with GCP requirements. Investigators should keep clinical trial data for at least 5 years after discontinuing the clinical trial, and sponsors should keep clinical trial data for at least 5 years after the study drug is approved for marketing.

## 11. Termination and End of Study

### 11.1. Criteria for Study Termination by the Sponsor

The study may be terminated prematurely. This may be due to a regulatory authority decision, a change in IRB/EC opinion, an efficacy or safety issue with the study drug, or at the discretion of the sponsor. In addition, Junshi reserves the right to stop developing JS004 at any time.

If the study is prematurely terminated or interrupted, the sponsor will promptly notify the investigators and the regulatory authorities. The investigator must inform all participating subjects, the Ethics Committee and the relevant participating departments of the site within 7 days of the notification. Under the leadership of the sponsor, all study data should be collected and all eCRFs should be completed to the greatest extent possible.

### 11.2. End of Study

The end of study is defined as follows:

Last subject last visit (LSLV)

LSLV may be determined when dose escalation and study discontinuation conditions are met.

or:

Database lock.

## 12. Regulatory Ethics, Informed Consent and Subject Protection

### 12.1. Regulatory Considerations

The study is designed based on the following laws and regulations:

1) Provisions for Drug Registration
2) "Good Clinical Practice"

4) Consensus on international ethical guidelines, including the Declaration of Helsinki and international ethical guidelines of the Council for International Organizations of Medical Sciences (CIOMS)
5) ICH Guidelines
6) Other applicable laws and regulations

## 12.2. Ethical Norms

This study will be conducted in accordance with the Declaration of Helsinki, Good Clinical Practice (GCP) and relevant regulations promulgated by the National Medical Products Administration (NMPA), and Good Clinical Practice (GCP) developed by the International Conference on Harmonization (ICH).

This study will be carried out according to the protocol. Prior to initiation of the study, the protocol and its amendments and informed consent form should all be approved/agreed on by the Institutional Review Board/Independent Ethics Committee (IRB/IEC).

Without the consent of both the sponsor and the investigator, no party may unilaterally modify the protocol. The investigator may make changes or deviations to the study protocol only to eliminate an immediate or immediate hazard to the subject prior to obtaining approval from the Ethics Committee/Institutional Review Board. Meanwhile, the deviations or changes made and their causes, and the proposed amendments to the protocol should be submitted to the Ethics Committee/Institutional Review Committee for review and approval as soon as possible. The investigator must explain and record any protocol deviation made.

During the study, any modifications made to the protocol should be submitted and, if necessary, along with other accordingly revised study documents, to the Ethics Committee for approval. Investigators are responsible for periodic submission of the in-trial reports to the Ethics Committee as required and should inform the Ethics Committee that the trial has ended after the trial.

Any serious violations must be immediately reported to Junshi. Serious violations refer to violations of the GCP and principles applicable to the study or violations of the protocol, which may have an important impact on subjects' safety or physical health or the scientific value of the study.

The personnel involved in implementing the study must have appropriate qualifications, educational background and training experience, and special experience in their respective work.

## 12.3. Institutional Review Board/ Independent Ethics Committee

Before the initiation of the study, the investigator must first obtain a dated written approval/consent of the IRB/IEC for the protocol, informed consent form, subject recruitment materials (e.g., advertising) and all other written materials to be provided to the subject. The investigator or Junshi should also submit to the IRB/IEC an Investigator's Brochure or a product package insert to be provided to the subject and any update thereto.

The investigator or Junshi should submit reports, updates and other materials (e.g., expedited safety reports, revisions and administrative letters) to the IRB/IEC in accordance with the requirements of competent authorities or the procedures of the site.

## 12.4. Informed Consent

### 12.4.1. Informed Consent

The investigator or his/her designated representative will be responsible for explaining the study background, pharmacological characteristics of the study drug, the protocol, and benefits and risks of participating in the study to the subject or his/her legally acceptable representative. A written informed consent form signed jointly by the subject or his/her legally authorized representative and the study physician should be obtained before the subject enters the study (prior to screening).

The final ICF text should contain the following: study objectives, study procedures, subject's obligations, foreseeable benefits as well as risks and inconveniences for subjects participating in the study; treatment and appropriate insurance compensation to be provided to subjects if study-related injuries occur; access to study data and confidentiality of subjects' information, etc. Written approval of the ICF should be obtained from regulatory authorities in accordance with applicable laws and regulations, and the informed consent form should be written in a language readable to the subjects.

The ICF should be signed and dated by the patient or his/her legal representative, the investigator or his/her representative performing the informed consent process. The informed consent process should be documented in the medical history or clinical records for each patient, and a written informed consent should be obtained before participating in the study. Both the investigator and the subject should maintain one copy of the ICF. If important new data involving the study drug are identified, the ICF must be revised in writing and submitted to the Ethics Committee for review and approval. The patients still in follow-up or under treatment should go through the informed consent process again.

Revised safety information will be shared with investigators, institutional ethics committees, and patients to ensure that patients can take into account any pertinent and regularly updated information that might influence their decision to continue participating in the study..

### 12.4.2. Informed Consent Process and Records

Informed consent begins before an individual's consent to participate in a clinical study and continues throughout the entire course of the study. The risks and possible benefits of participating in the study will be discussed in detail and adequately with subjects and their families. Subjects will be required to read and review the informed consent form approved by the Ethics Committee. The investigator will explain the study to subjects and answer any questions that subjects may ask. Subjects will have the opportunity to carefully review the written informed consent form and ask questions before signing. Subjects will have sufficient time to discuss the study with their agent and consider whether to participate in this study. Subjects are only allowed to participate in the study after signing the informed consent form. During the study, subjects may withdraw their consent at any time. A copy of the informed consent form will be retained by the subject. Even if the patient refuses to participate in the study, their rights and interests will be fully protected, and the quality of their medical care will not be affected in any way.

## 12.5. Confidentiality of Subject Information

The confidentiality of subject information is strictly kept by the investigator, study participants, the sponsor and its agent. Confidentiality also covers biological samples, genetic testing, and the subject's clinical information. The study protocol, files, data, and all other information generated therefrom will be kept strictly confidential. No relevant study or data information shall be disclosed to any unauthorized third party without the prior written approval of the sponsor.

The sponsor or its authorized representatives, EC/IRBs, third-party quality control/auditing institutions, or relevant auditors from individuals or government departments can access all documents and records that the investigator must maintain, including but not limited to medical and treatment records. The study site should allow access to these records.

The subjects' contact information will be securely stored at each study site and will only be used internally in the course of the study. At the end of the study, all records will continue to be kept in a safe place according to the time limit set by the local IRB and regulations.

Subject study data collected for statistical analysis and scientific reporting will be uploaded and stored at HRTAU EDC system, in which subjects' contact information or identification information should not be included. In contrast, individual subjects and their study data will all have a unique identification number. Study data entry and management systems used by the study staff at each study site have password security functions.

After the consent of the subjects and the approval of the Ethics Committee of the study site are obtained, de-identified biological samples (this study only involves venous blood samples, tumor tissue paraffin blocks or slides sent to the central laboratory for biomarker analysis) will be stored at the central laboratory. Data will be shared with the sponsor and each study site for the same purpose. These samples can only be used for biomarker analysis. Privacy of the subjects will be protected throughout the study.

After obtaining the subject's consent and the approval of the local IRB, the de-identified imaging data used for independent imaging assessments will be stored in the independent imaging assessment committee unit, and the subject's privacy will be protected throughout the process.

During the course of the study, the subject has the right to withdraw his/her consent for future use of his/her own biological specimens or data. However, if the study has ended, the subject will not be able to withdraw his/her consent for preservation of his/her own biological specimens or data.

When the study ends, access to study data and/or samples of the central laboratory can be allowed.

## 13. Confidentiality and Publication of Study Results

The objectives, content and results of this clinical trial and all future information must be kept strictly confidential. The copyright of all materials and results shall belong to the sponsor.

At the end of the trial or when the study data is sufficient (as reasonably determined by the sponsor), the investigator may prepare the materials from the trial for public publication. Such materials should be submitted to the sponsor for review and comment before public publication. In order to ensure that the sponsor can express its opinions and make relevant recommendations, materials for public communication should be submitted to the sponsor for review at least 60 days prior to submission for public publication, public communication or review by the public publication committee.

The investigator must agree to include all reasonable comments made by the sponsor relating to the article that the investigator intends to publish publicly.

During the review of the article intended for public publication, the sponsor will have the right to postpone the publication of the article in order to enable the sponsor to take measures to protect its patent information. All materials related to the trial shall not be published without the prior written consent of the sponsor. Except for legal reasons, the sponsor or the principal investigator may not disclose the study results to a third party until a bilateral agreement has been reached on data analysis and interpretation.

## 14. Clinical trial progress

Date of first subject enrollment: Aug. 2020

Expected date of last subject enrollment: Jun. 2022

Expected date of end of study: Jun. 2023

## 15. References

[1] gco.iarc.fr/today.

[2] Chowdhury, P.S., Chamoto, K., and Honjo, T. (2018). Combination therapy strategies for improving PD- 1 blockade efficacy: a new era in cancer immunotherapy. J Intern Med 283, 110-120.

[3] Watanabe, N., Gavrieli, M., Sedy, J.R., Yang, J., Fallarino, F., Loftin, S.K., Hurchla, M.A., Zimmerman, N., Sim, J., Zang, X., et al. (2003). BTLA is a lymphocyte inhibitory receptor with similarities to CTLA-4 and PD-1. Nat Immunol 4, 670-679.

[4] Sedy, J.R., Gavrieli, M., Potter, K.G., Hurchla, M.A., Lindsley, R.C., Hildner, K., Scheu, S., Pfeffer, K., Ware, C.F., Murphy, T.L., et al. (2005). B and T lymphocyte attenuator regulates T cell activation through interaction with herpesvirus entry mediator. Nat Immunol 6, 90-98.

[5] Ren, S., Tian, Q., Amar, N., Yu, H., Rivard, C.J., Caldwell, C., Ng, T.L., Tu, M., Liu, Y., Gao, D., et al. (2018). The immune checkpoint, HVEM may contribute to immune escape in non-small cell lung cancer lacking PD-L1 expression. Lung Cancer 125, 115-120.

[6] Haymaker, C.L., Wu, R.C., Ritthipichai, K., Bernatchez, C., Forget, M.A., Chen, J.Q., Liu, H., Wang, E., Marincola, F., Hwu, P., et al. (2015). BTLA marks a less-differentiated tumor-infiltrating lymphocyte subset in melanoma with enhanced survival properties. Oncoimmunology 4, e1014246.

[7] Inoue, T., Sho, M., Yasuda, S., Nishiwada, S., Nakamura, S., Ueda, T., Nishigori, N., Kawasaki, K., Obara, S., Nakamoto, T., et al. (2015). HVEM expression contributes to tumor progression and prognosis in human colorectal cancer. Anticancer Res 35, 1361-1367.

[8] Costello, R.T., Mallet, F., Barbarat, B., Schiano De Colella, J.M., Sainty, D., Sweet, R.W., Truneh, A., and Olive, D. (2003). Stimulation of non-Hodgkin's lymphoma via HVEM: an alternate and safe way to increase Fas-induced apoptosis and improve tumor immunogenicity. Leukemia 17, 2500-2507.

[9] Michael Boice, Darin Salloum1, et al. Loss of the HVEM tumor suppressor in lymphoma and restoration by modified CAR-T cells. Cell. 2016 October 06; 167(2): 405-418.

[10] Derre, L., Rivals, J.P., Jandus, C., Pastor, S., Rimoldi, D., Romero, P., Michielin, O., Olive, D., and Speiser, D.E. (2010). BTLA mediates inhibition of human tumor-specific CD8+ T cells that can be partially reversed by vaccination. The Journal of clinical investigation 120, 157-167.

[11] Thommen, D.S., Schreiner, J., Muller, P., Herzig, P., Roller, A., Belousov, A., Umana, P., Pisa, P., Klein, C., Bacac, M., et al. (2015). Progression of Lung Cancer Is Associated with Increased Dysfunction of T Cells Defined by Coexpression of Multiple Inhibitory Receptors. Cancer Immunol Res 3, 1344-1355.

[12] Fourcade, J., Sun, Z., Pagliano, O., Guillaume, P., Luescher, I.F., Sander, C., Kirkwood, J.M., Olive, D., Kuchroo, V., and Zarour, H.M. (2012). CD8(+) T cells specific for tumor antigens can be rendered dysfunctional by the tumor microenvironment through upregulation of the inhibitory receptors BTLA and PD-1. Cancer Res 72, 887-896.

[13] Nathan R. Miselis, D.L., Clifford Restaino, Toya Baral, Jane Xia, Roanna Ueda, Anandi Sawant, Jeanne Baker, Gopalan Raghunathan, Xinzhong Wang, Edward Bowman, Selvakumar Sukumar (2017). Antagonism of the co-inhibitory receptor BTLA enhances efficacy of anti-PD-1 treatment in murine syngeneic tumor models. Cancer Res 77(13 Suppl).

[14] Croft M. The evolving crosstalk between co-stimulatory and co-inhibitory receptors: HVEM-BTLA. Trends Immunol. 2005;26(6):292-4. pmid:15922943.

[15] Han, P.; Goularte, O.D.; Rufner, K.;Wilkinson, B.; Kaye, J. An inhibitory Ig superfamily protein expressed by lymphocytes and APCs is also an early marker of thymocyte positive selection. J. Immunol. 2004, 172, 5931-5939.

[16] Paulos CM, June CH. Putting the brakes on BTLA in T cell-mediated cancer immunotherapy J Clin Invest. 2010;120(1):76-80.

[17] Otsuki N, Kamimura Y, Hashiguchi M, Azuma M. Expression and function of the B and T lymphocyte attenuator (BTLA/CD272) on human T cells. Biochem Biophys Res Commun 2006 June 16;344(4):1121-7.

[18] Leach MW, Halpern WG, Johnson CW et al. Use of tissue cross-reactivity studies in the development of antibody-based biopharmaceuticals: history, experience, methodology, and future directions. Toxicol Pathol 2010 December;38(7):1138-66.

## Annex 1 Lugano 2014 Criteria

| Resolution | Metabolic Response (PET/CT) | Radiographic Response (CT) |
|---|---|---|
| CR | CMR (Complete Metabolic Response) | CRR (Complete Radiographic Response) |
| Lymph nodes and extranodal lesions | 5PS score: 1, 2 or 3 points, with/without residual lesions.<br>Note: For Waldeyer's ring, extranodal hypermetabolic uptake organs such as spleen or bone marrow stimulated with myeloid colony-stimulating factor, the uptake value may be higher than normal mediastinal/hepatic blood pools. At this time, the evaluation of CR should be compared with the background level | Longest diameter (Ldi) of lymph nodes ≤1.5 cm without extranodal lesions |
| Non-measurable | Not applicable | Absent |
| Organ enlargement | Not applicable | Return to normal |
| New Lesion | None | None |
| bone marrow | No FDA hypermetabolism | Morphology is normal; if indeterminate, the result of immunohistochemistry should be negative |
| PR | PMR (Partial Metabolic Response) | PRR (Partial Radiographic Response) |
| Lymph nodes and extranodal lesions | 5PS score: 4 or 5 points, with reduced uptake compared to baseline, the residual lesions can be of any size<br>Interim assessment: The above findings suggest disease response<br>Terminal assessment: The above findings suggest residual disease | At most 6 target lesions have SPD reduction ≥50%; if the lesion is too small to measure, the default value is 5 mm×5 mm; if the lesion is not visible, it is 0×0 mm; if the lesion is >5 mm×5 mm, but smaller than normal, the actual measured value is recorded; |
| Non-measurable | Not applicable | None/normal, resolved, no increase |
| Organ enlargement | Not applicable | The long diameter of the spleen is reduced >50% of the original long diameter increase; It is often assumed that the normal size of the spleen is 13cm. If it is 15cm, the long diameter of the spleen should be <14 for PR |
| New Lesion | None | None |
| bone marrow | Uptake values for residual lesions are higher than values in normal bone marrow but below baseline values (reactive diffuse uptake is allowed while receiving chemotherapy).<br>Consider MRI/biopsy/interim assessment for further diagnosis if lymph node lesions resolved but nodular local abnormal changes persist in the bone marrow | Not applicable |

| SD | No Metabolic Response | Stable disease |
|---|---|---|
| Lymph nodes and extranodal lesions | No metabolic response: intermediate/terminal phase rating 5PS score 4 or 5, no significant change in metabolism from baseline | <50% increase in SPD of up to 6 target lesions;<br>PD not achieved |
| Non-measurable | Not applicable | PD not reached |
| Organ enlargement | Not applicable | PD not reached |
| New Lesion | None | None |
| bone marrow | No significant change from baseline | Not applicable |
| **PD** | **Metabolic Progression** | **Radiographic Progression** |
| Individual lymph nodes and extranodal lesions | 5PS score of 4 or 5 points with increased uptake from baseline and/or new uptake increase in interim or terminal response assessment | Progression of at least one target lesion can be diagnosed, at the same time, lymph nodes/extranodal lesions should meet the following: LDi >1.5 cm,<br>PPD increase ≥50% from minimum, LDi/SDi increase 0.5 cm (if lesions ≤2 cm)/1.0 cm (if lesions >2 cm) from minimum;<br>If there is splenomegaly, the increase in the long diameter of the spleen is >50% of the increase in the original long diameter. The normal size of the spleen is often assumed to be 13cm. If the original size is 15cm, the long diameter of PD needs to be >16; If there is no splenomegaly at baseline, the long diameter must increase by at least 2 cm from the baseline; New or recurrent splenomegaly |
| Non-measurable | None | New or clear progression of existing non-measurable lesions |
| New Lesion | Occurrence of new hypermetabolic lesions associated with lymphoma (excluding infection, inflammation and other causes), biopsy or interim evaluation should be considered if the nature is unclear | Previously relieved lesions are re-enlarged; New lymph nodes >1.5 cm in any diameter; New extranodal lesions >1.0 cm in any diameters. If it is less than 1.0cm, it must be clear whether it is related to lymphoma; Confirm lymphoma-associated lesion of any size |
| bone marrow | New or recurrent FDG-avid lesion | New or recurrent involvement |

CR=complete response; PR = partial response; SD = stable disease; PD = progressive disease;
SPD: sum of the products of the largest perpendicular diameters; PPD: product of the longest diameter and its perpendicular diameter; LDi: longest transverse diameter; SDi: Shortest axis perpendicular to the LDi

### 5PS Score

| Scored | PET/CT Findings |
|---|---|
| 1 | Uptake value ≤ background |
| 2 | Uptake ≤ mediastinal blood pool |
| 3 | Mediastinal blood pool < uptake ≤ hepatic blood pool |
| 4 | Uptake > liver (mild) |
| 5 | Uptake > liver (significant, SUV max >2 times hepatic blood pool) or new lesions |

Measurable lesions:

Up to 6 significant lymph nodes/nodal confluent masses, extranodal lesions. And both diameters are easy to be measured:

(1) Lymph nodes: Lymph nodes should be divided by region; Mediastinal and retroperitoneal lymphadenopathy should be included if present; Measurable lymph nodes should be >1.5 cm in length;

(2) Non-nodal lesions: Including solid organs (such as liver, spleen, kidney, lung, etc.), digestive tract, skin or labeled parts accessible to palpation. Measurable extranodal lesions should be >1.0 cm in length.

Non-measured lesions: Any lesion not selected as measured, dominant disease and truly assessable disease should be considered not measured. Including:

(1) Any lymph nodes/nodal confluent masses, extranodal lesions, i.e., all parts that are not selected as dominant or measurable, or fail to meet the criteria for measurability but are still considered lesions;

(2) Considered as disease involvement but difficult to quantify, such as pleural effusion, ascites, bone metastasis, leptomeningeal involvement, abdominal mass lesions, etc.;

(3) Other undiagnosed lesions requiring imaging follow-up;

Waldeyer's ring and extranodalsites (e.g., gastrointestinal tract, liver, bone marrow): FDG uptake may be higher than the mediastinal pool but not higher than the surrounding background (e.g., the metabolic activity of bone marrow will generally increase due to chemotherapy or application of G-CSF) at the time of CR.

## Annex 2 Eastern Cooperative Oncology Group Performance Status

| Rating | Eastern Cooperative Oncology Group Performance Status |
|---|---|
| 0 | Fully active, able to carry on all pre-disease performance without restriction |
| 1 | Unable to carry out physically strenuous work, able to engage in light or sedentary work, e.g., light indoor work, office work |
| 2 | Able to walk freely and capable of self-care activities in daily life, but unable to carry out any work activities. Able to be ambulant more than 50% of waking hours |
| 3 | Limiting self-care ability in daily life, confined to bed or wheelchair for more than 50% of waking hours |
| 4 | Totally confined to bed. Unable to carry out any self-care activities. Totally confined to bed or chair. |
| 5 | Death |

## Annex 3 International Prognostic Index (IPI) score

| **Item** | **0** | **1** |
|---|---|---|
| Age: | ≤60 years | >60 years |
| Stage | I-II | III-IV |
| ECOG score | 0-1 | ≥2 |
| No extranodal lesions | 0-1 | ≥2 |
| LDH | Normal | Above normal |

## Annex 4 ASCO Guidelines Management of Immune-Related Adverse Events (irAE)

Management of Immune-Related Adverse Events in Patients Treated With Immune Checkpoint Inhibitor Therapy: American Society of Clinical Oncology Clinical Practice Guideline.

Link: https://ascopubs.org/doi/pdf/10.1200/JCO.21.01440

Schneider BJ,et al. Management of Immune-Related Adverse Events in Patients Treated With Immune Checkpoint Inhibitor Therapy: ASCO Guideline Update. J Clin Oncol. 2021 Dec 20;39(36):4073-4126. doi: 10.1200/JCO.21.01440. Epub 2021 Nov 1. Erratum in: J Clin Oncol. 2022 Jan 20;40(3):315.

ASCO **Guidelines**

| Management of Immune-Related Adverse Events in Patients Treated With Immune Checkpoint Inhibitor Therapy: American Society of Clinical Oncology Clinical Practice Guideline | | |
|---|---|---|
| Toxicity Type | Adverse Events | Page |
| 1.0 Skin Toxicity | 1.1 Rash/Inflammatory dermatitis | 6 |
| | 1.2 Bullous dermatoses | 6-7 |
| | 1.3 Severe cutaneous adverse reactions (SCAR) | 7-8 |
| 2.0 Gastrointestinal toxicity | 2.1 Colitis | 9-10 |
| | 2.2 Hepatitis | 10-11 |
| 3.0 Lung Toxicities | 3.1 Pulmonitis | 13 |
| 4.0 Endocrine toxicity | 4.1 Thyroid | 15-16 |
| | 4.1.1 Primary hypothyroidism | 15 |
| | 4.1.2 Hyperthyroidism | 15-16 |
| | 4.2 Adrenalitis - Primary adrenal insufficiency (AI) | 16 |
| | 4.3 Pituitary - hypophysitis | 16-17 |
| | 4.4 Diabetes | 17-18 |
| 5.0 Musculoskeletal toxicity | 5.1 Inflammatory arthritis | 20 |
| | 5.2 Myositis | 20-21 |
| | 5.3 Polymyalgia-like syndrome | 21 |
| 6.0 Renal toxicity | 6.1 Nephritis or acute kidney injury | 23 |
| | 6.2 Follow-up of nephritis or acute kidney injury | 24 |
| 7.0 Nervous system toxicity | 7.1 Myasthenia gravis | 25 |
| | 7.2 Guillain-Barre syndrome | 25-26 |
| | 7.3 Peripheral neuropathy | 26 |

| | | |
|---|---|---|
| | 7.4 Autonomic neuropathy | 26-27 |
| | 7.5 Aseptic meningitis | 27 |
| | 7.6 Encephalitis | 27-28 |
| | 7.7 Demyelinating diseases, including multiple sclerosis, transverse myelitis, acute disseminated encephalomyelitis (ADEM), optic neuritis (ON), and neuromyelitis optica (NMO) | 28 |
| 8.0 Hematologic toxicity | 8.1 Hemolytic anemia | 30 |
| | 8.2 Acquired thrombotic thrombocytopenic purpura | 30-31 |
| | 8.3 Hemolytic uremic syndrome (HUS) | 31 |
| | 8.4 Anemia aplastic aregenerative | 31-32 |
| | 8.5 Lymphopenia | 32 |
| | 8.6 Idiopathic thrombocytopenic purpura | 32-33 |
| | 8.7 Acquired hemophilia | 33 |
| 9.0 Cardiovascular toxicity | 9.1 Myocarditis, pericarditis, arrhythmia, impaired ventricular function with heart failure, vasculitis | 35 |
| | 9.2 Venous thromboembolism | 35-36 |
| 10.0 Ocular toxicities | 10.1 Uveitis, iritis | 37 |
| | 10.2 Scleritis | 37-38 |
| 11.0 Systemic toxicity | 11.1 Infusion reactions | 38 |
| 12.0 Prophylaxis and management of steroid use-related adverse reactions | 12.1 Pretreatment considerations | 39 |
| | 12.2 Prophylaxis of opportunistic infections | 39 |
| | 12.3 Monitoring for acute or short- and long-term adverse reactions | 39 |
| | 12.4 Steroid taper | 39 |


All recommendations are based on expert consensus, the benefits outweigh the risks, and the strength of the recommendations is moderate www.asco.ore/support-care-guidelines© American Society of Clinical Oncology and the 2022 National Comprehensive Cancer Network. All rights reserved.

This guideline is written in collaboration with NCCN.

## Annex 5 US NIAID and FAAN Diagnostic Guidelines for Anaphylaxis

The Anaphylaxis Network guidelines for the National Institute of Allergy and Infectious Diseases (NIAID) and Food Allergy and Anaphylaxis Network (FAAN) define anaphylaxis as a serious allergic reaction that develops rapidly and can lead to death (Sampson et al, 2006 <applewebdata://B361DB79-219E-48F7-A97C-DCE43062FA65#sampson>). They recognize 3 categories of anaphylaxis, with criteria designated to capture from 80% of cases (category 1) to >95% of all cases of anaphylaxis (for all 3 categories).

1. Acute onset of disease (minutes to hours) involving the skin, mucosal tissue, or both (e.g., generalized urticaria, pruritus or flushing, swollen lips-tongue-uvula) with at least one of the following:
    a. Respiratory disturbances (such as dyspnea, wheezing-bronchospasm, stridor, decreased peak expiratory flow rate [PEF], and hypoxic).
    b. Decreased blood pressure (BP) or symptoms associated with end-organ dysfunction (e.g., hypotonia [collapse], syncope, and incontinence).
2. Following exposure to possible antigens, two or more of the following occur rapidly (minutes to hours):
    a. Involvement of the skin-mucosal tissue (e.g., generalized urticaria, swollen lips-tongue-uvula).
    b. Respiratory disturbances (such as dyspnea, wheezing-bronchospasm, stridor, decreased peak expiratory flow rate [PEF], and hypoxic).
    c. Decreased BP or associated symptoms (e.g., hypotonus [collapse], syncope, and incontinence).
    d. Persistent gastrointestinal symptoms (e.g., cramping abdominal pain and vomiting).
3. Following exposure to known antigens, the patient's BP decreases (minutes to hours):
    a. Infants and children: low systolic blood pressure (age-specific) or greater than 30% decrease in low systolic blood pressure.

Adults: systolic blood pressure less than 90 mm Hg or a decrease of more than 30% from the patient's baseline value.

## Annex 6 Bone Marrow Distribution in Adults

| **Site** | | **Bone marrow mass (g)** | **Fraction of red bone marrow Age 40** | **Red marrow mass (g) Age 40** | **% of red bone marrow** | |
|---|---|---|---|---|---|---|
| Skull and lower jaw | Head: | | | 136.6 | | |
| | Skull | 165.8 | 0.75 | 124.3 | 13.1 | 13.1 |
| | Lower jaw | 16.4 | 0.75 | 12.3 | | |
| Humerus, Scapula, Clavicles | Upper limb girdle: | | | 86.7 | | |
| | 2 Humerus, | 26.5 | 0.75 | 20.0 | 8.3 | |
| | Head and neck | | | | | 8.3 |
| | 2 Scapula | 67.4 | 0.75 | 50.5 | | |
| | 2 Clavicles | 21.6 | 0.75 | 16.2 | | |
| Sternebrae and Rib | Sternebrae | 39.0 | 0.6 | 23.4 | 2.3 | |
| | Rib: | | | 82.6 | | |
| | 1 pair | 10.2 | 0.4 in total | 4.1 | | |
| | 2 | 12.6 | | 5.0 | | |
| | 3 | 16.0 | | 6.4 | | |
| | 4 | 18.6 | | 7.4 | | |
| | 5 | 23.8 | | 9.5 | 7.9 | 10.2 |
| | 6 | 23.6 | | 9.4 | | |
| | 7 | 25.0 | | 10.0 | | |
| | 8 | 24.0 | | 9.6 | | |
| | 9 | 21.2 | | 8.5 | | |
| | 10 | 16.0 | | 6.4 | | |
| | 11 | 11.2 | | 4.5 | | |
| | 12 | 4.6 | | 1.8 | | |
| Pelvic bone | Sacrum | 194.0 | 0.75 | 145.6 | 13.9 | |
| | 2 Hip bone | 310.6 | 0.75 | 233.0 | 22.3 | 36.2 |
| Femora | 2 Femoral head and neck | 53.0 | 0.75 | 40.0 | | 3.8 |

## Bone Marrow Distribution in Adults (Continued)

| Site | | Bone marrow mass (g) | Red Bone Marrow Fragment AGE 40 | Red marrow mass (g) AGE 40 | % of red bone marrow | |
|---|---|---|---|---|---|---|
| | Vertebra (cervical): | | | 35.8 | | |
| | 1 | 6.6 | 0.75 in total | 5.0 | | |
| | 2 | 8.4 | | 6.3 | | |
| | 3 | 5.4 | | 4.1 | 3.4 | |
| | 4 | 5.7 | | 4.3 | | |
| | 5 | 5.8 | | 4.4 | | |
| | 6 | 7.0 | | 5.3 | | |
| | 7 | 8.5 | | 6.4 | | |
| | Vertebra (thoracic): | | | 147.9 | | |
| | 1 pair | 10.8 | 0.75 in total | 8.1 | | |
| | 2 | 11.7 | | 8.8 | | |
| | 3 | 11.4 | | 8.5 | | |
| | 4 | 12.2 | | 9.1 | | |
| **vertebra** | 5 | 13.4 | | 10.1 | 14.1 | 28.4 |
| | 6 | 15.3 | | 11.5 | | |
| | 7 | 16.1 | | 12.1 | | |
| | 8 | 18.5 | | 13.9 | | |
| | 9 | 19.7 | | 14.8 | | |
| | 10 | 21.2 | | 15.9 | | |
| | 11 | 21.7 | | 16.3 | | |
| | 12 | 25.0 | | 18.8 | | |
| | Vertebra (lumbar): | | | 114.1 | | |
| | 1 pair | 27.8 | 0.75 in total | 20.8 | | |
| | 2 | 29.1 | | 21.8 | 10.9 | |
| | 3 | 31.8 | | 23.8 | | |
| | 4 | 32.1 | | 24.1 | | |
| | 5 | 31.4 | | 23.6 | | |
| **Total** | | **1497.7** | | **1045.7** | **100.0** | **100.0** |

Note: Data are taken from R.E.ELLIS: The Distribution of Active Bone Marrow in the Adult, Phy. Med. Biol. 5, 255-258, 1961

## Annex 7 Traditional Chinese Medicine (TCM) Prohibited during the Trial

Huatan Huisheng Tablets

Brucea javanica oil soft capsules

Zhemu Syrup

Cantharidin

Cinobufagin

Chan Su

Kangai Injection

Kanglaite

Zhongjiefeng Injection

Aidi Injection

Awei Huapi Gao

Kang Ai Ping Wan

Fukang Capsules

Xiaoaiping

Pingxiao Capsules

Pingxiao Tablets

Shendan Sanjie Capsules

Ankangxin Capsules

Bosheng Ai-ning

Zedoary Turmeric Oil and Glucose Injection

Kanglixin Capsules

Cidan Capsules
