# Supplementary material for: Tifcemalimab as monotherapy or in combination with toripalimab in patients with relapsed/refractory lymphoma: a Phase I trial
Source: Nat Commun. 2025 May 16;16:4559. doi: 10.1038/s41467-025-59461-3 (PMC12084519; doi:10.1038/s41467-025-59461-3)
Supplement: Supplementary file 2 — Reporting Summary [file 41467_2025_59461_MOESM2_ESM.pdf]

nature portfolio

Corresponding author(s): Jun Zhu
Last updated by author(s): Mar 26, 2025

Reporting Summary

Nature Portfolio wishes to improve the reproducibility of the work that we publish. This form provides structure for consistency and transparency in reporting. For further information on Nature Portfolio policies, see our Editorial Policies and the Editorial Policy Checklist.

Statistics

For all statistical analyses, confirm that the following items are present in the figure legend, table legend, main text, or Methods section.

n/a	Confirmed	
☐	☒	The exact sample size (*n*) for each experimental group/condition, given as a discrete number and unit of measurement
☐	☒	A statement on whether measurements were taken from distinct samples or whether the same sample was measured repeatedly
☒	☐	The statistical test(s) used AND whether they are one- or two-sided<br>*Only common tests should be described solely by name; describe more complex techniques in the Methods section.*
☒	☐	A description of all covariates tested
☒	☐	A description of any assumptions or corrections, such as tests of normality and adjustment for multiple comparisons
☐	☒	A full description of the statistical parameters including central tendency (e.g. means) or other basic estimates (e.g. regression coefficient) AND variation (e.g. standard deviation) or associated estimates of uncertainty (e.g. confidence intervals)
☒	☐	For null hypothesis testing, the test statistic (e.g. *F*, *t*, *r*) with confidence intervals, effect sizes, degrees of freedom and *P* value noted<br>*Give P values as exact values whenever suitable.*
☒	☐	For Bayesian analysis, information on the choice of priors and Markov chain Monte Carlo settings
☒	☐	For hierarchical and complex designs, identification of the appropriate level for tests and full reporting of outcomes
☒	☐	Estimates of effect sizes (e.g. Cohen's *d*, Pearson's *r*), indicating how they were calculated

Our web collection on statistics for biologists contains articles on many of the points above.

Software and code

Policy information about availability of computer code

Data collection	Medidata Rave Platform
Data analysis	SAS 9.4 was employed to calculate descriptive statistics, while PK parameters were derived using WinNonlin Version 8.3 (Certara) software.

For manuscripts utilizing custom algorithms or software that are central to the research but not yet described in published literature, software must be made available to editors and reviewers. We strongly encourage code deposition in a community repository (e.g. GitHub). See the Nature Portfolio guidelines for submitting code & software for further information.

Data

Policy information about availability of data

All manuscripts must include a data availability statement. This statement should provide the following information, where applicable:

- Accession codes, unique identifiers, or web links for publicly available datasets
- A description of any restrictions on data availability
- For clinical datasets or third party data, please ensure that the statement adheres to our policy

The study protocol was provided as a Supplementary Note 2. All requests for individual participant data will be reviewed by the leading clinical site, Peking University Cancer Hospital & Institute, and the study sponsor, Shanghai Junshi Biosciences, to verify whether the request is subject to any patient privacy, intellectual property or confidentiality obligations. Requests for access to the patient-level data from this study for research purposes can be submitted via email to zhu-jun2017@outlook.com with detailed proposals for approval. A signed data access agreement with the sponsor is required before accessing shared data. Access

is provided after a proposal has been approved by an independent review committee identified for this purpose and after receipt of a signed data sharing agreement. Access to all individual participant data collected during the trial, will be provided after anonymization. Data and documents will be provided in a secure data sharing environment. No expiration date of data requests is currently set once data are made available. Source data are provided with this paper. All remaining data can be found in the Article, Supplementary and Source Data files.

Research involving human participants, their data, or biological material

Policy information about studies with human participants or human data. See also policy information about sex, gender (identity/presentation), and sexual orientation and race, ethnicity and racism.

Reporting on sex and gender	Sex and/or gender were not considered in study design, and which was determined based on self-reporting.
Reporting on race, ethnicity, or other socially relevant groupings	This study was conducted in china, and all patients who met the inclusion criteria could enter the screening process.
Population characteristics	In Part A (N=25), the median age was 45 (range: 26 to 70) years. The majority of the patients had Stage III/IV disease (96.0%) and an ECOG PS of 0 (84.0%). The median prior anti-tumor treatment line was 4, and 13 (52.0%) patients received prior PD-(L)1 blockade. There were 9 (36.0%) patients with cHL, and all of them received prior PD-(L)1 blockade, and the median prior anti-tumor treatment line was 6 among the cHL patients.<br>In Part B (N=46), the median age was 36 (range: 19 to 68) years. The majority of the patients had Stage III/IV disease (82.6%) and an ECOG PS of 0 (56.5%). The median prior anti-tumor treatment line was 3, and 41 (89.1%) patients received prior PD-(L)1 blockade. There were 42 patients (91.3%) with cHL, and the median prior anti-tumor treatment line was 3.5 among those cHL patients, with 41 of them receiving prior PD-(L)1 blockade. Furthermore, among all the cHL patients, 34 patients were refractory to PD-(L)1 blockade (defined as PD occurring during treatment or within 3 months after the last dose), 28 patients had PD-1 blockade as their most recent therapy, and 21 patients received at least 2 courses of PD-(L)1 blockades.
Recruitment	The investigators and sponsor judged whether a patient met the eligibility criteria. Written informed consent was obtained from all patients. Patients volunteered to participate in the study.
Ethics oversight	The protocol and all amendments were approved by the ethics committee at each study site, including: Peking University Cancer Hospital & Institute; Harbin Institute of Hematology & Oncology; Tianjin Medical University Cancer Institute and Hospital; The First Affiliated Hospital of Guangxi Medical University; Jiangxi Cancer Hospital; The First Affiliated Hospital of Nanchang University; Henan Cancer Hospital; Zhejiang Cancer Hospital; West China Hospital of Sichuan University; The Second Hospital of Dalian Medical University; Shanghai Ruijin Hospital, Shanghai Jiao Tong University School of Medicine; The Fifth Medical Center of the General Hospital of the Chinese People's Liberation Army; The Affiliated Cancer Hospital of Guizhou Medical University; Hunan Cancer Hospital.<br>The study was overseen by the Sponsor and the ethics committee at each study site.

Note that full information on the approval of the study protocol must also be provided in the manuscript.

Field-specific reporting

Please select the one below that is the best fit for your research. If you are not sure, read the appropriate sections before making your selection.

☒ Life sciences ☐ Behavioural & social sciences ☐ Ecological, evolutionary & environmental sciences

For a reference copy of the document with all sections, see nature.com/documents/nr-reporting-summary-flat.pdf

Life sciences study design

All studies must disclose on these points even when the disclosure is negative.

Sample size	This was an early phase study, and the sample size calculation was not based on a statistical hypothesis. Approximately 170 patients were planned to be enrolled into the study. The sample size for indication expansion might be adjusted based on the efficacy and safety data obtained during the study.
Data exclusions	No data were excluded from the analyses.
Replication	As an early phase clinical trial, no replication is required.
Randomization	This phase 1 study was designed to explore the safety and preliminary efficacy of tifcemalimab with or without toripalimab in patients with relapsed or refractory lymphoma. As no control group was set, randomization was not applicable.
Blinding	This phase 1 study was designed to explore the safety and preliminary efficacy of tifcemalimab with or without toripalimab in patients with relapsed or refractory lymphoma. Open-Label design was used to better ensure patients' safety, so blinding were not applicable.

Reporting for specific materials, systems and methods

We require information from authors about some types of materials, experimental systems and methods used in many studies. Here, indicate whether each material, system or method listed is relevant to your study. If you are not sure if a list item applies to your research, read the appropriate section before selecting a response.

Materials & experimental systems

n/a	Involved in the study
☐	☒ Antibodies
☒	☐ Eukaryotic cell lines
☒	☐ Palaeontology and archaeology
☒	☐ Animals and other organisms
☐	☒ Clinical data
☒	☐ Dual use research of concern
☒	☐ Plants

Methods

n/a	Involved in the study
☒	☐ ChIP-seq
☒	☐ Flow cytometry
☒	☐ MRI-based neuroimaging

Antibodies

Antibodies used	Antibodies used in this study included tifcemalimab and toripalimab, both were manufactured and supplied by the sponsor, Shanghai Junshi Biosciences.
Validation	The serum concentrations of tifcemalimab and toripalimab were determined using a validated immunoassay method for PK analysis.

Clinical data

Policy information about clinical studies

All manuscripts should comply with the ICMJE guidelines for publication of clinical research and a completed CONSORT checklist must be included with all submissions.

Clinical trial registration	NCT04477772
Study protocol	Study protocol is provided as a appendix for the article.
Data collection	Data were collected from 14 cancer hospitals or general hospitals in china.
Outcomes	The primary endpoints were to evaluate safety and to determine the RP2D of tifcemalimab as monotherapy and in combination with toripalimab. Secondary endpoints were to evaluate clinical efficacy (include objective response rate [ORR], duration of response [DoR], disease control rate [DCR], progression-free survival [PFS] and overall survival [OS]), and the pharmacokinetics of tifcemalimab as monotherapy and in combination with toripalimab.<br>Dose-limiting toxicities (DLTs) were to be collected for 21 days after the first dose of the study treatment during dose escalation. All adverse events (AEs) including serious adverse events (SAEs) were captured through 90 days after the last dose of study treatments or until initiation of a new anti-tumor therapy, whichever occurred first. AEs were graded according to the National Cancer Institute-Common Terminology Criteria for Adverse Events (NCI-CTCAE) version 5.0.<br>Tumor status was evaluated by the investigators per 2014 Lugano criteria every 9 weeks (± 7 days) after the first dose of study treatment until disease progression, initiation of new anti-tumor therapy, withdrawal of consent, lost to follow-up or death, whichever occurred first. Tumor imaging examination methods included enhanced computed tomography (CT) or magnetic resonance imaging (MRI). Baseline FDG-PET examination was required within 28 days before enrollment. During the treatment, FDG-PET examination was to be performed within 4 weeks when complete response (CR) was assessed by imaging such as CT or MRI.<br>Biological sample collection for pharmacokinetics (PK) and BTLA receptor occupancy was detailed in the study protocol.

Plants

Seed stocks	*Report on the source of all seed stocks or other plant material used. If applicable, state the seed stock centre and catalogue number. If plant specimens were collected from the field, describe the collection location, date and sampling procedures.*
Novel plant genotypes	*Describe the methods by which all novel plant genotypes were produced. This includes those generated by transgenic approaches, gene editing, chemical/radiation-based mutagenesis and hybridization. For transgenic lines, describe the transformation method, the number of independent lines analyzed and the generation upon which experiments were performed. For gene-edited lines, describe the editor used, the endogenous sequence targeted for editing, the targeting guide RNA sequence (if applicable) and how the editor was applied.*
Authentication	*Describe any authentication procedures for each seed stock used or novel genotype generated. Describe any experiments used to assess the effect of a mutation and, where applicable, how potential secondary effects (e.g. second site T-DNA insertions, mosiacism, off-target gene editing) were examined.*
